# Prognostic and Predictive Value of Immune-Related Gene Expression Signatures vs Tumor-Infiltrating Lymphocytes in Early-Stage ERBB2/HER2-Positive Breast Cancer: A Correlative Analysis of the CALGB 40601 and PAMELA Trials

**DOI:** 10.1001/jamaoncol.2022.6288

**Published:** 2023-01-05

**Authors:** Aranzazu Fernandez-Martinez, Tomás Pascual, Baljit Singh, Paolo Nuciforo, Naim U. Rashid, Karla V. Ballman, Jordan D. Campbell, Katherine A. Hoadley, Patricia A. Spears, Laia Pare, Fara Brasó-Maristany, Nuria Chic, Ian Krop, Ann Partridge, Javier Cortés, Antonio Llombart-Cussac, Aleix Prat, Charles M. Perou, Lisa A. Carey

**Affiliations:** 1 Lineberger Comprehensive Cancer Center, University of North Carolina, Chapel Hill; 2 Department of Genetics, University of North Carolina, Chapel Hill; 3 Department of Medical Oncology, Hospital Clínic de Barcelona, Barcelona, Spain; 4 Translational Genomics and Targeted Therapeutics in Solid Tumors, August Pi i Sunyer Biomedical Research Institute (IDIBAPS), Barcelona, Spain; 5 SOLTI Breast Cancer Cooperative Group, Barcelona, Spain; 6 Department of Pathology, White Plains Hospital, White Plains, New York; 7 Molecular Oncology Laboratory, Vall d’Hebron Institute of Oncology, Barcelona, Spain; 8 Department of Biostatistics, University of North Carolina, Chapel Hill; 9 Alliance Statistics and Data Management Center, Mayo Clinic, Rochester, Minnesota; 10 Reveal Genomics, Barcelona, Spain; 11 Yale Cancer Center, New Haven, Connecticut; 12 Department of Breast Oncology, Dana-Farber Cancer Institute, Boston, Massachusetts; 13 International Breast Cancer Center, Barcelona, Spain; 14 Medical Oncology Department, Hospital Arnau de Vilanova, Valencia, Spain; 15 Department of Medicine, University of Barcelona, Barcelona, Spain; 16 Breast Cancer Unit, IOB-QuirónSalud, Barcelona, Spain; 17 Division of Medical Oncology, Department of Medicine, School of Medicine, University of North Carolina at Chapel Hill, Chapel Hill

## Abstract

**Importance:**

Both tumor-infiltrating lymphocytes (TILs) assessment and immune-related gene expression signatures by RNA profiling predict higher pathologic complete response (pCR) and improved event-free survival (EFS) in patients with early-stage ERBB2/HER2-positive breast cancer. However, whether these 2 measures of immune activation provide similar or additive prognostic value is not known.

**Objective:**

To examine the prognostic ability of TILs and immune-related gene expression signatures, alone and in combination, to predict pCR and EFS in patients with early-stage ERBB2/HER2-positive breast cancer treated in 2 clinical trials.

**Design, Setting, and Participants:**

In this prognostic study, a correlative analysis was performed on the Cancer and Leukemia Group B (CALGB) 40601 trial and the PAMELA trial. In the CALGB 40601 trial, 305 patients were randomly assigned to weekly paclitaxel with trastuzumab, lapatinib, or both for 16 weeks. The primary end point was pCR, with a secondary end point of EFS. In the PAMELA trial, 151 patients received neoadjuvant treatment with trastuzumab and lapatinib for 18 weeks. The primary end point was the ability of the HER2-enriched subtype to predict pCR. The studies were conducted from October 2013 to November 2015 (PAMELA) and from December 2008 to February 2012 (CALGB 40601). Data analyses were performed from June 1, 2020, to January 1, 2022.

**Main Outcomes and Measures:**

Immune-related gene expression profiling by RNA sequencing and TILs were assessed on 230 CALGB 40601 trial pretreatment tumors and 138 PAMELA trial pretreatment tumors. The association of these biomarkers with pCR (CALGB 40601 and PAMELA) and EFS (CALGB 40601) was studied by logistic regression and Cox analyses.

**Results:**

The median age of the patients was 50 years (IQR, 42-50 years), and 305 (100%) were women. Of 202 immune signatures tested, 166 (82.2%) were significantly correlated with TILs. In both trials combined, TILs were significantly associated with pCR (odds ratio, 1.01; 95% CI, 1.01-1.02; *P* = .02). In addition to TILs, 36 immune signatures were significantly associated with higher pCR rates. Seven of these signatures outperformed TILs for predicting pCR, 6 of which were B-cell related. In a multivariable Cox model adjusted for clinicopathologic factors, including PAM50 intrinsic tumor subtype, the immunoglobulin G signature, but not TILs, was independently associated with EFS (immunoglobulin G signature–adjusted hazard ratio, 0.63; 95% CI, 0.42-0.93; *P* = .02; TIL-adjusted hazard ratio, 1.00; 95% CI, 0.98-1.02; *P* = .99).

**Conclusions and Relevance:**

Results of this study suggest that multiple B-cell–related signatures were more strongly associated with pCR and EFS than TILs, which largely represent T cells. When both TILs and gene expression are available, the prognostic value of immune-related signatures appears to be superior.

## Key Points

**Question:**

Which immune-related biomarker provides the most valuable information to predict pathologic complete response and event-free survival in patients with early-stage ERBB2/HER2-positive breast cancer: tumor-infiltrating lymphocytes, immune-related gene expression signatures, or both?

**Findings:**

In this predictive prognostic study in which a combined correlative analysis of the CALGB 40601 and PAMELA trials was conducted, 305 patients with early-stage ERBB2/HER2-positive breast cancer, 6 B-cell–related signatures were more strongly associated with pathologic complete response than were tumor-infiltrating lymphocytes. In a multivariable Cox model performed in the CALGB 40601 trial, the immunoglobulin G signature, but not tumor-infiltrating lymphocytes, was independently associated with event-free survival.

**Meaning:**

Findings suggest that when both tumor-infiltrating lymphocytes and gene expression are available, the prognostic and predictive value of RNA sequencing–based immune signatures is superior.

## Teaser

This prognostic study (in which a combined correlative analysis of the CALGB 40601 and PAMELA trials was conducted) compares the effectiveness of tumor-infiltrating lymphocytes vs immune-related gene expression signatures in predicting pathologic complete response and event-free survival in patients with early-stage ERBB2/HER2-positive breast cancer.

## Introduction

During the last 2 decades, the outcome of patients with early-stage ERBB2/HER2-positive breast cancer has markedly improved owing to new treatment strategies combining polychemotherapy and multiple ERBB2/HER2-targeted drugs.^1,2,3,4,5,6,7,8,9,10^ However, it is increasingly evident that many patients are overtreated by the recommended regimens, whereas others still experience metastatic relapse. A primary research focus in breast cancer is to better tailor treatments to risk; to accomplish this, effective prognostic and predictive biomarkers are needed.

Increasing evidence suggests that the activation of the host immune system mediates the response to ERBB2/HER2-targeted therapies in breast cancer.^11^ Currently, there are several methods to assess intratumor immune activation. The presence of tumor-infiltrating lymphocytes (TILs) in the hematoxylin-eosin–stained tumor slides is one of these methods, and an international working group has established standardized tools for measuring TILs.^12^ The percentage of TILs that infiltrate the breast tumor is positively prognostic in patients with early-stage ERBB2/HER2-positive breast cancer treated with anti-ERBB2/HER2 therapies in multiple scenarios: in the neoadjuvant and adjuvant setting, in the presence or absence of chemotherapy, with single and dual ERBB2/HER2 blockade, and when assessed at baseline and during treatment.^13,14,15,16,17,18^ Other than TILs, immune activation can also be measured by gene expression.^19,20^ In patients with early-stage ERBB2/HER2-positive breast cancer treated in the neoadjuvant setting, immune-related gene expression signatures (iGESs) are associated with higher pathologic complete response (pCR) rates and prolonged survival.^21,22,23^ Specifically, the immunoglobulin G (IgG) signature^24^ has previously shown strong and independent prognostic value across many studies.^2,19,22,25^ However, the comparative prognostic ability of these different means of measuring immune activation has not been well examined. In this retrospective predictive and prognostic study, we tested which biomarker, or combination of biomarkers, is the most powerful for response and survival in 2 independent clinical trials: the Cancer and Leukemia Group B (CALGB) 40601 trial (NCT00770809) and the PAMELA trial (NCT01973660), respectively. The CALGB is now part of the Alliance for Clinical Trials in Oncology.

## Methods

### Neoadjuvant Trials

The CALGB 40601 trial study design, pCR, event-free survival (EFS), overall survival, and genomic correlative studies have been previously published.^2,22^ In this predictive and prognostic study, a total of 305 women with stage II to III ERBB2/HER2-positive breast cancer were randomly assigned to receive neoadjuvant weekly paclitaxel with the addition of trastuzumab, lapatinib, or both for 16 weeks. The primary end point was pCR, defined as no invasive tumor in the breast at surgery, and secondary end points included EFS. The PAMELA trial study design, pCR, and biomarker correlative studies have also been previously published.^14,26,27^ In this phase 2 trial, 151 patients with stage I to IIIA ERBB2/HER2-positive breast cancer received neoadjuvant lapatinib plus trastuzumab for 18 weeks. The primary outcome was the ability of the HER2-enriched subtype to predict pCR, defined as no invasive tumor in the breast at surgery. Each trial participant signed an institutional review board–approved (National Cancer Institute Central institutional review board for the CALGB 40601 trial and Hospital Universitari Vall d’Hebron for the PAMELA trial), protocol-specific informed consent document following federal and institutional guidelines.

### Tumor Gene Expression Analyses and iGESs

Gene expression profiles from pretreatment core biopsies were obtained from 264 of 305 CALGB 40601 trial participants (86.6%) and 142 of 151 PAMELA trial participants (94.0%) (eFigure 1 in the Supplement). Whole-transcriptome analyses by messenger RNA sequencing (RNA-Seq) were performed in the University of North Carolina High-Throughput Sequencing Facility and analyzed by the university’s Lineberger Comprehensive Cancer Center Bioinformatics Core. The RNA sequencing libraries were made from total RNA with the TruSeq (Illumina) messenger RNA kit in the CALGB 40601 trial and the TruSeq RNA Access kit in the PAMELA trial and were sequenced on an Illumina HiSeq 2000 using a 2 × 50–base pair configuration. The CALGB 40601 trial RNA-Seq FASTQ files are available on the dbGAP repository (phs001570.v3.p1). The PAMELA trial RNA-Seq FASTQ files are available on EGA (EGAS00001006410/EGAD00001009054). Purity-filtered reads were aligned to the human reference GRCh38/hg38 genome, using Spliced Transcripts Aligned to a Reference, version 2.4.2a.^28^ Transcript (GENCODE, version 22) abundance estimates were generated by Salmon, version 0.6.0^29^ in “-quant” mode, based on the Spliced Transcripts Aligned to a Reference alignments. Raw read counts for all RNA-Seq samples were normalized to a fixed upper quartile.^30^ Messenger RNA sequencing–normalized gene counts were then log_2_ transformed, and genes were filtered for those expressed in 70% of samples. The batch effect between the gene expression from the CALGB 40601 and PAMELA trials was corrected by applying the distance-weighted discrimination method,^31,32^ version 1.0.2^33^ (SlicerSALT) and using the CALGB 40601 trial as reference. Intrinsic subtypes were obtained from RNA-Seq gene expression data as described elsewhere.^22^

Expression of 202 iGESs from 43 publications (eReferences in the Supplement) was calculated. The list of iGESs and the genes within each signature are summarized in eTable 1 in the Supplement, and the R code is provided.^34^ Finally, the iGESs were classified into 22 immune classes based on their gene ontology, with CIBERSORT as reference.^35^

### TIL Evaluation

In the CALGB 40601 and PAMELA trials, slides from core biopsies were available for 230 of 264 patients (87.1%) and 138 of 142 patients (97.2%) from the RNA-Seq cohort (eFigure 1 in the Supplement), respectively. The stromal TILs from both clinical trials were scored by the CALGB 40601 trial lead study pathologist (B.S.), following the International TILs Working Group recommendations.^12^ In patients with more than 1 core biopsy available, the mean of the 2 TILs assessments was calculated, and patients without TILs assessment were censored.

### Statistical Analysis

The criteria of the Reporting Recommendations for Tumour Marker Prognostic Studies (REMARK) guidelines were followed for this study.^36^ Comparisons of differences in baseline clinicopathologic variables between the CALGB 40601 trial and the PAMELA trial were made with the Wilcoxon rank sum test (continuous variables) and the χ^2^ test (categorical variables).

For pCR and EFS modeling, the iGES scores were analyzed as continuous variables. Stromal TILs were analyzed as continuous and discrete variables with different prespecified cutoffs (ie, 20%, 40%, and 60%). Immune-related gene expression signatures and TILs were also categorized by tertiles (ie, low, medium, and high) for visualization purposes.

The association between TILs and iGESs was measured with Spearman correlation coefficients. For differential gene expression analysis, we performed a multiclass significance analysis of microarrays.^37^

The association of immune biomarkers with pCR was evaluated in the CALGB 40601 and PAMELA combined cohort by logistic regression models. *P* values were adjusted for multiple testing with a Benjamini-Hochberg method to control the false discovery rate. To compare the goodness of fit of 2 models, we used the Akaike information criterion (AIC). As accuracy metric, we calculated the area under the receiver operating characteristic curve (AUC) for pCR, using the CALGB 40601 trial as a train set and the PAMELA trial as validation. In the CALGB 40601 trial, a mean of the AUC was calculated with 10-fold cross-validation.

In the CALGB 40601 trial, EFS was defined as the time from randomization to a breast cancer relapse after surgery, second primary malignant neoplasm, or death without recurrence for women who underwent surgery. For individuals who did not undergo surgery, the event was defined as death during clinical follow-up or noncompletion of neoadjuvant therapy due to progressive disease. The median follow-up was 9.1 years (IQR, 8.10-9.84). The association of immune biomarkers with EFS was evaluated with Cox regression models. *P* values were adjusted for multiple testing with a Benjamini-Hochberg correction. To compare the goodness of fit, we used the AIC. To evaluate the accuracy, we calculated an average C index using 5-fold cross-validation. To avoid a potential guarantee time bias in the multivariable EFS models including pCR status, we performed a 30-week landmark analysis. The landmark subpopulation included only patients without events who were followed up at 30 weeks after randomization.^38,39^ Finally, to compare the prognostic ability of 2 nested models, we used the likelihood ratio test (LRT).

All the analyses were based on the study clinical database frozen on June 10, 2021. All tests were 2-sided, and a .05 level of significance was used. All statistical analyses were performed with R version 3.5.2 (R Foundation for Statistical Computing) and Python version 3.6 (Python Software Foundation). Data analyses were performed from June 1, 2020, to January 1, 2022.

## Results

### Baseline Patient Characteristics and TIL Distribution

The characteristics of the 305 patients included in the study are summarized in Table 1. Data on race and ethnicity were collected in the CALGB 40601 trial but not the PAMELA trial; therefore, we decided not to include the information in this study. The median age of the patients was 50 years (IQR, 42-50 years), and 305 (100%) were women. Patients enrolled in the CALGB 40601 trial were significantly younger, more likely to be premenopausal, and at a more advanced clinical stage at diagnosis than those enrolled in the nonchemotherapy PAMELA trial. There were no significant differences between the trials in the hormone receptor status and intrinsic subtype distribution. In the CALGB 40601 trial, there were no statistically significant differences in the baseline clinicopathologic characteristics between the TIL cohort (n = 230) and the landmark cohort (n = 227) (eTable 2 in the Supplement).

**Table 1.** Baseline Characteristics of Patients From the Study Population by Clinical Trial

Characteristic	Patients, No. (%)			*P* value^a^
	CALGB 40601 trial (n = 230)	PAMELA trial (n = 138)	All (N = 368)	
Age, median (IQR), y	49 (41-56)	54 (44-64)	50 (42-59)	
Menopause status				
Postmenopausal	89 (38.7)	81 (58.7)	170 (46.2)	<.001
Premenopausal	141 (61.3)	57 (41.3)	198 (53.8)	
Hormone receptor status				
Negative	93 (40.4)	67 (48.6)	160 (43.5)	.13
Positive	137 (59.6)	71 (51.4)	208 (56.5)	
Clinical stage				
I	0	45 (32.6)	45 (12.2)	<.001
II	157 (68.3)	86 (62.3)	242 (65.8)	
III	73 (31.7)	8 (5.8)	81 (22.0)	
Treatment				
HL ± ET	0	138 (100)	138 (37.5)	<.001
TH	89 (38.7)	0	89 (24.2)	
THL	95 (41.3)	0	95 (25.8)	
TL	46 (20.0)	0	46 (12.5)	
Intrinsic subtype				
Basal-like	19 (8.3)	7 (5.1)	26 (7.1)	.06
HER2-enriched	131 (57.0)	91 (65.9)	222 (60.3)	
Luminal A	26 (11.3)	20 (14.5)	46 (12.5)	
Luminal B	32 (13.9)	16 (11.6)	48 (13.0)	
Normal-like	22 (9.5)	4 (2.9)	26 (7.1)	

Abbreviations: CALGB, Cancer and Leukemia Group B 40601 trial; ET, endocrine therapy; HL, trastuzumab plus lapatinib; TH, weekly paclitaxel plus trastuzumab; THL, TH plus lapatinib; TL, weekly paclitaxel plus lapatinib.

^a^ Statistical differences were assessed with the Wilcoxon rank sum test (for age) and the Pearson χ^2^ test (for the rest of the variables).

**Figure 1.**
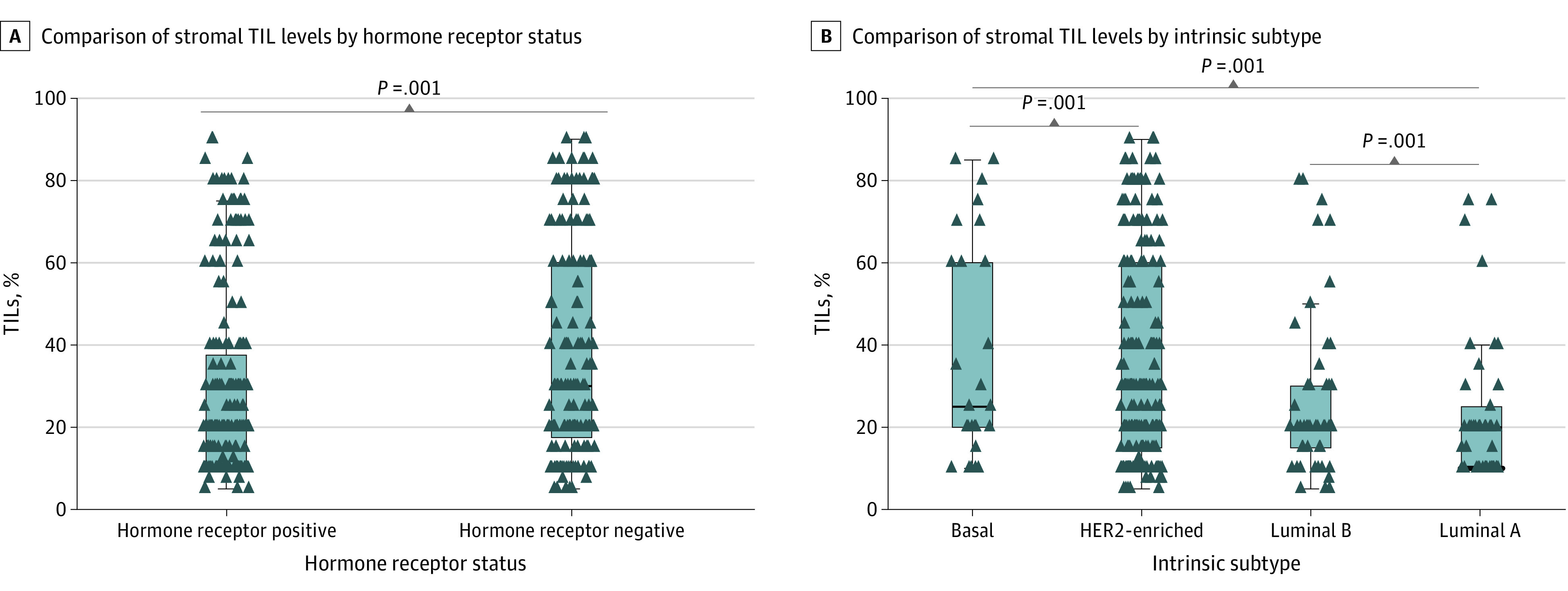
Comparison of Stromal Tumor-Infiltrating Lymphocyte (TIL) Levels by Hormone Receptor Status (A) and Intrinsic Subtype (B) in the Combined Cohort of the Cancer and Leukemia Group B 40601 Trial and the PAMELA Trial Statistical differences were assessed with the Kruskal-Wallis test. The horizontal line in each box plot indicates the median of the distribution.

In the CALGB 40601 trial, the median TIL count was 20% (IQR, 13.1%-45%) (eFigure 2A in the Supplement). In the PAMELA trial, the median TIL count was 30% (IQR, 20%-50%) (eFigure 2B in the Supplement); the TILs distribution was not significantly different between the studies (eFigure 2C in the Supplement). The proportion of TILs was significantly higher in hormone receptor–negative compared with hormone receptor–positive disease in the CALGB 40601 trial (median in hormone receptor–negative disease = 30 [IQR, 15-60]; median in hormone receptor–positive disease = 20 [IQR, 10-35]; *P* = .03), the PAMELA trial (median in hormone receptor–negative disease = 30 [IQR, 20-65]; median in hormone receptor–positive disease = 20 [IQR, 10-35]; *P* = .04), and the combined cohort (median in hormone receptor–negative disease = 30 [IQR, 19-60]; median in hormone receptor–positive disease = 20 [IQR, 10-36]; *P* = .001) (eFigure 3A and C in the Supplement; Figure 1A). There was a significant difference in the proportion of TILs by tumor intrinsic subtype, with a significantly higher proportion of TILs in nonluminal (ie, basal-like and HER2-enriched) compared with luminal tumors (CALGB 40601 trial: median nonluminal tumors = 25 [IQR, 15-60], median luminal tumors = 20 [IQR, 10-30], *P* = .01; PAMELA trial: median nonluminal tumors = 30 [IQR, 20-60], median luminal tumors = 20 [IQR, 10-30], *P* = .004; combined cohort: median nonluminal tumors = 30 [IQR, 15-60], median luminal tumors = 20 [IQR, 10-30], *P* < .001) (eFigure 3B and D in the Supplement; Figure 1B).

### Association Between TILs and iGESs

We found that 166 of 202 iGESs (82.2%) were significantly correlated with TILs in both studies, 179 in the CALGB 40601 trial and 174 in the PAMELA trial (eTable 3 in the Supplement). Spearman correlation coefficients are summarized in eTable 3 in the Supplement, and the coefficients from the 20 signatures that were the most correlated with TILs in both studies are represented in eFigure 4 in the Supplement. The iGESs most correlated with TILs were largely T-cell related. The highest Spearman correlation coefficient for TILs was 0.61 in the CALGB 40601 trial and 0.71 in the PAMELA trial. Three signatures associated with resistance to immunotherapy, inflammation, and immunosuppression were significantly negatively correlated with TILs in both studies (Spearman correlation coefficients for the CALGB 40601 and PAMELA trials: −0.29 and −0.37, −0.26 and −0.22, and −0.17 and −0.23, respectively) (eFigure 5 and eTable 3 in the Supplement).

**Figure 2.**
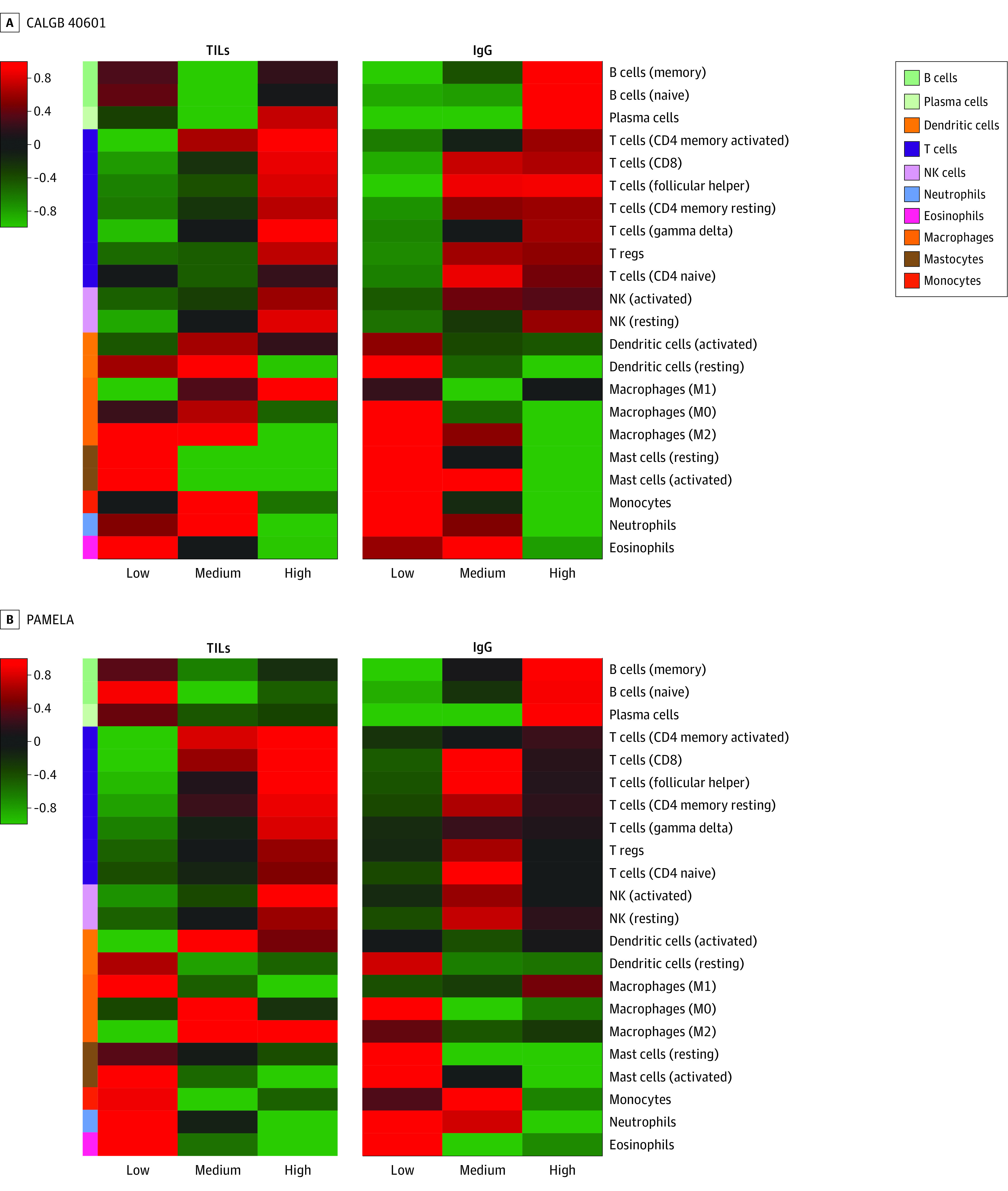
Heatmap Representing the Different Distributions of the CIBERSORT-Derived Gene Expression Signatures by Tumor-Infiltrating Lymphocyte (TIL) and Immunoglobulin G (IgG) Levels Cancer and Leukemia Group B 40601 (CALGB 40601) and PAMELA trial samples were classified into 3 different groups by study, depending on the TIL and IgG levels by tertiles (ie, low, medium, and high TILs; and low, medium, and high IgG). Then, a multiclass significance analysis of microarrays was performed. The standardized mean differences between the immune signatures in 1 class vs the overall mean expression for each study are represented in 4 heatmaps. NK indicates natural killer; T regs, T regulatory cells.

To further study the association between TILs and iGESs, we compared the differences in immune cell infiltration, using our CIBERSORT-derived signatures to perform a multiclass significance analysis of microarrays by TIL levels (ie, low, medium, and high) and by IgG signature levels (ie, low, medium, and high) in the CALGB 40601 and PAMELA trials. The standardized mean differences between the iGESs in 1 class vs the overall mean expression are represented in Figure 2A (the CALGB 40601 trial) and Figure 2B (the PAMELA trial). Tumors with high TIL and IgG levels were significantly enriched for T cells compared with those with low TIL and IgG levels. However, although tumors with high IgG levels were enriched for B-cell and plasma cell signatures compared with those with low levels, in both studies, samples with high and low TIL levels showed a high expression of B-cell and plasma cell signatures. This analysis suggests that TILs do not recapitulate B-cell and plasma cell immune infiltration, and the 2 biomarkers should not be considered the same.

### Association of TILs and iGESs With pCR in the CALGB 40601 and PAMELA Trials

**Figure 3.**
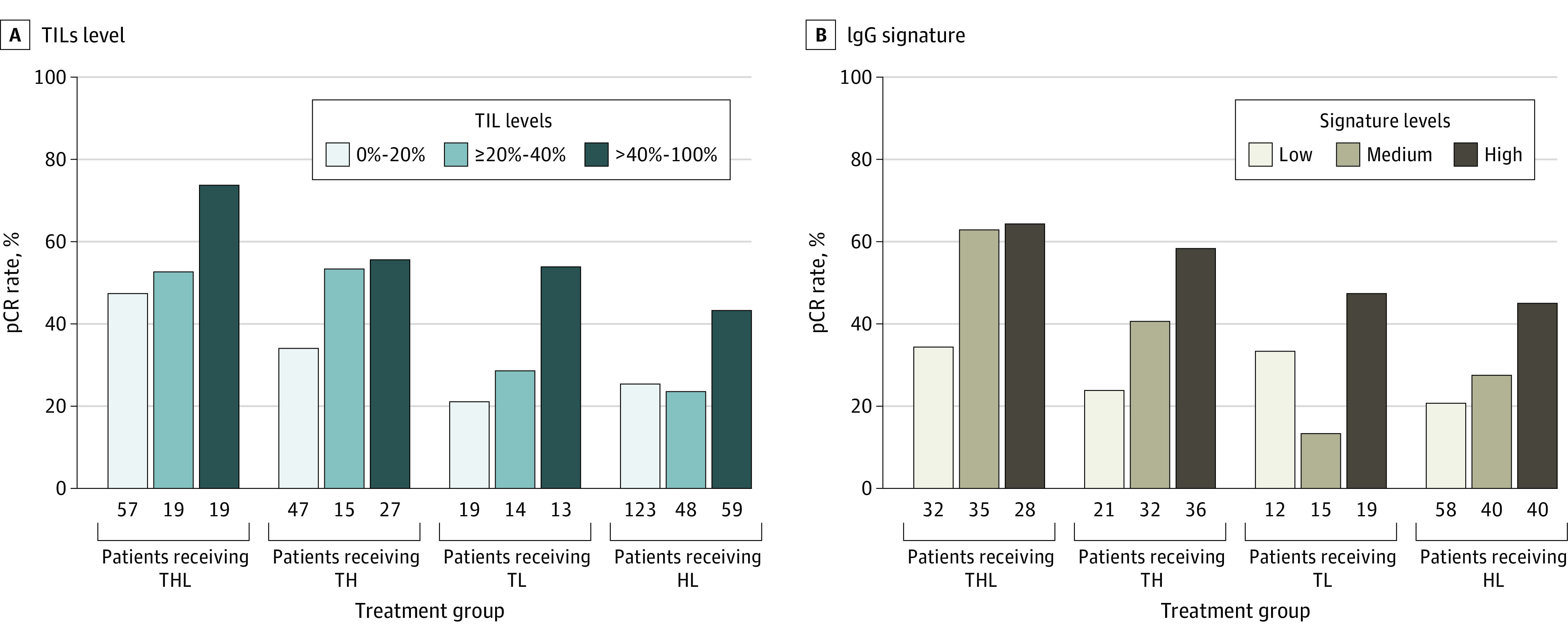
Rates of Pathologic Complete Response (pCR) According to Levels of Tumor-Infiltrating Lymphocytes (TILs) (A) and Immunoglobulin G (IgG) Gene Expression Signature (B) Both variables were divided by tertiles to illustrate their association with pCR. HL indicates trastuzumab plus lapatinib; TH, weekly paclitaxel plus trastuzumab; THL, TH plus lapatinib; and TL, weekly paclitaxel plus lapatinib.

In the combined cohort, the percentage of TILs as a continuous variable was significantly associated with pCR, with an odds ratio of 1.01 (95% CI, 1.01-1.02; *P* = .02) for each 1% increase in TILs. This association was observed regardless of the clinical trial and treatment group (Figure 3A). High vs low levels of TILs using a cutoff of 20% and 40% were also significantly associated with pCR (20%: odds ratio, 1.86; 95% CI, 1.20-2.91; *P* = .04; 40%: odds ratio, 2.29; 95% CI, 1.40-3.77; *P* = .02). The model including TILs with a cutoff of 40% rather than TILs as a continuous variable better predicted pCR (AIC, 471.68 for TILs with a cutoff of 40% vs 472.23 for TILs as a continuous variable; AUC, 0.59 for TILs with a cutoff of 40% in the PAMELA trial vs 0.57 for TILs as a continuous variable).

Thirty-six of 202 iGESs (17.8%) were also significantly associated with pCR independently of the treatment group and study (eTable 4 in the Supplement; Figure 3B; eFigure 6 in the Supplement). The biomarker models from 13 of these signatures better predicted the response end point than the best TILs model for pCR prediction, showing lower AIC values (AIC, 450.06-471.30 for iGESs vs 471.68 for TILs with a cutoff of 40%) (eTable 4 in the Supplement). When the accuracy metrics were examined, 7 iGESs outperformed TILs for pCR, showing higher AUC values in the train (CALGB 40601 trial) and the validation set (PAMELA trial) (eTable 5 in the Supplement). Moreover, in multivariable models adjusted by multiple clinical parameters (study, treatment group, stage, age, hormone receptor status, menopausal status, and subtype), these signatures but not TILs were significantly associated with pCR (eTable 5 in the Supplement). Most of these signatures were associated with B cells, plasma cells, and immunoglobulins (eFigure 7 in the Supplement).

### Association of TILs and iGESs With EFS in the CALGB 40601 Trial

In the CALGB 40601 trial, 37 iGESs, but not TILs, were significantly associated with EFS in Cox regression models adjusted by treatment group (eTable 6 in the Supplement). As with pCR, the top-performing immune signatures were also associated with B cells.

Finally, we wanted to test whether the combination of iGESs and TILs was more prognostic than each alone by comparing multiple multivariable Cox regression models. To test this, we selected 6 immune signatures that outperformed TILs for pCR and were also prognostic in the CALGB 40601 trial: 3 IgG signatures, 2 B-cell signatures, and 1 plasma cell signature. The results for 1 of the IgG signatures are shown in Table 2. We first built a base model that included known prognostic variables: pCR status, treatment group (weekly paclitaxel plus trastuzumab plus lapatinib, weekly paclitaxel plus lapatinib, or weekly paclitaxel plus trastuzumab, where the latter is the reference group), hormone receptor status, clinical stage, and PAM50 intrinsic tumor subtype (HER2-enriched vs other subtypes) (model 1). In this model, treatment group, clinical stage, pCR status, and intrinsic subtype were significantly associated with EFS. Adding TILs (model 2) did not provide additional prognostic information (LRT *P* = .12). We then built a model adding our previously published IgG signature to model 1; in this model (model 3), treatment group, clinical stage, pCR status, intrinsic subtype, and the IgG signature were all significantly associated with EFS (IgG-adjusted hazard ratio, 0.63; 95% CI, 0.45-0.87; *P* = .006). Also, model 3 was significantly better than model 1 for EFS (LRT *P* = .005). The last model (model 4) included both TILs and the IgG signature. In this model, the IgG signature, but not TILs, was significantly associated with EFS (IgG-adjusted hazard ratio, 0.63; 95% CI, 0.42-0.93; *P* = .02; TIL-adjusted hazard ratio, 1.00; 95% CI, 0.98-1.02; *P* = .99) (Table 2). Model 4, including both TILs and the IgG signature, was significantly better than the model including only TILs (model 4 vs model 2; LRT *P* = .02), but it was not significantly better than model 3, which included only the IgG signature (model 4 vs model 3; LRT *P* = .99). Similar results were observed with the other 5 iGESs (eTable 7 in the Supplement) when TILs was used as a discrete variable with a cutoff of 40% (eTable 8 in the Supplement) and when a landmark analysis was performed (eTable 9 in the Supplement). When different multivariate models including clinical parameters and 1 iGES were compared, the immunoglobulin–The Cancer Genome Atlas signature model performed slightly better than the rest (eTable 10 and eTable 11 in the Supplement).

**Table 2.** Association of TILs and Immune-Related Gene Expression Signatures With EFS in the Cancer and Leukemia Group B 40601 Trial

Model, formula, and features	HR (95% CI)	*P* value	AIC	LRT *P* value^a^
Model 1: EFS ≈ treatment + HR + stage + pCRB + subtype				
THL vs TH	0.34 (0.14-0.78)	.01	395.68	1 [Reference]
TL vs TH	1.27 (0.62-2.60)	.51		
HR (pos vs neg)	1.86 (0.94-3.69)	.08		
Stage (III vs II)	2.03 (1.07-3.87)	.03		
pCR (pCR vs RD)	0.22 (0.10-0.48)	<.001		
HER2-enriched vs other	4.20 (1.97-8.96)	<.001		
Model 2: EFS ≈ treatment + HR + stage + pCRB + subtype + TILs				
THL vs TH	0.31 (0.13-0.73)	.007	395.31	Model 2 vs model 1 LRT: *P* = .12
TL vs TH	1.30 (0.63-2.67)	.48		
HR (pos vs neg)	1.85 (0.93-3.69)	.08		
Stage (III vs II)	1.95 (1.02-3.71)	.04		
pCR (pCR vs RD)	0.24 (0.11-0.54)	<.001		
HER2-enriched vs not	4.53 (2.11-9.73)	<.001		
TILs (continuous)	0.99 (0.97-1.00)	.14		
Model 3: EFS ≈ treatment + HR + stage + pCRB + subtype + signature				
THL vs TH	0.31 (0.13-0.71)	.006	389.96	Model 3 vs model 1 LRT: *P* = .005
TL vs TH	1.45 (0.70-3.02)	.32		
HR (pos vs neg)	1.43 (0.71-2.90)	.32		
Stage (III vs II)	2.01 (1.06-3.83)	.03		
pCR (pCR vs RD)	0.30 (0.13-0.66)	.003		
HER2-enriched vs not	4.28 (2.02-9.08)	<.001		
Signature (continuous)	0.63 (0.45-0.87)	.006		
Model 4: EFS ≈ treatment + HR + stage + pCRB + subtype + signature + TILs				
THL vs TH	0.31 (0.13-0.72)	.006	391.96	Model 4 vs model 2 LRT: *P* = .02
TL vs TH	1.45 (0.69-3.02)	.32		
HR (pos vs neg)	1.43 (0.70-2.92)	.32		
Stage (III vs II)	2.01 (1.05-3.84)	.03		
pCR (pCR vs RD)	0.30 (0.13-0.66)	.003		Model 4 vs model 3 LRT: *P* = .99
HER2-enriched vs not	4.28 (2.01-9.12)	<.001		
TILs (continuous)	1.00 (0.98-1.02)	.99		
Signature (continuous)	0.63 (0.42-0.93)	.02		

Abbreviations: AIC, Akaike information criterion; EFS, event-free survival; HR, hormone receptor; LRT, likelihood ratio test; neg, negative; pCR, pathologic complete response; pCRB, in-breast pCR; pos, positive; RD, residual disease; TH, weekly paclitaxel plus trastuzumab; THL, TH plus lapatinib; TIL, tumor infiltrating lymphocyte; TL, weekly paclitaxel plus lapatinib.

^a^ This was a comparative analysis of nested multivariable Cox regression models using an LRT. The signature identification from the IgG signature is IGG.Cluster Fan BMCMedGenomics.2011 PMID.21214954.

## Discussion

In the CALGB 40601 and PAMELA trials, both the proportion of TILs and the multiple iGESs were significantly associated with pCR. Moreover, in the CALGB 40601 trial, several immune signatures were also associated with EFS in univariable and multivariable Cox analyses that included clinical factors and intrinsic subtype, whereas TILs were not significantly associated with outcome. B-cell signatures outperformed TILs and T-cell signatures for pCR and EFS.^40^ Combining TILs and iGESs did not provide additional prognostic information. These results are especially relevant in early-stage ERBB2/HER2-positive breast cancer, in which multiple trials focus on developing prognostic tools combining tumor and immune cell biomarkers to guide treatment escalation and de-escalation.^25,41,42,43^

The proportion of TILs as a continuous variable has proven to be an independent prognostic biomarker in early-stage ERBB2/HER2-positive breast cancer.^13,14,15,16,17,18^ Thus, the evaluation of TILs has been proposed as a readily available tool to identify different prognostic groups in this setting. Despite the standardization of TIL scoring by the International TILs Working Group, this biomarker still has low reproducibility rates across pathologists, with κ values in the 0.4 to 0.6 range^44,45^ even after efforts to define the optimal TIL cutoff.^46^ However, these are ongoing harmonization efforts, and TILs have value, particularly in resource-constrained settings.

Not surprisingly, quantitative gene expression of immune-related genes and signatures is strongly associated with the infiltration of TILs.^47,48^ Immune genes and signatures have also proven to have prognostic and predictive value in patients with early-stage ERBB2/HER2-positive breast cancer, and the IgG signature is included in the HER2DX genomic test.^21,22,23,25^ To our knowledge, this is the first study demonstrating that when both TILs and immune gene expression data are available, iGESs, particularly B-cell signatures, provide more prognostic information in ERBB2/HER2-positive breast cancer without the additional value of adding TILs. Similar results suggest the potential superiority of iGESs over TILs in triple-negative breast cancer treated with chemotherapy alone in the CALGB 40603 trial,^48^ in which B-cell features, including IgG expression, were found to be the most prognostically valuable metric.

### Limitations

Our study has limitations. First, a substantial proportion of patients included in the CALGB 40601 and PAMELA trials received trastuzumab combined with lapatinib, a dual treatment used in the metastatic setting but not approved for early-stage ERBB2/HER2-positive breast cancer. Moreover, the PAMELA trial differed from the CALGB 40601 trial in that patients received only anti-ERBB2/HER2 therapy before surgery, without chemotherapy. Second, although EFS was a key secondary end point of the CALGB 40601 trial, the trial was not powered for long-term outcomes, so EFS prediction modeling should be interpreted with caution. Third, even when scored as a continuous variable, the proportion of TILs (ie, 0% to 100%) follows a semiquantitative pattern, with increments of 5% to 10%, and thus is not a true continuous variable like iGESs. However, when TILs were divided into high vs low levels based on multiple prespecified cutoffs, their ability to predict response and survival was lower than that of multiple B-cell–related signatures. Finally, we performed numerous statistical predictions simultaneously by building 1 model for each immune biomarker to predict pCR and EFS. To control type I error, we adjusted the models’ *P* values for multiple testing.

## Conclusions

To conclude, accumulating evidence supports the validity of using evidence of immune activation, which can be measured with TILs or immune-related gene expression biomarkers, to stratify patients with early-stage ERBB2/HER2-positive breast cancer into different prognostic groups. This study supports that measurement of immune activation, either by TIL measurement or by immune-related gene expression profiling, is predictive of treatment response and that immune-related gene expression is prognostic. In the presence of both immune biomarker types, iGESs, especially B-cell–related signatures, outperform TILs for both pCR and prognosis, and the combination of both biomarkers does not yield improved prognostic value. These results highlight the essential role of B cells in antitumor immunity and suggest that B-cell immune-related gene expression provides valuable prognostic information for treatment escalation and de-escalation in patients with early-stage ERBB2/HER2-positive breast cancer.

## Notes

Supplement. **eFigure 1.** CONSORT Diagram **eFigure 2.** Distribution of Tumor-Infiltrating Lymphocytes Infiltration by Clinical Trial **eFigure 3.** Comparison of Stromal Tumor-Infiltrating Lymphocytes (TILs) Levels by Hormone Receptor Status and Intrinsic Subtype in CALGB 40601 and PAMELA **eFigure 4.** Correlation Between Tumor-Infiltrating Lymphocytes (TILs) Levels and Immune Gene Expression Signatures (iGES) **eFigure 5.** Association Between the Spearman Correlation Coefficients of Tumor-Infiltrating Lymphocytes (TILs) and Immune Gene Expression Signatures (iGES) in CALGB 40601 and PAMELA **eFigure 6.** Rates of Pathologic Complete Response (pCR) According to Immune Gene Expression Signature (iGES) Levels **eFigure 7.** Accuracy Metrics of the Univariable Immune Biomarker Models for Pathologic Complete Response (pCR) Prediction **eTable 1.** List of Immune Gene Expression Signatures, Signature Class, PMID, and Genes Within a Signature **eTable 2.** Comparison of Baseline Characteristics of the Patients From the CALGB 40601 Event-Free Survival (EFS) and Landmark Subpopulations **eTable 3.** Correlation of Tumor-Infiltrating Lymphocytes (TILs) and Immune Gene Expression Signatures (iGES) in CALGB 40601 and PAMELA **eTable 4.** Association of Tumor-Infiltrating Lymphocytes (TILs) and Immune Gene Expression Signatures (iGES) With Pathologic Complete Response (pCR) in the Combined CALGB 40601 and PAMELA Data Set **eTable 5.** Association of Tumor-Infiltrating Lymphocytes (TILs) and Immune Gene Expression Signatures (iGES) With Pathologic Complete Response (pCR) in the Presence of Clinical Parameters Using the Combined CALGB 40601 and PAMELA Cohort **eTable 6.** Accuracy Metrics (Area Under the Curve From the Receiver Operating Characteristic Curves [AUC ROC]) of the Univariable Immune-Biomarker Models to Predict Pathologic Complete Response (pCR) **eTable 7.** Association of Tumor-Infiltrating Lymphocytes (TILs) and Immune Gene Expression Signatures (iGES) With Event-Free Survival (EFS) in CALGB 40601 **eTable 8.** Association of Tumor-Infiltrating Lymphocytes (TILs) as a Continuous Variable and Immune Gene Expression Signatures (iGES) With Event-Free Survival (EFS) in CALGB 40601 **eTable 9.** Association of Tumor-Infiltrating Lymphocytes (TILs) Using a Cutoff of 40% and Immune Gene Expression Signatures (iGES) With Event-Free Survival (EFS) in CALGB 40601 **eTable 10.** Landmark Analysis Week 30 **eTable 11.** Summary Table of Akaike Information Criteria (AIC) and C-Index From Multivariable Cox Models Including Immune Gene Expression Signatures (iGES)
eReferences
