# Supplementary material for: Prognostic and Predictive Value of Immune-Related Gene Expression Signatures vs Tumor-Infiltrating Lymphocytes in Early-Stage ERBB2/HER2-Positive Breast Cancer: A Correlative Analysis of the CALGB 40601 and PAMELA Trials
Source: JAMA Oncol. 2023 Jan 5;9(4):490–9. doi: 10.1001/jamaoncol.2022.6288 (PMC9857319; doi:10.1001/jamaoncol.2022.6288)
Supplement: Supplement. — eFigure 1. CONSORT Diagram eFigure 2. Distribution of Tumor-Infiltrating Lymphocytes Infiltration by Clinical Trial eFigure 3. Comparison of Stromal Tumor-Infiltrating Lymphocytes (TILs) Levels by Hormone Receptor Status and Intrinsic Subtype in CALGB 40601 and PAMELA eFigure 4. Correlation Between Tumor-Infiltrating Lymphocytes (TILs) Levels and Immune Gene Expression Signatures (iGES) eFigure 5. Association Between the Spearman Correlation Coefficients of Tumor-Infiltrating Lymphocytes (TILs) and Immune Gene Expression Signatures (iGES) in CALGB 40601 and PAMELA eFigure 6. Rates of Pathologic Complete Response (pCR) According to Immune Gene Expression Signature (iGES) Levels eFigure 7. Accuracy Metrics of the Univariable Immune Biomarker Models for Pathologic Complete Response (pCR) Prediction eTable 1. List of Immune Gene Expression Signatures, Signature Class, PMID, and Genes Within a Signature eTable 2. Comparison of Baseline Characteristics of the Patients From the CALGB 40601 Event-Free Survival (EFS) and Landmark Subpopulations eTable 3. Correlation of Tumor-Infiltrating Lymphocytes (TILs) and Immune Gene Expression Signatures (iGES) in CALGB 40601 and PAMELA eTable 4. Association of Tumor-Infiltrating Lymphocytes (TILs) and Immune Gene Expression Signatures (iGES) With Pathologic Complete Response (pCR) in the Combined CALGB 40601 and PAMELA Data Set eTable 5. Association of Tumor-Infiltrating Lymphocytes (TILs) and Immune Gene Expression Signatures (iGES) With Pathologic Complete Response (pCR) in the Presence of Clinical Parameters Using the Combined CALGB 40601 and PAMELA Cohort eTable 6. Accuracy Metrics (Area Under the Curve From the Receiver Operating Characteristic Curves [AUC ROC]) of the Univariable Immune-Biomarker Models to Predict Pathologic Complete Response (pCR) eTable 7. Association of Tumor-Infiltrating Lymphocytes (TILs) and Immune Gene Expression Signatures (iGES) With Event-Free Survival (EFS) in CALGB 40601 eTable 8. Association of Tumo [file jamaoncol-e226288-s001.pdf]

Supplementary Online Content

Fernandez-Martinez A, Pascual T, Singh B, et al. Prognostic and predictive value of immune-related gene expression signatures vs tumor-infiltrating lymphocytes in early-stage ERBB2/HER2-positive breast cancer: a correlative analysis of the CALGB 40601 and PAMELA trials. *JAMA Oncol*. Published online January 5, 2023. doi:10.1001/jamaoncol.2022.6288

eFigure 1. CONSORT Diagram
eFigure 2. Distribution of Tumor-Infiltrating Lymphocytes Infiltration by Clinical Trial
eFigure 3. Comparison of Stromal Tumor-Infiltrating Lymphocytes (TILs) Levels by Hormone Receptor Status and Intrinsic Subtype in CALGB 40601 and PAMELA
eFigure 4. Correlation Between Tumor-Infiltrating Lymphocytes (TILs) Levels and Immune Gene Expression Signatures (iGES)
eFigure 5. Association Between the Spearman Correlation Coefficients of Tumor-Infiltrating Lymphocytes (TILs) and Immune Gene Expression Signatures (iGES) in CALGB 40601 and PAMELA
eFigure 6. Rates of Pathologic Complete Response (pCR) According to Immune Gene Expression Signature (iGES) Levels
eFigure 7. Accuracy Metrics of the Univariable Immune Biomarker Models for Pathologic Complete Response (pCR) Prediction
eTable 1. List of Immune Gene Expression Signatures, Signature Class, PMID, and Genes Within a Signature
eTable 2. Comparison of Baseline Characteristics of the Patients From the CALGB 40601 Event-Free Survival (EFS) and Landmark Subpopulations
eTable 3. Correlation of Tumor-Infiltrating Lymphocytes (TILs) and Immune Gene Expression Signatures (iGES) in CALGB 40601 and PAMELA
eTable 4. Association of Tumor-Infiltrating Lymphocytes (TILs) and Immune Gene Expression Signatures (iGES) With Pathologic Complete Response (pCR) in the Combined CALGB 40601 and PAMELA Data Set
eTable 5. Association of Tumor-Infiltrating Lymphocytes (TILs) and Immune Gene Expression Signatures (iGES) With Pathologic Complete Response (pCR) in the Presence of Clinical Parameters Using the Combined CALGB 40601 and PAMELA Cohort
eTable 6. Accuracy Metrics (Area Under the Curve From the Receiver Operating Characteristic Curves [AUC ROC]) of the Univariable Immune-Biomarker Models to Predict Pathologic Complete Response (pCR)
eTable 7. Association of Tumor-Infiltrating Lymphocytes (TILs) and Immune Gene Expression Signatures (iGES) With Event-Free Survival (EFS) in CALGB 40601
eTable 8. Association of Tumor-Infiltrating Lymphocytes (TILs) as a Continuous Variable and Immune Gene Expression Signatures (iGES) With Event-Free Survival (EFS) in CALGB 40601
eTable 9. Association of Tumor-Infiltrating Lymphocytes (TILs) Using a Cutoff of 40% and Immune Gene Expression Signatures (iGES) With Event-Free Survival (EFS) in CALGB 40601
eTable 10. Landmark Analysis Week 30
eTable 11. Summary Table of Akaike Information Criteria (AIC) and C-Index From Multivariable Cox Models Including Immune Gene Expression Signatures (iGES)
eReferences

This supplementary material has been provided by the authors to give readers additional information about their work.

© 2023 Fernandez-Martinez A et al. *JAMA Oncology.*

eFigure 1. CONSORT Diagram

TILs: Tumor-Infiltrating Lymphocytes; Rx: treatment; QC: quality control; w: weeks.

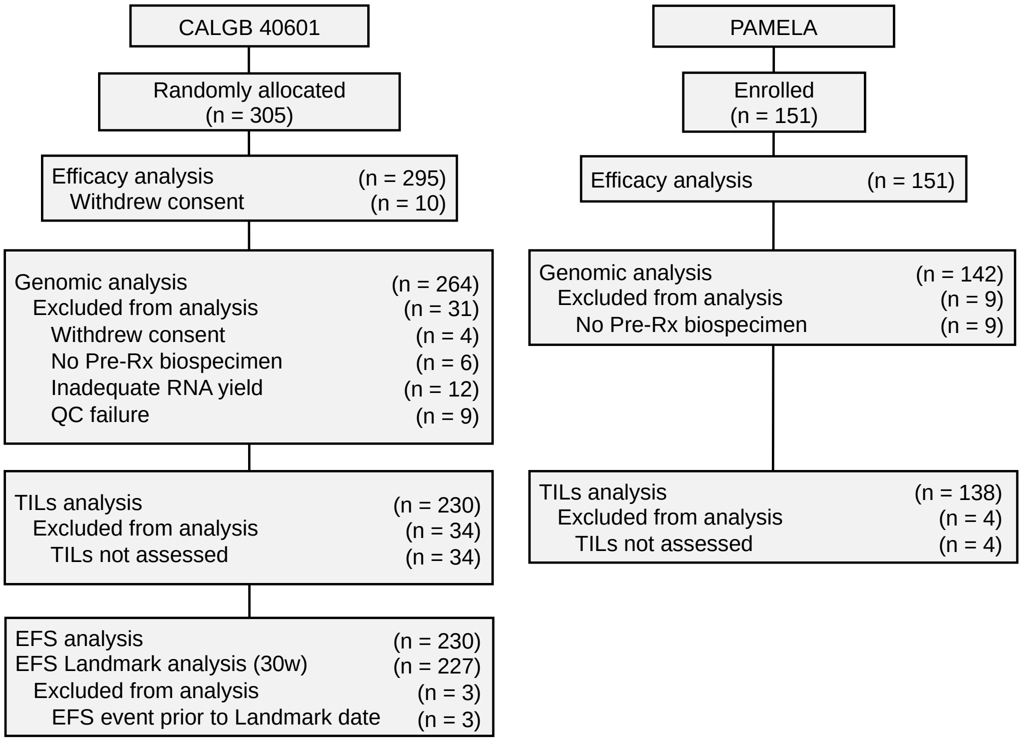

© 2023 Fernandez-Martinez A et al. *JAMA Oncology.*

eFigure 2. Distribution of Tumor-Infiltrating Lymphocytes Infiltration by Clinical Trial

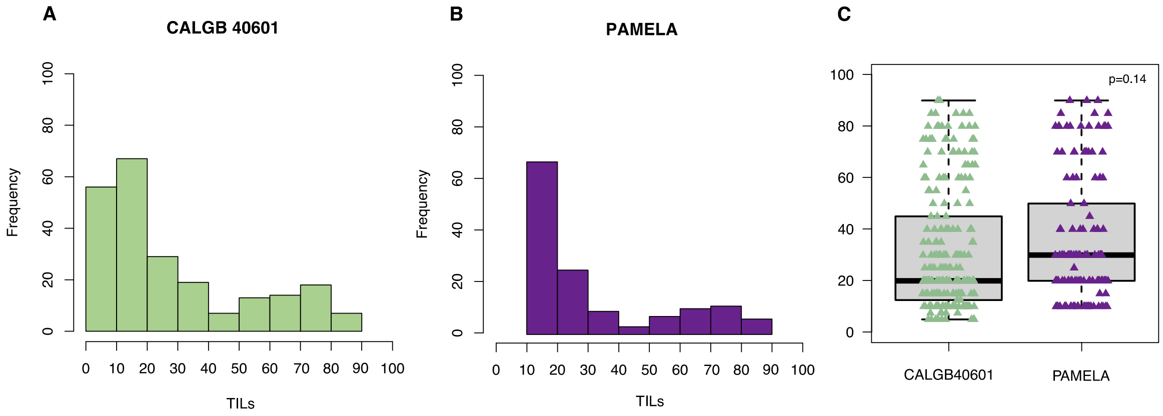

A) Histogram of Tumor-Infiltrating Lymphocytes infiltration levels in CALGB 40601. B) Histogram of Tumor-Infiltrating Lymphocytes infiltration levels in PAMELA. C) Comparison of Tumor-Infiltrating Lymphocytes infiltration levels between both clinical trials. Statistical differences were assessed using an Kruskal Wallis test (P-value at the top of the figure). The horizontal line from the boxplots represents the median of the distribution. TILs: Tumor-Infiltrating Lymphocytes.

© 2023 Fernandez-Martinez A et al. *JAMA Oncology.*

eFigure 3. Comparison of Stromal Tumor-Infiltrating Lymphocytes (TILs) Levels by Hormone Receptor Status and Intrinsic Subtype in CALGB 40601 and PAMELA

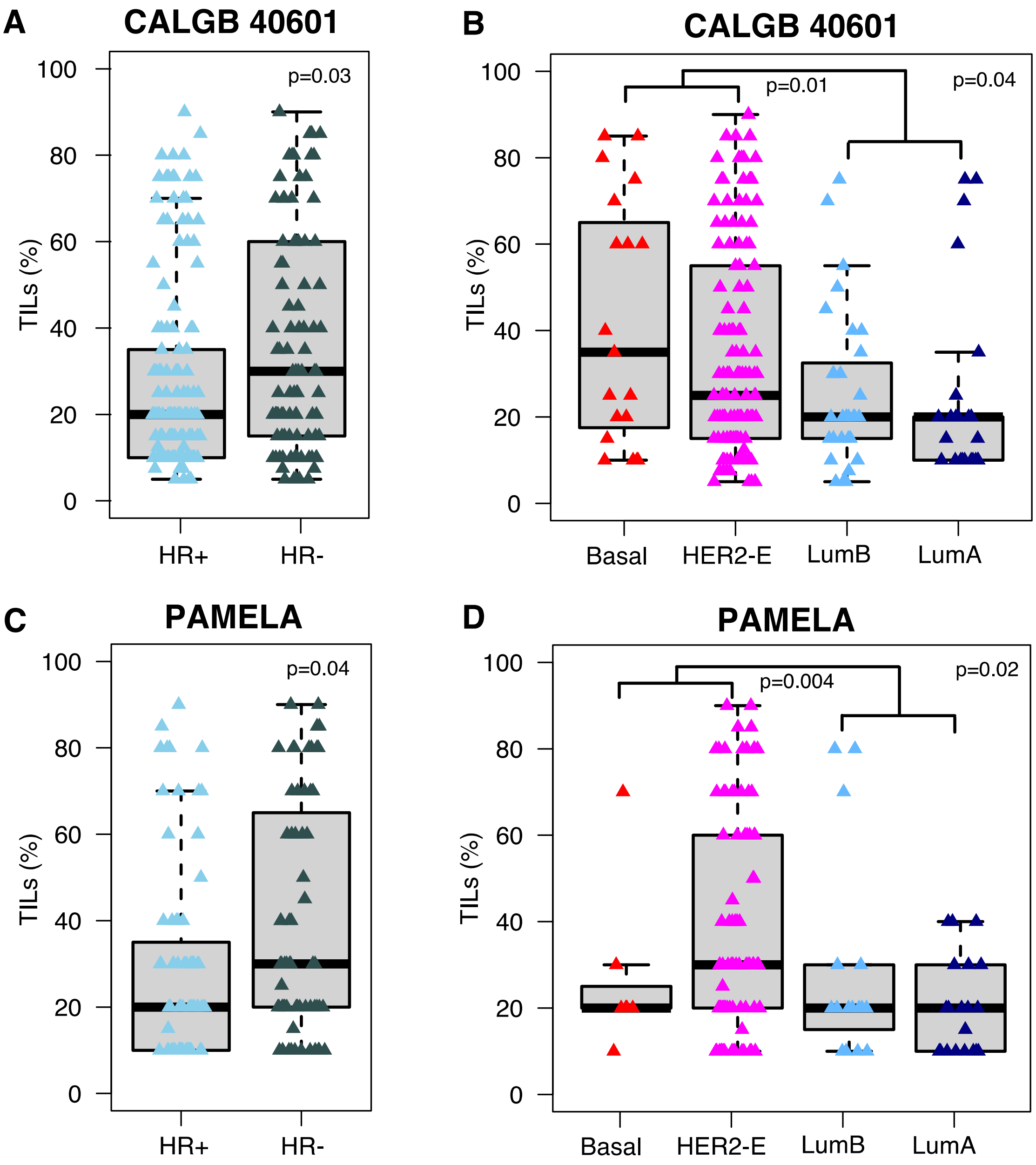

Comparison of stromal Tumor-Infiltrating Lymphocytes (TILs) levels by hormone receptor status and intrinsic subtype in CALGB 40601 (A, B) and PAMELA (C, D) trials. Statistical differences were assessed using an Kruskal Wallis test (P-value at the top of the figure). The horizontal line from the boxplots represents the median of the distribution.
TILs: Tumor-Infiltrating Lymphocytes; HR+: hormone receptor positive; HR-: hormone receptor negative; Basal: basal-like; HER2-E: HER2-Enriched; LumB: luminal B; LumA: luminal A.

© 2023 Fernandez-Martinez A et al. *JAMA Oncology.*

eFigure 4. Correlation Between Tumor-Infiltrating Lymphocytes (TILs) Levels and Immune Gene Expression Signatures (iGES)

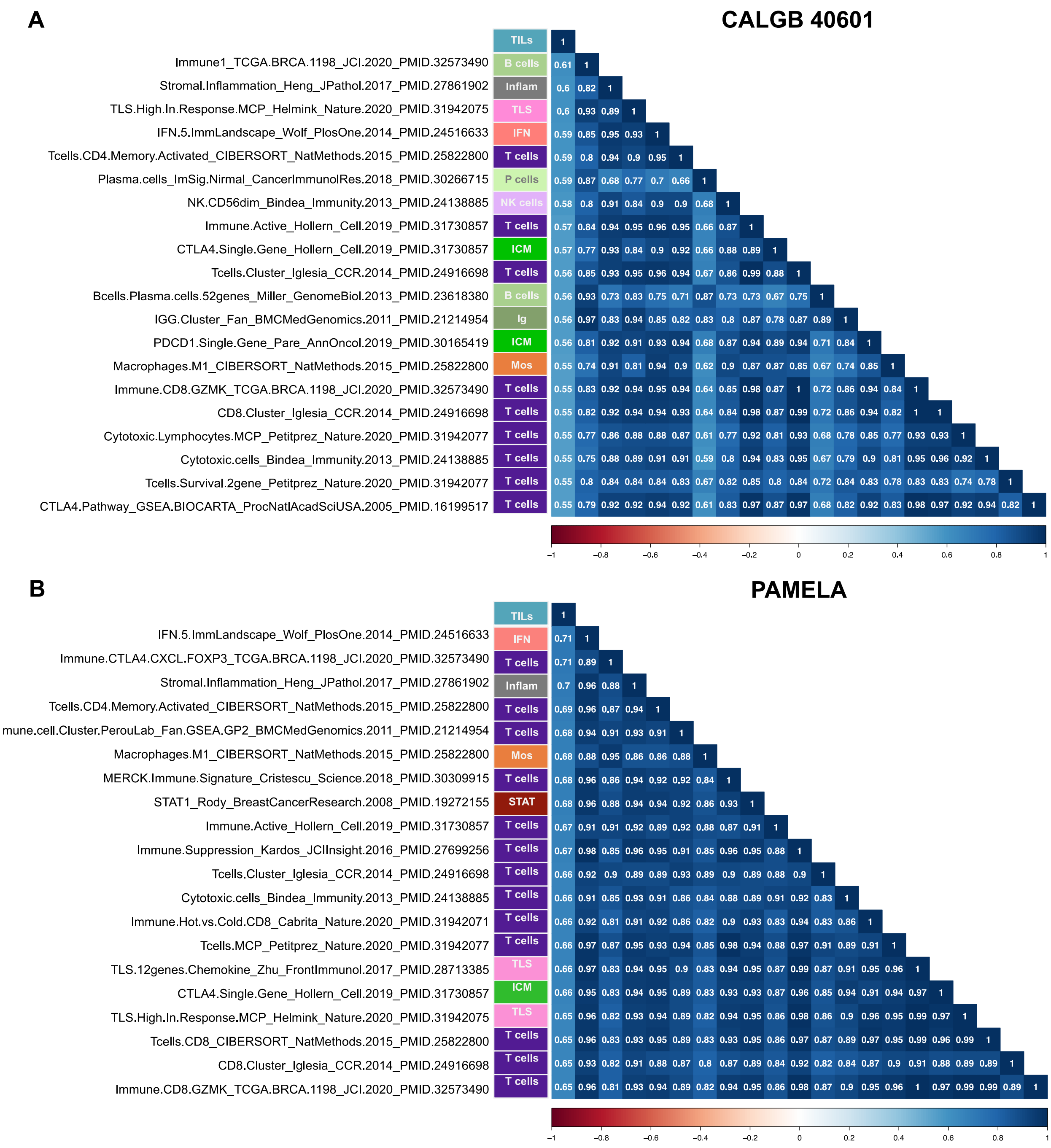

Spearman's Correlations between Tumor-Infiltrating Lymphocytes (TILs) levels and immune gene expression signatures. The 20 signatures with a p-value of 0.05 and the highest correlation coefficients are shown for A) CALGB 40601 and B) PAMELA.
TILs: Tumor-Infiltrating Lymphocytes; Inflam: inflammation; TLS: tertiary lymphoid structures; IFN: interferon; P: plasma; ICM: immune checkpoint molecules; Mos: macrophages; NK: natural killer; Ig: immunoglobulin; STAT: signal transducer and activator of transcription.

© 2023 Fernandez-Martinez A et al. *JAMA Oncology.*

eFigure 5. Association Between the Spearman Correlation Coefficients of Tumor-Infiltrating Lymphocytes (TILs) and Immune Gene Expression Signatures (iGES) in CALGB 40601 and PAMELA.

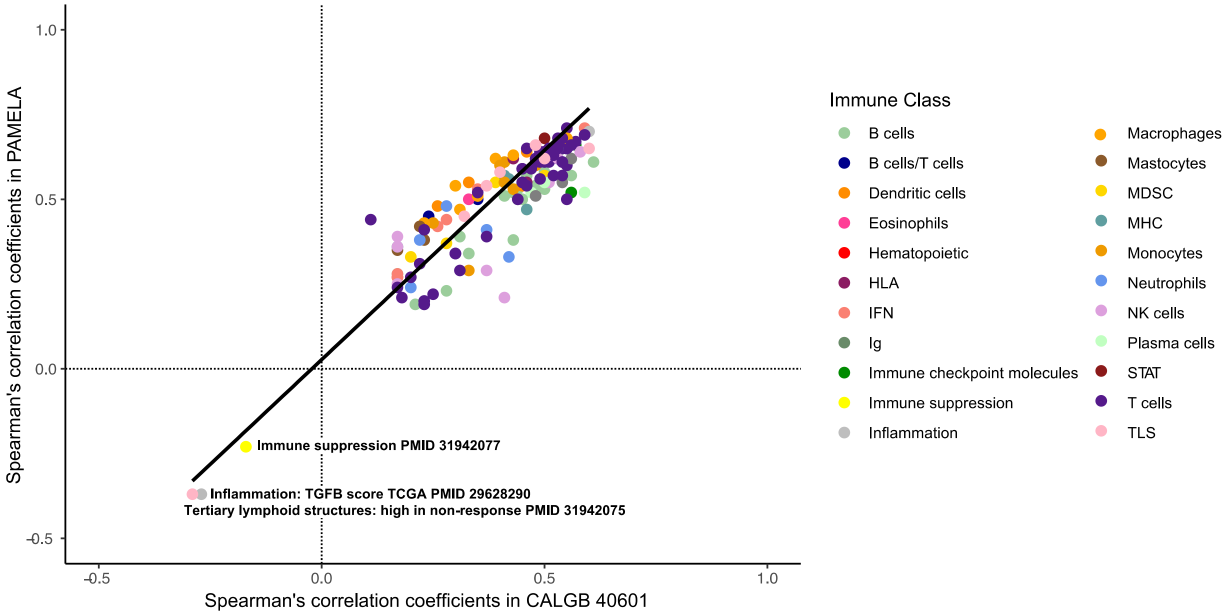

Only the signatures significantly correlated with TILs in both studies (166/202, 81.2%) are represented (adjusted p-value < 0.05).
HLA: human leukocyte antigens; IFN: interferon; Ig: Immunoglobulin; MDSC: myeloid-derived-suppressor cells; MHC: major histocompatibility complex; NK: natural killer; STAT: signal transducer and activator of transcription; TLS: tertiary lymphoid structures.

© 2023 Fernandez-Martinez A et al. *JAMA Oncology.*

eFigure 6. Rates of Pathologic Complete Response (pCR) According to Immune Gene Expression Signature (iGES) Levels

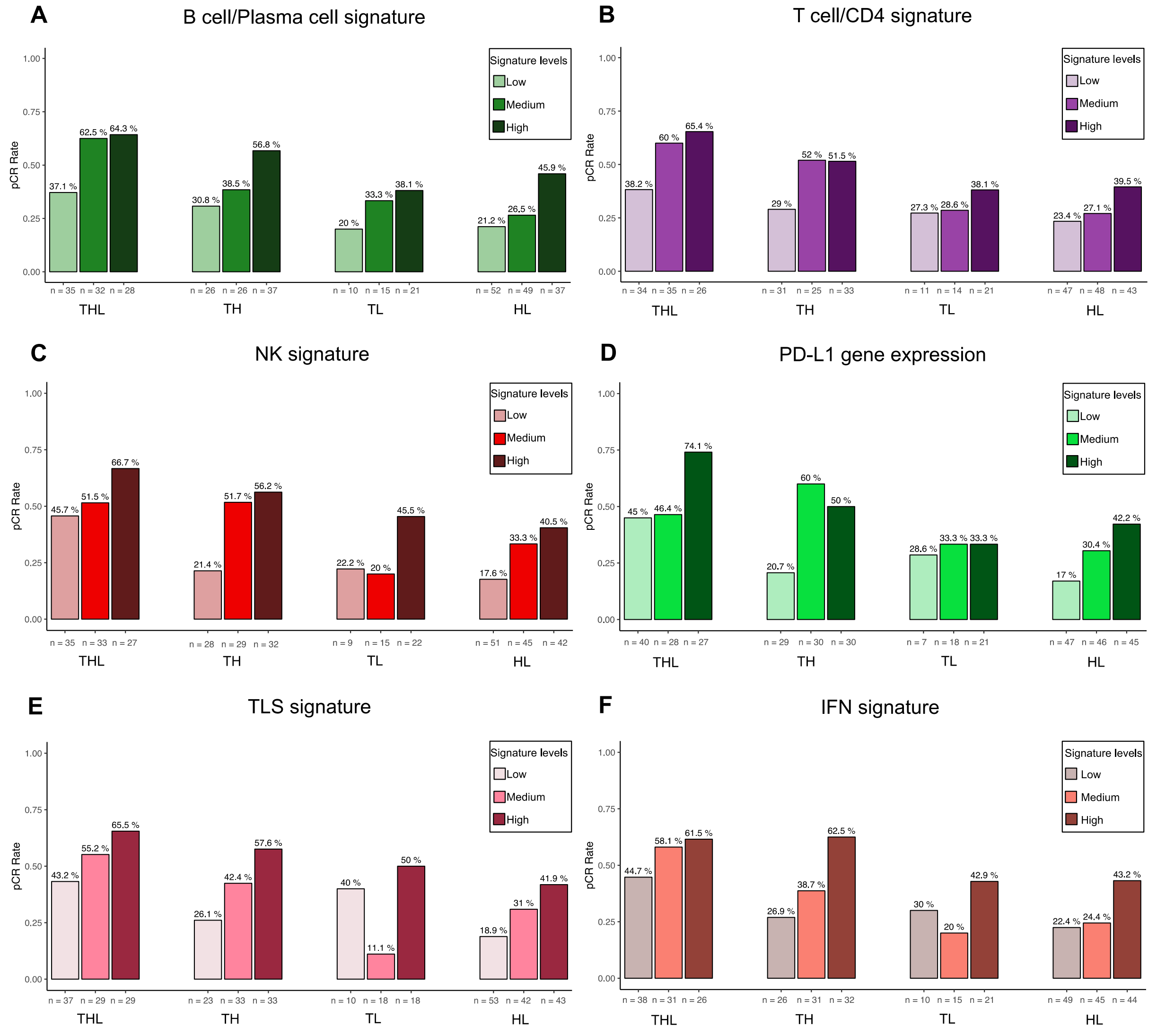

iGES have been divided by tertiles to illustrate their association with pCR rates.
pCR: pathologic complete response; T: weekly paclitaxel; H: trastuzumab; L: lapatinib; NK: natural killer; PD-L1: programmed death-1 ligand 1; TLS: tertiary lymphoid structures; IFN: interferon.

© 2023 Fernandez-Martinez A et al. *JAMA Oncology.*

eFigure 7. Accuracy Metrics of the Univariable Immune Biomarker Models for Pathologic Complete Response (pCR) Prediction

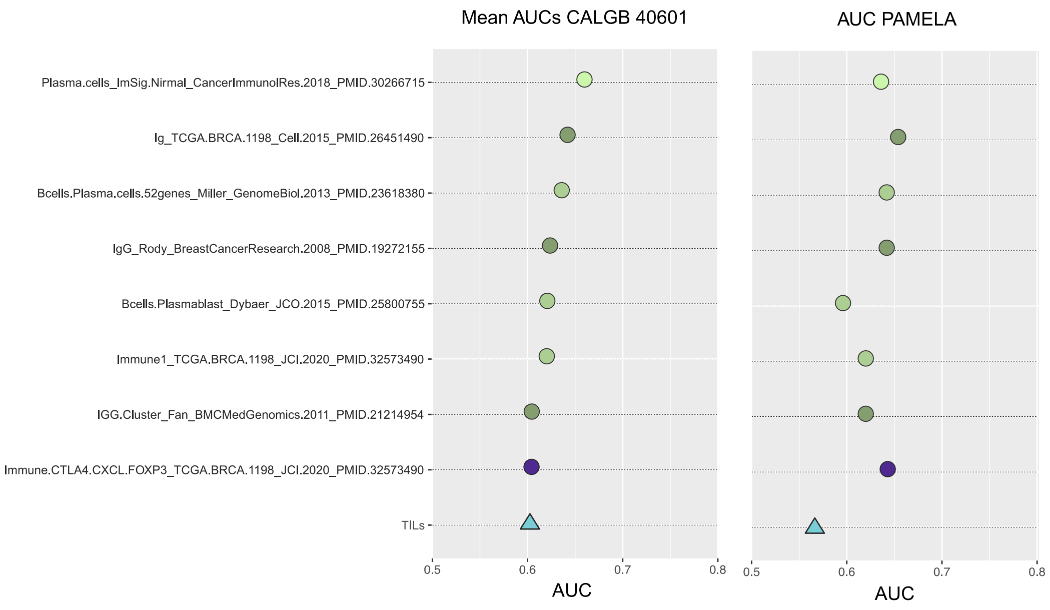

Using CALGB 40601 as training set, the average AUC of the different models was calculated across all the resamples using 10-fold cross validation. A second AUC for the different biomarkers was calculated using PAMELA as an external validation set. The gene expression signatures are represented as circles compared to TILs as continuous variable, that is represented as a triangle. The circle colors represent the immune signature class as shown in eTable 1.
TILs: Tumor-Infiltrating Lymphocytes; AUC: Area under the receiver operating characteristic curve.

© 2023 Fernandez-Martinez A et al. *JAMA Oncology.*

eTable 1. List of Immune Gene Expression Signatures, Signature Class, PMID, and Genes Within a Signature

Signature ID paper	Immune class	Signature class	PMID	Genes
**Bcells.Cluster Iglesia CCR.2014 PMID.24916698**	B cells	Signature	PMID.24916698	3512; 9834; 608; 83416; 5450; 973; 96610; 8755; 389643; 150365; 974; 5079; 2208; 79368; 643; 930; 84824; 640; 115350; 931; 1269; 55024; 23495
**Bcells.IL10.Minus Lin JImmunol.2014 PMID.25080484**	B cells	Signature	PMID.25080484	6504; 3586; 3569; 4049; 374; 50832; 1259; 5167; 2745; 54512; 92935; 6723; 760; 6507; 55176; 1960; 3280; 3662; 9021; 10406; 79005; 10794; 6503; 51559; 11147; 55784; 92002
**Bcells.IL10.Plus Lin JImmunol.2014 PMID.25080484**	B cells	Signature	PMID.25080484	225; 481; 822; 901; 946; 1491; 1545; 1831; 2149; 2170; 2213; 2316; 2838; 3087; 3512; 3552; 3587; 3597; 3663; 3687; 3695; 3782; 3937; 3956; 4000; 4046; 4063; 4233; 4938; 4940; 5027; 5996; 6346; 6505; 6843; 9235; 9447; 9760; 10150; 10225; 10550; 10628; 10752; 10800; 11343; 22797; 23208; 25842; 26207; 26230; 28984; 51284; 51676; 55785; 55809; 55824; 57379; 79026; 81553; 84561; 118429; 128553; 130367; 143686; 151963; 162394; 166824; 167838; 201799; 219285; 219855; 257144; 285386; 374618; 388325; 402415
**Bcells.Centroblast Dybaer JCO.2015 PMID.25800755**	B cells	Signature	PMID.25800755	10810; 7482; 148229; 651; 23710; 4086; 54443; 259266; 55635; 7153; 100126791; 283431; 9928; 10112; 80119; 1062; 25959; 8739; 4297; 157740; 101928620; 81930; 79071; 5450; 23089; 1063; 9787; 27109; 4751; 9582; 10733; 4798; 5577; 124989; 983; 100506844; 23766

© 2023 Fernandez-Martinez A et al. *JAMA Oncology.*

**Bcells.Centrocyte Dybaer JCO.2015 PMID.25800755**	**B cells**	Signature	PMID.25800755	55448; 8622; 101929450; 639; 54820; 116931; 94121; 327657; 64805; 597; 6662; 88; 3117; 55971; 4023; 9638; 92737; 1960; 25953; 3119; 84102; 285313; 63971; 10170; 55785; 816; 285237; 5795; 1289; 53354; 23334; 54072; 55293; 147945; 3690; 100506930; 27197; 148808; 114614; 9782; 2956; 9133; 100509457; 406947; 724102
**Bcells.Memory Dybaer JCO.2015 PMID.25800755**	**B cells**	Signature	PMID.25800755	6352; 170371; 946; 1230; 100506071; 3684; 27197; 374618; 1285; 1690; 440823; 27163; 2857; 2212; 79026; 195; 83417; 339005; 10382; 285972; 8082; 3310; 440253
**Bcells.Naive Dybaer JCO.2015 PMID.25800755**	**B cells**	Signature	PMID.25800755	55211; 10252; 4345; 351; 8496; 5243; 167410; 140733; 643733; 100128252; 8531; 8115; 342926; 909; 5796; 9976; 646113; 4606; 1901; 6328; 2650; 401312; 646588; 63934; 2208
**Bcells.Memory CIBERSORT NatMethods.2015 PMID.25822800**	**B cells**	Signature	PMID.25822800	10863; 60468; 55024; 640; 4064; 930; 911; 933; 951; 969; 971; 973; 974; 1380; 643; 9214; 2213; 79368; 2444; 2841; 91316; 3087; 3112; 3495; 3507; 3514; 91353; 3394; 80183; 4050; 9450; 931; 4829; 5026; 5368; 5790; 55103; 10235; 150094; 6557; 6689; 26228; 29802; 9447; 240; 1235; 939; 9629; 160518; 9750; 2788; 3446; 3574; 4153; 440348; 27240; 11262; 80008; 23495; 608; 9618; 27033
**Bcells.Naive CIBERSORT NatMethods.2015 PMID.25822800**	**B cells**	Signature	PMID.25822800	5244; 10863; 60468; 55024; 605; 79656; 640; 673; 4064; 930; 911; 933; 951; 969; 971; 973; 974; 1380; 643; 55840; 9214; 2208; 2213; 79368; 2444; 2841; 91316; 3087; 3112; 3495; 3507; 3514; 91353; 3566; 3394; 80183; 283876; 4050; 9450; 4224; 57553; 931; 55335; 4829;

© 2023 Fernandez-Martinez A et al. *JAMA Oncology.*

				5026; 9934; 5368; 5670; 5790; 55103; 10235; 6402; 150094; 6557; 6689; 26228; 8115; 54576; 29802; 57335
**Bcells Garber CellMolGastroenterol Hepatol.2017 PMID.28508029**	B cells	Signature	PMID.28 508029	10863; 240; 60468; 55024; 53335; 53335; 53335; 53335; 640; 643; 930; 933; 951; 951; 958; 971; 973; 2208; 2788; 3087; 3112; 3117; 114884; 5368; 25780; 6689; 8115; 29802; 9934; 55103; 55103; 55103; 6039; 6328; 608
**Bcells.Extended Garber CellMolGastroenterol Hepatol.2017 PMID.28508029**	B cells	Signature	PMID.28 508029	3899; 65069; 10409; 695; 66033; 201895; 934; 100133941; 972; 972; 973; 26047; 51523; 378885; 9214; 84824; 23062; 3119; 3126; 23231; 4067; 4082; 55846; 256236; 5336; 5777; 10509; 51092; 6643
**Bcells Bindea Immunity.2013 PMID.24138885**	B cells	Signature	PMID.24 138885	608; 640; 930; 931; 971; 1380; 1690; 1838; 2731; 2788; 3112; 3117; 3493; 3500; 3507; 3514; 3535; 4208; 5244; 5368; 6328; 6565; 6689; 8115; 9834; 10803; 29064; 29760; 53335; 55278; 57553; 60468; 79368; 114884
**Bcells.ImmuneProfil es.Mouse.Human Shay PNAS.2013 PMID.23382184**	B cells	Signature	PMID.23 382184	19; 330; 489; 605; 640; 643; 695; 930; 931; 933; 951; 958; 971; 972; 973; 974; 1185; 1235; 1380; 1387; 1456; 1657; 1960; 2181; 2185; 2208; 2841; 2872; 2889; 3280; 3399; 3592; 3662; 3708; 3915; 4033; 4064; 4208; 4209; 4214; 4641; 5079; 5106; 5142; 5287; 5290; 5336; 5451; 5452; 5530; 5579; 5777; 5925; 5966; 5993; 6480; 6565; 6598; 6643; 6689; 6721; 6850; 6925; 6929; 6949; 7187; 7430; 7799; 7942; 8202; 8527; 8564; 8930; 8942; 9015; 9026; 9135; 9308; 9450; 9451; 9467; 9640; 9657; 9665; 9681; 9711; 9734; 9779; 9788; 9873; 9896; 9922; 10193; 10447; 10564; 11108; 11142; 22837; 22876; 22898;

© 2023 Fernandez-Martinez A et al. *JAMA Oncology.*

				22906; 23035; 23062; 23075; 23216; 23325; 23411; 23495; 23515; 23596; 24149; 25780; 26040; 26051; 26228; 27314; 27334; 29116; 29760; 29802; 50618; 51284; 51363; 51585; 53335; 54468; 54920; 55016; 55024; 55114; 55149; 55278; 55500; 55565; 55589; 55702; 55788; 57102; 57157; 57396; 58513; 59339; 59342; 60468; 60485; 64376; 64783; 64853; 65117; 65986; 79161; 79365; 79591; 79600; 79622; 79651; 79874; 80021; 80183; 80221; 80237; 83478; 84159; 160518; 200576; 201895; 221037; 221749; 283232; 5450; 9712; 101928620; 100507213
**Bcells.Activated Charoentong CellRep.2017 PMID.28052254**	B cells	Signature	PMID.28052254	79368; 3507; 931; 10863; 80709; 9938; 60468; 640; 6366; 4064; 930; 939; 952; 974; 388512; 283420; 160365; 1380; 2788; 3112; 3514; 57553; 5368; 6689; 8115; 608
**Bcells.Immature Charoentong CellRep.2017 PMID.28052254**	B cells	Signature	PMID.28052254	115350; 26228; 933; 1536; 199786; 115352; 83416; 84824; 9734; 3117; 84329; 9711; 653361; 654816; 27334; 6672; 117289; 10628; 54877
**Bcells.Memory Charoentong CellRep.2017 PMID.28052254**	B cells	Signature	PMID.28052254	4609; 57379; 890; 1033; 1184; 5167; 2205; 83417; 860; 6653; 6660; 6776; 6777; 54106
**Bcells ImSig.Nirmal CancerImmunolRes. 2018 PMID.30266715**	B cells	Signature	PMID.30266715	283663; 100507616; 29802; 640; 79368; 8115; 5079; 115350; 4063; 971; 83416; 115352; 8698; 5452; 931; 79856; 933; 199786; 3112; 81793; 1380; 26228; 3899; 4064; 5026; 151888; 951; 1235; 930; 28387; 5368; 1879; 974; 84824; 55024; 973
**Bcells.Plasma.cells.52genes Miller GenomeBiol.2013 PMID.23618380**	B cells	Signature	PMID.23618380	973; 26586; 9917; 3662; 54900; 4917; 5450; 608; 3512; 91316; 100423062; 723778; 3500; 28461;

© 2023 Fernandez-Martinez A et al. *JAMA Oncology.*

				28937; 91353; 28862; 28939; 3507; 3525; 28797; 106481689; 28445; 3531; 28444; 28391; 28442; 3493; 28893; 28803; 3494; 28902; 28883; 28894; 28900; 3505; 106480274; 28921; 28452; 28930; 28815; 28931; 28908; 28458; 28912; 28793; 3538; 644731; 3532; 3537; 28392; 3539
**Immune.CD19 TCGA.BRCA.1198 JCI.2020 PMID.32573490**	**B cells**	Signature	PMID.32573490	8115; 79368; 930; 640; 1269; 931; 84824; 5079
**Immune1 TCGA.BRCA.1198 JCI.2020 PMID.32573490**	**B cells**	Signature	PMID.32573490	952; 9834; 973; 83416; 5450; 96610; 608; 8755; 3662; 54900; 389643
**Bcells.Tcells.Cooperation Hollern Cell.2019 PMID.31730857**	**B cells/T cells**	Signature	PMID.31730857	115350; 640; 3502; 3502; 28776; 3500; 3502; 3514; 3512; 973; 100423062; 100423062; 3495; 930; 931; 5079; 3581; 6363; 51617; 2864; 959; 28558; 28621; 387923; 388507
**Immune.14 Perez JCO.2015 PMID.2560586**	**B cells/T cells**	Signature	PMID.25605861	84632; 120425; 6366; 1233; 913; 959; 6387; 2534; 3112; 3487; 3394; 5729; 5734; 81793
**Tcells.Bcells.Lymphocyte.Infiltration Calabro BreastCancerResTreat.2009 PMID.18592372**	**B cells/T cells**	Signature	PMID.18592372	6352; 930; 951; 915; 916; 917; 919; 973; 974; 925; 926; 3502; 3512; 3537; 929; 3932; 4050; 931
**Immune.CD4.CD53.CD84.BTK TCGA.BRCA.1198 JCI.2020 PMID.32573490**	**B cells/T cells**	Signature	PMID.32573490	221472; 257106; 1536; 8832; 22797; 313; 3394; 1234; 2124; 695; 920; 963; 5341; 1794; 3071; 3937; 2533; 5788; 10320; 3587; 3594; 64333; 54440; 5778; 124460; 6693; 388325; 89857; 64092; 23533; 286336; 951; 199; 7940; 79626; 3059; 27128; 4542; 83706; 6688; 7454; 4689; 64098; 6404
**CD103.Negative Broz CancerCell.2014 PMID.25446897**	**Dendritic cells**	Signature	PMID.25446897	728; 10894; 8714; 4360; 6614; 23166; 713; 712; 140738; 10461; 714; 338773; 58475; 348; 8529; 54209; 51284

© 2023 Fernandez-Martinez A et al. *JAMA Oncology.*

**Dendritic.cells.Activated CIBERSORT NatMethods.2015 PMID.25822800**	Dendritic cells	Signature	PMID.25822800	51296; 1193; 6362; 54209; 6357; 6367; 7941; 6361; 910; 913; 2359; 4321; 55026; 6355; 10148; 6614; 3357; 80380; 6352; 3559; 5739; 1236; 6363; 3627; 6373; 79132; 8820; 3620; 10964; 8942; 27074; 51365; 83937; 91543; 11182; 7130; 1593; 7293; 6364; 58504; 330; 6346; 941; 942; 56548; 2117; 3593; 9175; 9242; 8013; 81796; 10402; 8792
**Dendritic.cells.Resting CIBERSORT NatMethods.2015 PMID.25822800**	Dendritic cells	Signature	PMID.25822800	51296; 1193; 968; 51313; 6362; 54209; 199; 50856; 2535; 246; 6357; 30835; 10462; 10261; 4688; 54; 79839; 6367; 7941; 56833; 945; 64581; 6039; 6335; 911; 2208; 79630; 6361; 909; 910; 913; 79154; 1959; 2205; 55640; 2359; 3117; 4321; 8496; 55026
**Dendritic.cells.Activated Bindea Immunity.2013 PMID.24138885**	Dendritic cells	Signature	PMID.24138885	3620; 4940; 6346; 10148; 27074
**Dendritic.cells Bindea Immunity.2013 PMID.24138885**	Dendritic cells	Signature	PMID.24138885	3290; 4881; 6357; 6361; 6367; 8495; 30835
**Denditic.cells.ImmuneProfiles.Mouse.Human Shay PNAS.2013 PMID.23382184**	Dendritic cells	Signature	PMID.23382184	1471; 1776; 2322; 2828; 3383; 4610; 5058; 5199; 5547; 6252; 7045; 8321; 9314; 10875; 23677; 28955; 51177; 54802; 55022; 55509; 55521; 57118; 57698; 79887; 80005; 80176
**Dendritic.cells.Activated Charoentong CellRep.2017 PMID.28052254**	Dendritic cells	Signature	PMID.28052254	822; 4843; 6503; 8926; 10011; 215; 10096; 506; 10632; 523; 598; 713; 714; 414062; 8900; 50489; 9936; 1088; 170482; 3118; 5861; 387; 8778; 89886; 51312; 60559; 7127; 8771; 8740; 54210; 340205; 285852; 10537; 8875; 23214
**Dendritic.cells.Immature. Charoentong CellRep.2017 PMID.28052254**	Dendritic cells	Signature	PMID.28052254	400; 22876; 353514; 34; 10768; 8854; 224; 223; 246; 271; 275; 471; 498; 128346; 829; 1212; 1441; 5328; 23682; 5962; 58528; 6571; 4070

© 2023 Fernandez-Martinez A et al. *JAMA Oncology.*

**Dendritic.cells.Immature. Bindea Immunity.2013 PMID.24138885**	Dendritic cells	Signature	PMID.24138885	645; 909; 910; 911; 913; 1436; 1497; 2162; 2167; 2535; 2952; 2978; 4070; 4321; 5468; 5550; 5909; 8566; 9023; 9429; 9956; 10462; 22846; 23428; 51760; 53343; 64170; 64231; 65010; 81501; 81562
**Plasmacytoid.Dendritic.cell Charoentong CellRep.2017 PMID.28052254**	Dendritic cells	Signature	PMID.28052254	80325; 23466; 146722; 1601; 10521; 2014; 2022; 2204; 11337; 2876; 25994; 3419; 3479; 3563; 3674; 9903; 25984; 9500; 4925; 8481; 8473; 9601; 57661; 60675; 5899; 84666; 50862; 6397; 51734; 9792; 140885; 10959
**Eosinophils CIBERSORT NatMethods.2015 PMID.25822800**	Eosinophils	Signature	PMID.25822800	8809; 671; 932; 6003; 1178; 9173; 9934; 719; 10402; 246; 4688; 53829; 23569; 6036; 51744; 8807; 8972; 26030; 5996; 55024; 597; 27202; 1602; 23604; 9681; 64174; 2015; 30817; 84658; 22905; 2867; 2696; 1880; 8477; 222487; 3568; 4033; 4117; 4084; 27334; 5029; 5146; 27039; 9185; 23223; 64092; 55512; 27293; 79865; 140803; 7673
**Eosinophils Bindea Immunity.2013 PMID.24138885**	Eosinophils	Signature	PMID.24138885	32; 847; 1178; 1232; 2015; 2581; 3006; 3280; 3568; 3757; 6036; 6477; 7050; 7057; 7060; 8277; 8867; 9398; 9920; 11057; 11251; 22905; 23223; 25976; 51531; 55512; 55758; 57105; 59340; 91355; 728965; 80022
**Eosinophils Charoentong CellRep.2017 PMID.28052254**	Eosinophils	Signature	PMID.28052254	1602; 23604; 9681; 84658; 2354; 2696; 1880; 8477; 3568; 442236; 4033; 8013; 9934; 5146; 27039; 23223; 10402
**Hematopoietic.Stem.cells.ImmuneProfiles.Mouse.Human Shay PNAS.2013 PMID.23382184**	Hematopoietic	Signature	PMID.23382184	34; 284; 328; 332; 402; 444; 501; 594; 667; 672; 701; 759; 760; 783; 835; 947; 983; 1017; 1019; 1021; 1063; 1111; 1163; 1312; 1503; 1523; 1602; 1635; 1718; 1793; 1854; 1869; 1876; 1892; 1954; 1964; 2078; 2099; 2119; 2120; 2135; 2237; 2288;

© 2023 Fernandez-Martinez A et al. *JAMA Oncology.*

				2356; 2553; 2624; 2677;<br>2766; 2887; 2954; 2956;<br>2982; 3012; 3033; 3068;<br>3070; 3131; 3146; 3161;<br>3182; 3192; 3202; 3251;<br>3276; 3329; 3382; 3418;<br>3480; 3608; 3613; 3615;<br>3815; 3832; 3843; 3925;<br>3998; 4005; 4076; 4085;<br>4171; 4172; 4211; 4352;<br>4353; 4363; 4522; 4602;<br>4613; 4628; 4692; 4734;<br>4778; 4800; 4833; 5023;<br>5087; 5111; 5198; 5213;<br>5311; 5321; 5324; 5332;<br>5422; 5425; 5427; 5523;<br>5557; 5577; 5977; 5983;<br>5985; 6453; 6491; 6595;<br>6599; 6632; 6652; 6659;<br>6767; 6790; 6886; 7029;<br>7035; 7036; 7075; 7105;<br>7106; 7158; 7205; 7298;<br>7372; 7389; 7416; 7485;<br>7504; 7531; 7586; 8031;<br>8091; 8204; 8243; 8317;<br>8323; 8476; 8508; 8578;<br>8624; 8645; 8722; 8727;<br>8848; 8971; 9053; 9203;<br>9289; 9319; 9352; 9500;<br>9508; 9555; 9643; 9761;<br>9767; 9787; 9829; 9985;<br>10040; 10155; 10165;<br>10203; 10217; 10247;<br>10282; 10314; 10477;<br>10493; 10549; 10591;<br>10592; 10606; 10733;<br>10788; 10856; 10857;<br>10951; 11130; 11167;<br>11169; 11200; 11328;<br>11335; 22823; 22836;<br>22915; 22955; 23093;<br>23111; 23243; 23294;<br>23468; 23556; 23621;<br>23635; 23682; 25966;<br>26227; 26586; 27018;<br>27346; 29105; 29803;<br>29902; 30968; 50810;<br>51097; 51098; 51118;<br>51134; 51144; 51186;<br>51203; 51205; 51309;<br>51514; 51566; 51603;<br>51659; 51768; 54433;<br>54470; 54517; 54682;<br>54874; 54962; 55128;<br>55164; 55165; 55197;<br>55326; 55652; 55746;

© 2023 Fernandez-Martinez A et al. *JAMA Oncology.*

				55759; 55907; 56925; 57088; 57597; 60496; 63826; 64087; 64754; 64798; 64866; 79000; 79004; 79023; 79070; 79071; 79077; 79896; 80177; 80823; 84790; 84823; 90861; 92579; 94239; 114876; 117178; 200081; 284119; 441024; 11113; 1500; 205; 378708; 3992; 54930; 5867; 6573; 7982; 8339; 861; 100302274; 100528016; 100507855; 100526739; 100302263; 100616424; 10638; 101928717; 93655; 8343; 100506403; 102466616; 8344; 101928269; 8346; 8347
**Immune.HLA.A.F TCGA.BRCA.1198 JCI.2020 PMID.32573490**	HLA	Signature	PMID.32573490	3133; 116071; 3659; 81030; 3902; 3107; 3105; 3106; 3134; 5698; 5696; 6890; 3965; 3601; 197259
**Immune.HLA.D TCGA.BRCA.1198 JCI.2020 PMID.32573490**	HLA	Signature	PMID.32573490	972; 3108; 3109; 3122; 3115; 3113
**IFNa.Module10 Gatza ProcNatlAcadSciUSA.2010 PMID.20335537**	IFN	Signature	PMID.20335537	51479; 23780; 80830; 11119; 11118; 10384; 55337; 9473; 834; 961; 8837; 56259; 3627; 23586; 55601; 23549; 10468; 2633; 2643; 2650; 51191; 55008; 3106; 3107; 10437; 3430; 10561; 10964; 2537; 3434; 3433; 3437; 24138; 8519; 3600; 3659; 3665; 9636; 3669; 3717; 3964; 26010; 4061; 4599; 4600; 4615; 4938; 4939; 4940; 8638; 11054; 5359; 5371; 5698; 55905; 91543; 64108; 6398; 51296; 81894; 8467; 3431; 9517; 6772; 6773; 6890; 6891; 23424; 7098; 80021; 10906; 11277; 9830; 6737; 7318; 9246; 11274; 54739; 56829
**IFNg.Module11 Gatza ProcNatlAcadSciUSA.2010 PMID.20335537**	IFN	Signature	PMID.20335537	23780; 80830; 406; 528; 55509; 604; 11119; 11118; 10384; 55337; 9473; 23607; 961; 1506; 3627; 6373; 4283; 23586; 23549; 2115; 2355; 10468; 2633;

© 2023 Fernandez-Martinez A et al. *JAMA Oncology.*

				2643; 2820; 387751; 55008; 3620; 10437; 3430; 10561; 10964; 2537; 3433; 3437; 24138; 8519; 3600; 3659; 3394; 3717; 65987; 51056; 23175; 4599; 79840; 4837; 4939; 5099; 54103; 5359; 5366; 5464; 5638; 5698; 5920; 55905; 127544; 91543; 64108; 22904; 57147; 6398; 26040; 51296; 81894; 6672; 3431; 6772; 6773; 6890; 6891; 10420; 7127; 10906; 10221; 28951; 9830; 6737; 9246; 56829
**IFN.Cluster.GSEA.GP11 Fan BMCMedGenomics.2011 PMID.21214954**	IFN	Signature	PMID.21214954	6772; 10068; 54739; 567; 91543; 94240; 5696; 5698; 56829; 3430; 23586; 388646; 3627; 6352; 163351; 4283; 29126; 85441; 7318; 3959; 83666; 10379; 103; 3433; 8638; 55601; 64135; 64108; 115361; 10561; 4599; 9636; 684; 11274; 3434; 4939; 81030; 54625; 345611; 6773; 64761; 6672; 2633; 116071; 9111; 146857; 387751; 3659
**IFN Rody BreastCancerResearch.2008 PMID.19272155**	IFN	Signature	PMID.19272155	91543; 4940; 10561; 9636; 55601; 10964; 2537; 4599; 3437; 3429; 3434; 4938; 4939
**IFN.Cluster Fan BMCMedGenomics.2011 PMID.21214954**	IFN	Signature	PMID.21214954	55337; 684; 3431; 64761; 24138; 4938; 10379; 11274; 23586; 4600; 3429; 4939; 2537; 3437; 10964; 10561; 3434; 9636; 4599; 91543; 4940; 55008; 51191; 55601; 5359; 9246; 54809; 64108; 54739; 9111; 8519; 10410; 3669; 5721; 5720; 3665
**IFN.3.ImmLandscape Wolf PlosOne.2014 PMID.24516633**	IFN	Signature	PMID.24516633	3627; 23586; 55601; 51191; 55008; 3429; 10561; 10964; 2537; 64135; 3434; 3433; 3437; 9636; 4599; 4938; 4939; 4940; 8638; 91543; 64108; 54809; 6772; 6890
**IFN.5.ImmLandscape Wolf PlosOne.2014 PMID.24516633**	IFN	Signature	PMID.24516633	27299; 9447; 597; 330; 10384; 6357; 6362; 6363; 6352; 6355; 1236; 930; 952; 917; 969; 973; 942; 926; 64581; 56253; 1075;

© 2023 Fernandez-Martinez A et al. *JAMA Oncology.*

				1520; 3627; 6373; 10563; 4283; 10663; 1536; 9595; 2219; 2533; 2633; 10578; 29909; 2841; 3002; 2999; 3003; 10866; 29851; 3620; 3458; 10261; 3600; 3561; 9235; 3659; 3669; 3702; 3902; 27074; 3932; 10288; 50536986; 4067; 51237; 4818; 80896; 11040; 7941; 51316; 5450; 5698; 5778; 64108; 864; 64092; 23231; 6402; 57823; 56833; 11262; 6890; 22797; 608; 50852; 10537; 8875; 7453; 79413
**IFN.Pathway ImSig.Nirmal CancerImmunolRes. 2018 PMID.30266715**	IFN	Signature	PMID.30266715	57169; 85441; 10964; 57674; 85363; 11274; 9636; 55601; 94240; 4599; 4600; 116071; 684; 151636; 55008; 51191; 10561; 3428; 84875; 51056; 6614; 64761; 54625; 115361; 2633; 64135; 64108; 8519; 4940; 5371; 91543; 4939; 3431; 219285; 3430; 84941; 129607; 6890; 51246; 26270; 54739; 79132; 9246; 51131; 6772; 10346; 6737; 6773; 55337; 5698; 23586; 10379; 3437; 3433; 8638; 3434; 8542; 27074; 83666; 24138; 3665; 54809; 10906; 80830; 441108; 2537
**IFN.Score.module3 TCGA Immunity.2018 PMID.29628290**	IFN	Signature	PMID.29628290	10561; 10964; 23586; 2537; 3429; 3433; 3434; 3437; 8375; 8374; 8376; 3627; 4599; 4938; 4939; 4940; 51191; 54809; 55008; 55601; 64108; 64135; 6772; 6890; 8638; 91543; 9636
**Immune.IFN TCGA.BRCA.1198 JCI.2020 PMID.32573490**	IFN	Signature	PMID.32573490	10379; 24138; 51191; 55008; 55601; 54809; 3429; 2537; 9636; 23586; 64135; 3434; 10561; 10964; 3433; 3437; 129607; 91543; 4939; 4599; 8638; 4938; 4940; 54625; 83666; 6772; 11274; 54739; 4600; 64761; 5359; 9246; 6672; 3431

© 2023 Fernandez-Martinez A et al. *JAMA Oncology.*

**IgG Rody BreastCancerResearch.2008 PMID.19272155**	**Ig**	Signature	PMID.19272155	391427; 96610; 339562; 28902; 651961; 90925; 651629; 91353; 28299; 28461; 5450; 28831; 91316; 3538; 28793; 3514; 28815; 3500; 3507; 28786; 3495; 3493; 3535; 3492; 3537; 652745
**IGG.Cluster Fan BMCMedGenomics.2011 PMID.21214954**	**Ig**	Signature	PMID.21214954	939; 3561; 973; 11040; 5450; 3514; 28793; 3535; 3512; 608; 4917; 3576; 3107; 54900
**Ig TCGA.BRCA.1198 Cell.2015 PMID.26451490**	**Ig**	Signature	PMID.26451490	28904; 28467; 28875; 28426; 28776; 28474; 28919; 28424; 28923; 28874; 28420; 28784; 28423; 28817; 28937; 28457; 28876; 28943; 28778; 28400; 28396; 28793; 28434; 28391; 28941; 28938; 28935; 28408; 28394; 28797; 28914; 28395; 28465; 28809; 28450; 28822; 28448; 28933; 28468; 28388; 28813; 28921; 28444; 28815; 28893; 28392; 28299; 28442; 28912; 28908; 28913; 28825; 28439; 28796
**Immune.Suppression Kardos JCIInsight.2016 PMID.27699256**	**Immune checkpoint molecules**	Signature	PMID.27699256	29126; 3620; 356; 1493; 5133; 3902; 84868; 80380; 3586; 7040; 5743; 4513
**CD274.Single.Gene Hollern Cell.2019 PMID.31730857**	**Immune checkpoint molecules**	Gene	PMID.31730857	29126; 29126; 29126; 29126
**CTLA4.Single.Gene Hollern Cell.2019 PMID.31730857**	**Immune checkpoint molecules**	Gene	PMID.31730857	1493; 1493; 1493; 1493
**PDCD1.Single.Gene Pare AnnOncol.2019 PMID.30165419**	**Immune checkpoint molecules**	Gene	PMID.30165419	5133; 5133; 5133; 5133
**Immunosuppression Petitprez Nature.2020 PMID.31942077**	**Immune suppression**	Signature	PMID.31942077	7043; 6387; 7040; 3956
**Stromal.Inflammation Heng JPathol.2017 PMID.27861902**	**Inflammation**	Signature	PMID.27861902	1493; 27436; 50943; 2633; 9812; 11040; 23231; 9047; 56833; 201633; 79413
**Wound.Healing Chang PlosBiol.2004 PMID.14737219**	**Inflammation**	Signature	PMID.14737219	292; 1111; 1178; 1326; 1633; 1663; 1841; 1978; 1981; 2023; 2107; 2171; 2193; 2224; 2923; 3015; 3037; 3146; 3151; 3157;

© 2023 Fernandez-Martinez A et al. *JAMA Oncology.*

				3181; 3182; 3422; 3638; 3655; 3988; 3992; 4172; 4176; 4240; 4331; 4478; 4521; 4597; 4603; 4605; 4830; 5036; 5214; 5216; 5329; 5352; 5427; 5447; 5688; 5695; 5702; 5708; 5718; 5757; 5805; 5814; 5902; 5983; 6182; 6184; 6319; 6382; 6427; 6434; 6566; 6590; 6611; 6626; 6627; 6628; 6631; 6632; 6635; 6675; 6713; 6723; 6747; 6921; 6993; 7077; 7127; 7167; 7277; 7283; 7372; 7416; 7866; 7965; 7980; 8202; 8836; 9235; 9263; 9276; 9415; 9521; 9601; 9818; 9821; 10056; 10063; 10204; 10301; 10432; 10452; 10465; 10534; 10535; 10606; 10733; 10799; 10951; 10969; 11169; 11325; 11333; 11340; 22919; 22948; 23175; 23406; 23558; 23603; 23646; 26207; 26471; 27020; 27258; 28957; 29088; 29796; 50484; 51128; 51192; 51253; 51330; 51444; 51491; 51534; 51668; 51670; 51696; 51728; 54206; 54858; 55038; 55055; 55110; 55186; 55270; 55631; 55646; 55651; 55706; 55835; 55839; 55884; 56926; 56942; 57122; 57213; 57486; 57523; 64750; 79007; 79172; 79682; 79902; 84823; 90861; 201254
**TGFB.score TCGA Immunity.2018 PMID.29628290**	**Inflammation**	Signature	PMID.29628290	4314; 65108; 3482; 3912; 6678; 2335; 3676; 6608; 4327; 3696; 3678; 4811; 7076; 6405; 23433; 1499; 4313; 5054; 2048; 1307; 1969; 3371; 3728; 3675; 6934; 1281; 1004; 7482; 8754; 1832; 3339; 392; 3693; 3488; 396; 4035; 3485; 1495; 10234; 4323; 4756; 1946; 3690; 2049; 960; 3487; 7132; 5879; 5829; 5327; 1295; 7479;

© 2023 Fernandez-Martinez A et al. *JAMA Oncology.*

				3486; 387; 2050; 4312; 5058; 9112; 7058; 1462; 4326; 966; 1857; 388; 1293; 4853; 682; 4320; 1278; 7791; 390; 7057; 391; 3383; 3910; 1855; 5062; 3689; 1291; 2245
**Immune.FOS.JUN.IL 6 TCGA.BRCA.1198 JCI.2020 PMID.32573490**	**Inflammation**	Signature	PMID.32 573490	8013; 3569; 9021; 467; 1959; 64651; 1843; 1958; 2354; 7538; 3164; 3725; 1839; 23645
**Macrophages.M0 CIBERSORT NatMethods.2015 PMID.25822800**	**Macrophages**	Signature	PMID.25 822800	366; 728; 968; 51313; 3101; 10261; 4688; 51296; 8876; 8740; 54; 27299; 79365; 79839; 6362; 6367; 6354; 1116; 1296; 1435; 2921; 6374; 1593; 81501; 2239; 8685; 4318; 7941; 5473; 25797; 56833; 84561; 54209
**Macrophages.M1 CIBERSORT NatMethods.2015 PMID.25822800**	**Macrophages**	Signature	PMID.25 822800	366; 51296; 27299; 1116; 200315; 11026; 10288; 64127; 51284; 51311; 6352; 80830; 8651; 3902; 3559; 5739; 10563; 79931; 1236; 952; 43; 80833; 408; 6363; 6355; 958; 1193; 3627; 6373; 4283; 1594; 79132; 10148; 2687; 8820; 3620; 10964; 643314; 8942; 27074; 51365; 83937; 91543; 6614; 6504; 11182; 7130; 54795
**Macrophages.M2 CIBERSORT NatMethods.2015 PMID.25822800**	**Macrophages**	Signature	PMID.25 822800	51296; 27299; 1116; 51311; 6355; 1193; 10148; 2687; 6614; 968; 51313; 6362; 54209; 199; 5199; 50856; 2242; 2535; 64231; 53829; 51314; 920; 246; 6357; 6358; 6368; 30835; 10462; 1414; 55691; 2952; 3269; 3357; 80896; 80380; 5973; 81029
**Macrophages Bindea Immunity.2013 PMID.24138885**	**Macrophages**	Signature	PMID.24 138885	348; 586; 950; 968; 1116; 1118; 1296; 1536; 2012; 2230; 2239; 2335; 2760; 3730; 4199; 4481; 5730; 6354; 6374; 6447; 6819; 8685; 8832; 9332; 10533; 23601; 26064; 26577; 51338; 58511; 81035; 259230; 1513
**Macrophages.M2 Ghassabeh Blood.2006 PMID.16556895**	**Macrophages**	Signature	PMID.16 556895	383; 84666; 7941; 5660; 6414; 54209; 5376; 10462; 1118; 27159; 374868; 999; 2350

© 2023 Fernandez-Martinez A et al. *JAMA Oncology.*

**Macrophages.Th1.Cluster Iglesia CCR.2014 PMID.24916698**	Macrophages	Signature	PMID.24916698	26191; 5330; 50856; 3111; 752; 117289; 5552; 1436; 945; 713; 714; 712; 1520; 146722; 3394; 388325; 4332; 313; 57705; 8832; 22797; 1536; 5294; 11309; 3113; 3115; 3122; 3109; 3108; 972; 64092; 219972; 7133; 9938; 257106; 84433; 64231; 64098; 8477; 80231; 3594; 286336; 221472; 6404; 951; 3059; 7454; 4689; 3689; 83706; 6688; 4542; 27128; 79626; 199; 7940; 124460; 695; 3937; 5788; 2124; 3071; 963; 5341; 920; 54440; 3587; 1794; 2533; 10320; 1234; 6693; 64333; 10859; 11006; 2207; 9056; 3903; 84868; 7805; 942; 23533; 139716; 64926; 147138; 89857; 54518; 6503; 3635; 126364; 27180; 27036; 968; 3687; 55803; 719; 2123; 7456; 10875; 10261; 10288; 51411; 89790; 51311; 6039
**Macrophages.ImmuneProfiles.Mouse.Human Shay PNAS.2013 PMID.23382184**	Macrophages	Signature	PMID.23382184	136; 526; 719; 1026; 1051; 1317; 1436; 1462; 1508; 1827; 1848; 1861; 2131; 4035; 5271; 5326; 6051; 6237; 6256; 6614; 7045; 7056; 7133; 7941; 8522; 8754; 9056; 9732; 9777; 9935; 10577; 10855; 10924; 22918; 26509; 51311; 55022; 55160; 55486; 64127; 206358; 219654
**Macrophages Charoentong CellRep.2017 PMID.28052254**	Macrophages	Signature	PMID.28052254	1268; 1269; 3101; 3269; 2206; 199; 10409; 6346; 6358; 6368; 10344; 124599; 920; 1414; 10209; 1973; 51313; 2242; 2357; 2358; 23401; 55691; 2535; 2850; 27202; 3176; 10261; 80896; 6036; 51296; 51314; 8876; 81029
**Macrophages.Monocytes.CSF1.Response Beck CCR.2009 PMID.19188147**	Macrophages	Signature	PMID.19188147	113; 241; 272; 341; 348; 55843; 409; 567; 695; 80183; 79630; 719; 834; 6347; 6352; 9034; 9332; 945; 962; 1043; 963; 8832; 942; 925; 976; 51816;

© 2023 Fernandez-Martinez A et al. *JAMA Oncology.*

				11151; 54504; 1436; 1075; 1520; 10659; 7852; 1536; 1612; 1794; 2123; 2124; 2207; 2209; 2212; 2213; 2214; 10875; 2268; 2313; 752; 23048; 2359; 2533; 55303; 79158; 10457; 3055; 3059; 3108; 3123; 23526; 3428; 10437; 10320; 3587; 3606; 3575; 3689; 3903; 7805; 3936; 3937; 10184; 11006; 4033; 7940; 9450; 4067; 4069; 10461; 4332; 4354; 4478; 4542; 4688; 3071; 10577; 80896; 26031; 5294; 5336; 5341; 9595; 5788; 9770; 83937; 6039; 864; 25939; 64092; 6402; 6404; 10019; 6507; 9056; 11262; 6850; 6916; 7097; 7128; 7133; 10673; 11326; 7456; 1514
**Macrophages ImSig.Nirmal CancerImmunolRes. 2018 PMID.30266715**	Macrophages	Signature	PMID.30266715	59342; 140; 4481; 51393; 6688; 51311; 55281; 9034; 313; 4125; 2215; 79895; 58475; 3903; 728; 23643; 1240; 597; 51816; 1520; 9332; 712; 10261; 713; 942; 10673; 409; 11006; 4332; 7941; 1508; 11326; 7127; 29887; 719; 1230; 27128; 10437; 3689; 2359; 64747; 79630; 972; 3684; 968; 7133; 1890; 7805; 3687; 2209; 3101; 2210; 80301; 2207; 3128; 920; 717; 1318; 10457; 7305; 10062; 1436; 51338; 3988; 6916; 80896; 3071; 4542; 56833; 11309; 1536; 11314; 64581; 1806; 2212; 51296; 27299
**Immune.GIMAP.IL16 TCGA.BRCA.1198 JCI.2020 PMID.32573490**	Macrophages	Signature	PMID.32573490	54518; 55843; 1439; 9938; 170371; 1193; 2313; 55303; 168537; 474344; 55340; 4242; 139716; 7133; 4033; 10875; 219972; 3635; 3603
**Mast.cells.Activated CIBERSORT NatMethods.2015 PMID.25822800**	Mastocytes	Signature	PMID.25822800	945; 2205; 84561; 8809; 671; 932; 6003; 79895; 1178; 1215; 1359; 1511; 3067; 27306; 2206; 4602; 50508; 4914; 5023; 5553; 5874; 29091; 7177; 6364; 6346; 2921; 3002; 1437; 6351; 3562; 3198; 3552;

© 2023 Fernandez-Martinez A et al. *JAMA Oncology.*

				3553; 9173; 3567; 81698; 115123; 7006
**Mast.cells.Resting CIBERSORT NatMethods.2015 PMID.25822800**	Mastocytes	Signature	PMID.25822800	945; 2205; 84561; 8809; 2672; 671; 1088; 932; 6489; 6003; 26228; 9934; 9508; 154; 79895; 55589; 719; 1178; 1215; 1359; 10321; 1511; 79843; 400451; 3067; 27306; 4056; 2206; 4602; 50508; 4914; 5023; 54852; 5553; 5874; 23176; 29091; 7177
**Mast.cells Bindea Immunity.2013 PMID.24138885**	Mastocytes	Signature	PMID.24138885	116; 190; 794; 946; 1088; 1215; 1359; 1511; 1991; 2206; 2624; 3067; 3248; 3815; 4013; 4353; 5553; 5742; 6571; 6886; 7177; 7857; 10257; 27306; 57419; 57460; 64499; 79083; 339524; 4129
**Mast.cell Charoentong CellRep.2017 PMID.28052254**	Mastocytes	Signature	PMID.28052254	1359; 9508; 55843; 1215; 1368; 1511; 1960; 2219; 2512; 3310; 3680; 29992; 5743; 6037; 6275; 27181; 6532
**MDSC.Granulocytic Youn LeukocBiol.2012 PMID.21954284**	MDSC	Signature	PMID.21954284	100; 9133; 820; 8698; 2298; 2330; 2354; 2812; 3512; 3929; 4048; 4057; 4155; 4353; 5004; 5657; 641371; 55143; 8434; 1687; 55; 1992; 51449; 11065; 27333; 29942; 57509; 3039; 140460; 932; 55796; 55789; 353189; 10420; 9055; 25903
**MDSC.Neutrophil Youn LeukocBiol.2012 PMID.21954284**	MDSC	Signature	PMID.21954284	894; 1154; 1234; 2919; 2921; 3383; 3557; 3569; 3627; 4023; 4323; 4502; 4794; 5196; 5900; 6275; 6281; 6354; 6385; 6648; 6696; 7057; 7124; 7185; 9641; 23328; 23657; 55686; 56895
**MDSC.Tumor.Macrophages Schlecker JImmunol.2012 PMID.23152559**	MDSC	Signature	PMID.23152559	6351; 6348; 6354; 338442; 5008; 10409; 6369; 383; 967; 1339; 9034; 6696; 10022; 7070; 4323; 5996; 3303; 29923; 3553; 339210; 2920; 4360; 4322; 2919; 467; 3932; 1026; 9047; 2012; 929; 28984; 1601; 10912; 6385; 4283; 23179; 29126; 4643; 1263; 58191; 8877; 1958; 713; 8870; 1154; 101; 7124; 4818; 730249; 3123; 3725;

© 2023 Fernandez-Martinez A et al. *JAMA Oncology.*

				93978; 64651; 22822; 7535; 1844; 7052; 93099; 3557; 5209; 1649; 9046; 3783; 6319; 388325; 7371; 714; 23767; 23646; 9518; 7538; 54498; 400916; 8294; 1266; 2155; 9619; 6513; 4000; 7167; 54855; 1843; 23166; 10938; 8531; 338339; 2867; 7185; 2584; 23645; 7422; 84709; 90637; 3162; 28966; 10630; 1847; 712; 26064; 5236; 23657; 23428; 84331; 7076; 415116; 8111; 10769; 11172; 8354; 3575; 90522; 112399; 81671; 84868; 171177; 84275; 1647; 84803; 3939; 9500; 290; 4794; 1846; 7852; 366; 3726; 5866; 6004; 124540
**MDSC.Tumor Schlecker JImmunol.2012 PMID.23152559**	MDSC	Signature	PMID.23 152559	123; 290; 383; 467; 712; 713; 714; 817; 839; 929; 958; 1026; 1154; 1234; 1462; 1476; 1522; 1601; 1643; 1827; 1845; 1846; 1893; 1910; 1958; 2012; 2078; 2155; 2159; 2213; 2309; 2549; 2697; 2799; 2810; 2919; 2920; 3119; 3123; 3162; 3303; 3304; 3383; 3557; 3617; 3680; 3725; 4000; 4239; 4261; 4283; 4322; 4323; 4360; 4794; 5008; 5025; 5026; 5146; 5209; 5236; 5328; 5553; 5597; 5641; 5817; 5866; 5973; 5996; 6347; 6348; 6351; 6354; 6369; 6385; 6535; 6643; 6692; 6696; 7052; 7076; 7124; 7167; 7185; 7431; 7982; 8360; 8364; 8553; 8808; 8835; 8877; 8892; 8991; 9034; 9043; 9046; 9308; 9518; 9522; 9546; 9641; 9732; 9788; 9797; 9934; 9953; 10046; 10062; 10268; 10312; 10417; 10457; 10461; 10493; 10630; 10915; 11172; 11186; 11238; 11282; 11316; 11346; 22862; 23049; 23099; 23166; 23179; 23207; 23216;

© 2023 Fernandez-Martinez A et al. *JAMA Oncology.*

				23428; 23516; 23552; 23646; 23767; 25819; 25932; 25977; 26054; 26064; 27033; 27043; 28966; 29126; 29886; 29923; 29947; 29967; 30837; 50865; 51365; 51573; 51586; 54033; 54206; 54476; 54756; 54810; 54855; 55198; 55339; 55635; 55748; 55860; 55917; 56975; 57020; 57084; 57458; 57600; 58191; 59338; 64174; 64757; 64943; 65083; 79632; 79768; 80070; 80380; 80381; 80755; 80821; 81671; 84002; 84301; 84333; 84679; 84709; 84868; 90637; 91768; 93099; 93978; 112770; 116039; 127281; 128506; 140809; 145389; 151835; 157378; 160622; 161142; 171177; 196743; 222171; 259307; 285590; 339366; 342667; 376267; 377677; 388325; 388595; 401548; 415116; 642623
**MDSC Charoentong CellRep.2017 PMID.28052254**	MDSC	Signature	PMID.28052254	929; 5660; 729230; 914; 942; 7852; 2212; 2213; 2214; 83706; 63940; 10068; 3566; 3683; 3684; 64098; 5732; 80142; 6279; 6280
**MHC.11genes Forero CancerImmunolRes. 2016 PMID.26980599**	MHC	Signature	PMID.26980599	4261; 972; 3113; 3115; 3116; 3117; 3123; 3127; 3128; 1512; 8648; 100290966; 731247
**MHC.24genes Forero CancerImmunolRes. 2016 PMID.26980599**	MHC	Signature	PMID.26980599	4261; 972; 3113; 3115; 3116; 3117; 3123; 3127; 3128; 1512; 8648; 100290966; 731247; 913; 2217; 3861; 57121; 89846; 6844; 120892; 4154; 4916; 84265; 5730; 6451; 9760; 399472
**MHC.I Rody BreastCancerResearch.2008 PMID.19272155**	MHC	Signature	PMID.19272155	3135; 3105; 3106; 3107; 3134
**MHC.II Rody BreastCancerResearch.2008 PMID.19272155**	MHC	Signature	PMID.19272155	1520; 972; 3123; 3117; 3109; 3113; 3115; 3108; 3122; 3937; 5552; 5788

© 2023 Fernandez-Martinez A et al. *JAMA Oncology.*

**MHC.I.CoreGenes Lauss NatCommun.2017 PMID29170503**	MHC	Signature	PMID.29170503	3105; 3106; 3107; 6891; 84166; 6890; 5698; 5696; 567
**CD68.Cluster Iglesia CCR.2014 PMID.24916698**	Monocytes	Signature	PMID.24916698	126364; 27180; 27036; 968; 3687; 55803; 719
**Monocytes CIBERSORT NatMethods.2015 PMID.25822800**	Monocytes	Signature	PMID.25822800	8875; 2354; 199; 200315; 366; 432; 433; 683; 728; 729230; 912; 945; 968; 978; 5199; 51363; 50856; 64581; 9586; 1441; 51313; 2219; 2242; 2357; 2535; 3055; 3101; 3176; 10855; 10261; 11027; 11026; 10288; 7940; 4210; 4332; 64231; 4688; 4778; 114548; 64127; 53829; 23569; 6036; 6039; 6283; 51296; 7097; 51284; 51311; 7380; 8876
**Tcells.Effector.Memory Bindea Immunity.2013 PMID.24138885**	Monocytes	Signature	PMID.24138885	1231; 2130; 2313; 4058; 4210; 4776; 5616; 6904; 6955; 7430; 9779; 10000; 10521; 27099; 81544
**Monocytes.Dendritic.cell.Metagene Miller GenomeBiol.2013 PMID.23618380**	Monocytes	Signature	PMID.23618380	3113; 7940; 3133; 3115; 3119; 3108; 3122; 3123; 972
**Monocytes Charoentong CellRep.2017 PMID.28052254**	Monocytes	Signature	PMID.28052254	1072; 4155; 71; 308; 432; 433; 526; 912; 5199; 1486; 9802; 326342; 3097; 10320; 65108; 4324; 10908; 10084; 51368; 7009; 7380
**Monocytes ImSig.Nirmal CancerImmunolRes.2018 PMID.30266715**	Monocytes	Signature	PMID.30266715	7940; 2268; 199; 7077; 2242; 9056; 22918; 1200; 3162; 2219; 7045; 23313; 79168; 29108; 5265; 391; 29992; 5660; 3055; 150372; 84034; 126364; 57085; 2799; 4853; 10288; 11025; 2896; 945; 84106; 84898; 1509; 146722; 1462; 9473; 64115; 929
**Monocytes.Dendritic.25genes Miller GenomeBiol.2013 PMID.23618380**	Monocytes	Signature	PMID.23618380	241; 920; 972; 1436; 1536; 1794; 2123; 2124; 10875; 3109; 3113; 3115; 3117; 3119; 3122; 3123; 3133; 7462; 7940; 5788; 5552; 3118; 3127; 3128; 3108
**CSF1.Response TCGA Immunity.2018 PMID.29628290**	Monocytes	Signature	PMID.29628290	11151; 4332; 9034; 9056; 3108; 2533; 6039; 7097; 1075; 11006; 9595; 1520; 83937; 4478; 1536; 7805;

© 2023 Fernandez-Martinez A et al. *JAMA Oncology.*

				1794; 2209; 2124; 113; 962; 55843; 409; 6850; 695; 7128; 2212; 11326; 2359; 3587; 3428; 3689; 3575; 6916; 752; 2313; 9770; 4069; 9332; 976; 6347; 2213; 10461; 8832; 963; 942; 23526; 1514; 2123; 7133; 7852; 3936; 25939; 54504; 3123; 80183; 55303; 64092; 5336; 26031; 925; 864; 2214; 272; 4542; 51816; 4067; 4354; 4033; 10875; 3071; 3059; 6402; 834; 6404; 945; 10457; 4688; 23048; 3606; 567; 11262; 2207; 3937; 9450; 3903; 10437; 10673; 7929; 79407929; 7940; 2268; 80896; 5341; 6352; 5788; 79158; 6507; 3055; 10577; 719; 5294; 1612; 241; 1436; 10659; 348; 341; 1043; 10184; 79630; 10320; 10019; 7456
**Immune.CD34.TIE1 TCGA.BRCA.1198 JCI.2020 PMID.32573490**	**Monocytes**	Signature	PMID.32573490	54810; 6403; 5243; 9452; 22915; 6358; 857; 6886; 79843; 22918; 2078; 947; 1003; 80177; 1901; 7075; 10252; 128553; 5168
**HCK Rody BreastCancerResearch.2008 PMID.19272155**	**Monocytes**	Signature	PMID.19272155	64231; 11309; 51338; 7940; 199; 7305; 1794; 2207; 6039; 9056; 4332; 4688; 713; 7805; 10437; 22797; 9332; 3903; 942; 3689; 3055; 712; 1230
**Influenza.11genes Khatri Immunity.2015 PMID.26682989**	**Monocytes**	Signature	PMID.26682989	952; 51191; 55008; 2537; 64135; 3959; 4061; 4599; 64761; 64108; 81030
**Neutrophils.Activated.Blood Janiszewska NatCellBiol.2019 PMID.31263265**	**Neutrophils**	Signature	PMID.31263265	3043; 1514; 3039; 2791; 3043; 10398; 2815; 11313; 27013; 928; 51635; 10482; 3655; 5473; 1191; 8294; 10170; 64805; 65213; 4201; 6840; 11182; 3555; 7850; 653361; 5196; 3181; 84419; 6503; 3040; 4601; 820; 4792; 4282; 1831; 4001; 65125; 51274; 1611; 9246; 11345; 89875; 3187; 51499; 976; 4940; 259230; 26469; 23521; 54625; 54926; 51206; 4713; 6467; 7979; 128346; 10924; 6271; 3480; 5269; 1475;

© 2023 Fernandez-Martinez A et al. *JAMA Oncology.*

				10906; 1075; 217; 6146; 6733; 91543; 64500; 283349; 2214; 6137; 10541; 8848; 27086; 6233; 85027; 10260; 4726; 11329; 51324; 2280; 2289; 26262; 9034; 1116; 10390; 23118; 7430; 1647; 29780; 55602; 2098; 90355; 6191; 9636; 51266; 22889; 6001; 7090; 6205; 11079; 1656; 506; 57533; 3840; 517; 9588; 6130; 8407; 50486; 6210; 10294; 3958; 9595; 6009; 8493; 8347; 2014; 51194; 10959; 125965; 2812; 903; 57018; 9289; 5688; 23528; 2123; 196264; 5577; 94097; 6161; 9541; 1990; 10634; 6228; 55500; 11243; 83662; 60685; 2920; 4540
**Neutrophils.Activated.Lung Janiszewska NatCellBiol.2019 PMID.31263265**	**Neutrophils**	Signature	PMID.31263265	467; 8832; 79145; 143384; 115817; 3265; 80153; 92241; 123; 6222; 84919; 9698; 10905; 334; 23741; 2778; 51324; 23048; 10999; 63935; 1479; 11052; 22898; 54542; 7884; 258010; 23360; 63931; 64961; 23412; 5711; 58476; 26292; 4520; 9686; 1613; 7704; 85439; 26156; 9748; 56688; 5439; 9169; 6856; 10011; 6414; 64965; 79095; 151188; 10010; 5782; 4938; 3656; 55180; 60626; 388272; 4597; 64943; 4200; 8260; 139716; 84265; 8295; 255967; 119504; 22862; 5771; 9658; 23602; 57175; 79077; 6137; 10923; 1175; 54522; 91373; 10906; 2631; 79705; 55331; 9274; 203328; 4671; 51527; 4677; 8199; 84557; 3692; 10451; 11021; 51028; 51021; 64959; 2314; 4214; 4089; 51315; 3397; 53938; 3028; 1435; 79982; 6497; 389203; 55144; 155185; 79042; 4205; 5428; 1431; 5376; 140735; 22890; 51586; 7936; 79143; 1656; 29896; 196264; 54407;

© 2023 Fernandez-Martinez A et al. *JAMA Oncology.*

				23476; 8655; 2533; 23168; 79637; 10527; 64777; 10713; 1192; 57620; 91746; 4659; 56259; 84306; 8575; 81704; 6503; 59339; 80790; 122622; 7534; 8344; 5048; 55904; 65117; 57140; 58159; 10625; 10857; 9061; 51499; 9683; 136647; 54069; 81555
**Neutrophils CIBERSORT NatMethods.2015 PMID.25822800**	Neutrophils	Signature	PMID.25822800	1178; 9934; 4688; 53829; 23569; 8807; 8972; 26030; 64174; 30817; 84658; 2867; 222487; 4117; 4084; 9185; 79865; 7130; 199; 10261; 1116; 51311; 5199; 366; 200315; 10288; 728; 8876; 25797; 8875; 978; 51363; 9586; 1441; 2357; 11027; 7940; 4210; 4332; 4778; 6283; 7097; 820; 79908; 838; 1232; 1084; 3577; 3579; 55924; 2215; 2358; 3034; 3310; 64386; 8993; 79689; 8794; 54210; 55350
**Neutrophils Bindea Immunity.2013 PMID.24138885**	Neutrophils	Signature	PMID.24138885	249; 683; 1084; 1441; 2204; 2215; 2357; 2358; 3577; 3579; 3772; 4051; 4311; 5142; 6283; 6583; 8291; 8347; 8778; 8794; 8972; 9586; 9895; 10288; 10855; 22918; 50486; 51312; 55313; 55350; 83716
**Granulocytes.ImmuneProfiles.Mouse.Human Shay PNAS.2013 PMID.23382184**	Neutrophils	Signature	PMID.23382184	306; 366; 383; 526; 706; 719; 728; 820; 928; 929; 1116; 1436; 1441; 1675; 2896; 3082; 3162; 3176; 3934; 4057; 4814; 4973; 5154; 5329; 5724; 6280; 6403; 6688; 6813; 6916; 7057; 7077; 7097; 7099; 7130; 8301; 8972; 9764; 9826; 10159; 10170; 10457; 10562; 10634; 11237; 23569; 23601; 26253; 50486; 51257; 51311; 51734; 54210; 55350; 55625; 55647; 55793; 56729; 79689; 83716
**Neutrophils.MCP Petitprez**	Neutrophils	Signature	PMID.31942077	762; 1084; 3577; 3579; 4051; 2215; 3034; 3772;

© 2023 Fernandez-Martinez A et al. *JAMA Oncology.*

**Nature.2020 PMID.31942077**				1955; 51312; 79689; 9895; 7090; 8794; 55350
**Neutrophils Charoentong CellRep.2017 PMID.28052254**	**Neutrophils**	Signature	PMID.28052254	4117; 64386; 200315; 79908; 55924; 838; 978; 51363; 9586; 3577; 3579; 2867; 3034; 8972; 6283; 79689; 8794; 55350
**Neutrophils ImSig.Nirmal CancerImmunolRes.2018 PMID.30266715**	**Neutrophils**	Signature	PMID.30266715	10135; 23258; 10154; 6279; 366; 10581; 57580; 6280; 85464; 6777; 2357; 7097; 84984; 3579; 7099; 3759; 23765; 9750; 7132; 399844; 2180; 8291; 11027; 201799; 126308; 5899; 1441; 604; 284996; 1912; 8837; 144423; 80216; 3985; 4689; 11240; 10409; 6774; 7056; 9103; 8972; 51317; 51312; 692205
**NK.Activated CIBERSORT NatMethods.2015 PMID.25822800**	**NK cells**	Signature	PMID.25822800	919; 3932; 4068; 3004; 3560; 3820; 79037; 3002; 6352; 3001; 8530; 10578; 2999; 3824; 22914; 5551; 5729; 28526; 51744; 8807; 7535; 10225; 924; 5790; 4818; 3595; 1521; 3823; 51348; 8809; 3802; 3812; 10004; 79899; 53637; 30009; 7294; 29909; 2841; 1803; 4050; 4049; 969; 1437; 3458; 9402; 5732; 259197; 60489; 80830; 6351; 894; 1021; 356; 3805; 3809; 5008; 8651; 8740
**NK.Resting CIBERSORT NatMethods.2015 PMID.25822800**	**NK cells**	Signature	PMID.25822800	914; 919; 3932; 4068; 3004; 28639; 3560; 3820; 79037; 3002; 6352; 3001; 3003; 8530; 10578; 2999; 3824; 22914; 5551; 5729; 28526; 11126; 51744; 2672; 8807; 7535; 10225; 924; 5790; 4818; 3595; 1521; 3823; 8302; 51348; 566; 671; 820; 92211; 1088; 1669; 1991; 8809; 3802; 3812; 8972; 932; 10004; 51314; 79156; 79899; 53637; 30009; 7011; 55020; 7294; 7694
**NK.CD56bright Bindea Immunity.2013 PMID.24138885**	**NK cells**	Signature	PMID.24138885	758; 1846; 2302; 6236; 6375; 8398; 8567; 10299; 57876; 83696; 254531

© 2023 Fernandez-Martinez A et al. *JAMA Oncology.*

**NK.CD56dim Bindea Immunity.2013 PMID.24138885**	NK cells	Signature	PMID.24138885	2975; 3002; 3804; 3806; 3807; 3810; 3811; 3812; 3813; 10417; 50615; 53637; 55020; 56937; 115653
**NK Bindea Immunity.2013 PMID.24138885**	NK cells	Signature	PMID.24138885	104; 219; 323; 596; 988; 2527; 2574; 2778; 3488; 5710; 6375; 6693; 6846; 6915; 7755; 8572; 8817; 8888; 9437; 9902; 11155; 22924; 25959; 27308; 51343; 55503; 55671; 57716; 64129; 64225; 64924; 65988; 79867; 84436; 259230; 643313; 730096
**Tcells.Central.Memory Bindea Immunity.2013 PMID.24138885**	NK cells	Signature	PMID.24138885	360; 472; 841; 1540; 1678; 2533; 3187; 4214; 4297; 4747; 4775; 5108; 6482; 6638; 7294; 8287; 8556; 8675; 8821; 9057; 9397; 11278; 22990; 23059; 23177; 23348; 55729; 58487; 80012; 80342; 85021; 202134; 246721; 283970; 441155; 116828; 27086; 55269; 84663
**NK.ImmuneProfiles.Mouse.Human Shay PNAS.2013 PMID.23382184**	NK cells	Signature	PMID.23382184	598; 864; 1521; 2151; 3001; 3140; 3458; 3560; 3673; 3824; 3982; 4818; 5782; 6574; 7008; 8459; 8530; 8807; 8809; 9437; 11030; 11092; 11126; 11145; 11178; 11285; 30009; 54438; 54843; 79158; 81563; 83988; 254531
**NK.CD56bright Charoentong CellRep.2017 PMID.28052254**	NK cells	Signature	PMID.28052254	1001; 10866; 3067; 18; 64919; 56935; 56951; 92211; 1193; 9167; 148327; 1435; 1459; 1475; 1476; 1503; 1509; 1594; 25853; 8655; 10480; 84173; 957; 151194; 165186; 10468; 2624; 2766; 7107; 84717; 23462; 3198; 9653; 9957; 3892; 64223; 140465; 79612
**NK.CD56dim Charoentong CellRep.2017 PMID.28052254**	NK cells	Signature	PMID.28052254	2902; 3806; 100132285; 3810; 22977; 1593; 57696; 8445; 55890; 3133; 50615; 4854; 64840; 5704; 6168; 7378
**NK Charoentong CellRep.2017 PMID.28052254**	NK cells	Signature	PMID.28052254	729447; 2635; 7940; 10000; 558; 596; 684; 988; 1000; 56253; 1438; 1522;

© 2023 Fernandez-Martinez A et al. *JAMA Oncology.*

				2919; 9267; 1616; 160851; 54567; 1806; 2065; 50848; 100132948; 81553; 356; 2209; 8817; 2335; 11167; 2517; 2527; 51343; 27165; 2885; 3488; 25959; 11155
**NK.Tcell Charoentong CellRep.2017 PMID.28052254**	**NK cells**	Signature	PMID.28052254	3802; 3809; 3812; 6696; 10385; 55704; 9398; 135228; 51267; 10695; 245812; 1385; 200186; 64784; 1437; 2015; 8061; 2526; 3308; 126393; 3384; 9235; 145501; 3698; 23081; 3804; 3811; 84623; 3821; 10219; 3920; 10990; 4277; 9437; 84901; 10630; 6386; 9056; 7056; 54209; 85480; 53347; 7412; 51087
**NK ImSig.Nirmal CancerImmunolRes. 2018 PMID.30266715**	**NK cells**	Signature	PMID.30266715	8302; 5551; 3822; 3823; 57292; 3810; 30009; 100132285; 3806; 3808; 154075; 115653; 3802; 3804; 117157; 3824; 3803; 3811; 3812; 3805
**NK.MCP Helmink Nature.2020 PMID.31942077**	**NK cells**	Signature	PMID.31942077	11126; 3802; 3804; 3805; 3811; 3813; 9437; 5729; 117157
**Bcells.Plasmablast Dybaer JCO.2015 PMID.25800755**	**Plasma cells**	Signature	PMID.25800755	639; 857; 999; 1534; 2081; 2487; 2549; 3493; 3537; 3655; 3662; 4121; 4189; 4695; 7421; 7494; 8339; 8343; 8344; 8346; 8347; 8349; 8970; 9236; 10788; 10802; 10970; 22862; 22936; 23446; 23710; 23766; 28396; 28831; 51237; 51303; 54502; 54855; 55132; 57823; 64764; 64778; 79694; 91319; 115908; 144535; 151556; 160428; 317649; 340542; 645784; 100533483; 101928615
**Plasma.cells CIBERSORT NatMethods.2015 PMID.25822800**	**Plasma cells**	Signature	PMID.25822800	973; 79368; 91316; 3112; 3495; 3507; 3514; 91353; 5026; 5368; 150094; 939; 160518; 2788; 80008; 608; 55840; 23457; 270; 51378; 6315; 79703; 2826; 952; 2848; 3012; 8339; 3497; 3738; 26251; 54923; 100130100; 4121; 79694; 22983; 374977; 51237; 5081; 5163; 25780; 5972; 6003; 642741; 6676;

© 2023 Fernandez-Martinez A et al. *JAMA Oncology.*

				27090; 9333; 7367; 81030; 7718
**Bcells.Plasma.cells. Metagene Miller GenomeBiol.2013 PMID.23618380**	**Plasma cells**	Signature	PMID.23618380	3537; 3500; 3507; 3493; 3492; 3514; 28831; 50802; 28823; 100293211; 5450; 608; 91316; 3495; 91353; 100423062
**Plasma.cells ImSig.Nirmal CancerImmunolRes. 2018 PMID.30266715**	**Plasma cells**	Signature	PMID.30266715	3492; 3502; 28823; 91353; 81567; 91316; 51237; 28902; 608; 28831; 3546; 3514; 3537
**STAT1 Rody BreastCancerResearch.2008 PMID.19272155**	**STAT**	Signature	PMID.19272155	6890; 2633; 64135; 5698; 4283; 3659; 6373; 3627; 3620; 6772
**Proliferation.Pathway ImSig.Nirmal CancerImmunolRes. 2018 PMID.30266715**	**T cells**	Signature	PMID.30266715	6491; 2146; 79733; 7272; 6790; 9212; 9232; 55388; 113115; 81620; 9134; 11004; 55143; 83879; 11339; 55215; 157313; 55789; 7153; 113130; 890; 259266; 83461; 9928; 983; 990; 3832; 51514; 56992; 22974; 51203; 84057; 55635; 55872; 4171; 11065; 1894; 4173; 5888; 4175; 29128; 4085; 6241; 11130; 554282; 6240; 701; 3925; 9833; 79801; 1163; 24137; 3149; 4751; 2305; 9768; 54443; 55165; 3161; 7083; 57405; 7298; 5427; 64151; 54892; 55355; 1058; 699; 26271; 220134; 55055; 10721; 9319; 9837; 51659; 91687; 10635; 4288; 51053; 9787; 83540; 146909; 1063; 991; 10403; 332; 1062; 29980; 1033; 29127; 10592; 891; 10733; 9133; 55010; 5111; 6941; 10112
**Immune.CTLA4.CXCL.FOXP3 TCGA.BRCA.1198 JCI.2020 PMID.32573490.**	**T cells**	Signature	PMID.32573490	1493; 6373; 3627; 4283; 3604; 50615; 56833; 50943; 3620; 94240; 2633
**CD8.Cluster Iglesia CCR.2014 PMID.24916698**	**T cells**	Signature	PMID.24916698	256380; 151888; 925; 3003; 29909; 128611; 5790; 374403; 10663; 4063; 9744; 27240; 201633; 3932; 55423; 919; 84174; 3702; 10225; 4068; 921; 916; 914; 915; 2833; 114836; 3561; 53347; 6504; 149628; 939; 50852;

© 2023 Fernandez-Martinez A et al. *JAMA Oncology.*

				3001; 30009; 5778; 6352; 4818; 5551; 962; 154075; 917; 7535; 22914; 283897; 923; 5579; 8698
**Tcells.CD8.MCP Petitprez Nature.2020 PMID.31942077**	**T cells**	Signature	PMID.31942077	926; 926; 926; 926
**Tcells.CD4.Memory. Activated CIBERSORT NatMethods.2015 PMID.25822800**	**T cells**	Signature	PMID.25822800	914; 919; 915; 917; 924; 1803; 29851; 3932; 4068; 28755; 50852; 53347; 959; 923; 29909; 4818; 940; 1493; 3002; 3902; 6364; 993; 1437; 10563; 2842; 3458; 3595; 3605; 55801; 3559; 3562; 3565; 3578; 4049; 4998; 5367; 9136; 220134; 7293; 79931
**Tcells.CD4.Memory. Resting CIBERSORT NatMethods.2015 PMID.25822800**	**T cells**	Signature	PMID.25822800	939; 54923; 642741; 9214; 4050; 64919; 914; 919; 915; 917; 924; 1803; 2323; 9402; 3004; 29851; 3575; 3702; 3932; 51176; 4063; 4068; 55423; 6932; 28755; 50852; 28671; 28639; 53347; 7535; 10235; 959; 969; 5790; 6352; 916; 923; 10225; 1521; 29909; 3001; 3003; 3820; 4818; 22821; 2848; 1235; 940; 920; 1493; 1606; 2035; 2113; 55336; 57326; 5732; 11123; 140801; 6489; 28670; 28662; 28680; 28677; 678
**Tcells.CD4.Naive CIBERSORT NatMethods.2015 PMID.25822800**	**T cells**	Signature	PMID.25822800	939; 54923; 642741; 9214; 4050; 64919; 914; 919; 915; 917; 924; 1803; 1823; 2323; 9402; 3004; 29851; 3575; 3702; 3932; 51176; 4063; 11184; 79884; 4068; 55423; 6932; 28755; 50852; 28671; 28639; 53347; 7535; 10235; 9744; 79722; 80162; 1236; 959; 55086; 2041; 79667; 79690; 2587; 2825; 27040; 5871; 26297; 50853; 7476; 7754; 25799
**Tcells.CD8 CIBERSORT NatMethods.2015 PMID.25822800**	**T cells**	Signature	PMID.25822800	3514; 939; 54923; 642741; 969; 9214; 4050; 5790; 64919; 6352; 914; 919; 915; 916; 917; 923; 924; 925; 926; 10225; 56253; 8530; 1521; 1803; 1823; 1844; 2323; 10578; 29909;

© 2023 Fernandez-Martinez A et al. *JAMA Oncology.*

				9402; 3001; 3002; 2999; 3003; 3004; 29851; 3575; 3702; 3820; 3823; 8302; 3824; 51348; 22914; 3902; 3932; 51176; 4063; 11184; 79884; 259197; 4818; 113791; 5551; 5729; 79037; 22821; 4068; 55423; 6932; 28755; 50852; 28673; 28671; 28639; 28526; 53347; 7535
**Tcells.Follicular.Helper CIBERSORT NatMethods.2015 PMID.25822800**	**T cells**	Signature	PMID.25822800	914; 919; 915; 917; 924; 29851; 3932; 4068; 28755; 50852; 53347; 959; 1493; 3902; 10563; 2842; 4049; 7293; 939; 642741; 9214; 64919; 3004; 3702; 51176; 55423; 6932; 28671; 28639; 7535; 969; 5790; 3820; 1606; 6489; 28680; 28677; 11184; 79884; 80162; 27040; 79037; 150094; 643; 767; 1117; 2354; 7976; 3382; 59067; 23178; 5133; 5996; 56301; 28951; 7253; 65986
**Tcells.Gamma.Delta CIBERSORT NatMethods.2015 PMID.25822800**	**T cells**	Signature	PMID.25822800	914; 919; 915; 917; 3932; 4068; 53347; 3004; 55423; 28639; 11184; 29909; 27240; 3560; 8631; 3902; 6932; 3820; 27040; 79037; 3002; 4063; 6352; 3001; 3003; 925; 926; 8530; 1844; 10578; 2999; 3824; 22914; 5551; 5729; 28526; 2841; 631; 9024; 1234; 11126; 51744; 11314; 1010; 8292; 10663; 2672; 2996; 8807; 10219; 442236; 3973; 4110; 26030; 6335; 643503; 8875; 79973
**Tcells.Regulatory.Tregs CIBERSORT NatMethods.2015 PMID.25822800**	**T cells**	Signature	PMID.25822800	914; 919; 915; 917; 29851; 3932; 4068; 28755; 50852; 53347; 1493; 2842; 939; 64919; 3004; 3702; 55423; 28639; 7535; 1606; 28677; 11184; 1803; 923; 29909; 940; 3559; 5367; 4050; 916; 10225; 920; 11123; 2825; 4153; 27240; 8538; 921; 970; 752014; 29121; 1946; 50943; 83786; 3090; 203510; 3560; 55243; 3904; 23547; 126987; 4861; 4917; 56143; 9651;

© 2023 Fernandez-Martinez A et al. *JAMA Oncology.*

				5739; 5793; 6261; 25956; 5413; 8631; 9806; 6756; 7299
**Cytolytic.Activity Rooney Cell.2015 PMID.25594174**	**T cells**	Signature	PMID.25594174	3001; 5551
**Cytotoxic.Lymphocytes.MCP Petitprez Nature.2020 PMID.31942077**	**T cells**	Signature	PMID.31942077	925; 8320; 83888; 10578; 3823; 8302; 3824
**Immune.Active Hollern Cell.2019 PMID.31730857**	**T cells**	Signature	PMID.31730857	12; 356; 433; 640; 718; 914; 915; 916; 917; 920; 923; 924; 925; 927; 930; 931; 933; 939; 940; 959; 969; 972; 973; 1233; 1380; 1493; 1776; 2634; 2841; 2864; 3002; 3109; 3111; 3112; 3117; 3120; 3135; 3274; 3363; 3458; 3495; 3500; 3502; 3512; 3514; 3559; 3560; 3581; 3593; 3595; 3627; 3682; 3702; 3816; 3820; 3824; 4049; 4050; 4261; 4283; 4332; 4818; 5079; 5133; 5551; 5790; 6039; 6346; 6352; 6363; 6367; 6373; 6434; 6504; 6518; 6773; 6846; 6909; 8302; 8530; 8740; 8798; 8942; 9402; 9476; 9934; 10019; 10100; 10225; 10420; 10563; 10612; 10663; 10666; 10803; 22806; 22914; 26191; 26279; 27334; 28557; 28558; 28621; 28683; 28776; 29126; 29851; 29909; 51617; 53347; 54929; 56253; 56949; 57823; 64386; 80380; 84327; 84636; 114132; 114770; 114836; 115350; 115362; 117157; 137970; 152789; 158067; 163351; 168537; 202309; 221150; 221830; 246778; 283420; 284486; 345611; 353345; 387914; 387923; 388507; 388646; 400410; 445347; 100423062; 100527949; 84824
**CTLA4.Pathway GSEA.BIOCARTA ProcNatlAcadSciUSA.2005 PMID.16199517**	**T cells**	Signature	PMID.16199517	5781; 941; 942; 940; 915; 916; 917; 3702; 5290; 23308; 6957; 6955; 2885; 919; 29851; 5295; 3932; 3123; 3122; 1493; 3558

© 2023 Fernandez-Martinez A et al. *JAMA Oncology.*

**Immune.cell.Cluster.PerouLab Fan.GSEA.GP2 BMCMedGenomics.2011 PMID.21214954**	**T cells**	Signature	PMID.16199517	7453; 3620; 6039; 8477; 4332; 27074; 25816; 4067; 7128; 51316; 3575; 1439; 3659; 7133; 27299; 56833; 55423; 4050; 5790; 1236; 9806; 5341; 972; 3108; 3122; 3109; 2207; 7305; 7805; 3689; 712; 6772; 6373; 3627; 2633; 6890; 5698; 55303; 5552; 10875; 3059; 9938; 3932; 2124; 55843; 9595; 3001; 3937; 963; 3587; 962; 6352; 915; 914; 3003; 5788; 4283; 9535; 3394; 1520; 22797; 6503; 9056; 7940; 199; 942; 3071; 9473; 4069; 1043; 6402; 3134; 3106; 3133; 3002; 10507; 11118; 10384; 834
**Tcells.Bcell.KEGG.hematopoietic.cell.lineage GSEA.GP2 ProcNatlAcadSciUSA.2005 PMID.16199517**	**T cells**	Signature	PMID.16199517	2811; 2812; 2056; 3581; 945; 7124; 2815; 3684; 947; 948; 2814; 3676; 3675; 4254; 3674; 2209; 3678; 2208; 2993; 7066; 7850; 3575; 4311; 3590; 930; 931; 7037; 933; 3815; 2322; 3690; 2323; 3552; 1380; 929; 1378; 7173; 2057; 3589; 1437; 3126; 920; 1436; 3127; 1435; 3125; 3563; 3562; 966; 652799; 928; 925; 926; 960; 924; 921; 1438; 1604; 1791; 1440; 3553; 3554; 3559; 3655; 3570; 3673; 3672; 1441; 3574; 951; 914; 915; 916; 952; 917; 910; 290; 911; 912; 913; 909; 3565; 3566; 3567; 3568; 3569; 3123; 3122
**PD1.Signaling.Reactome GSEA ProcNatlAcadSciUSA.2005 PMID.16199517**	**T cells**	Signature	PMID.16199517	1445; 29126; 3113; 3115; 3117; 3118; 3123; 3125; 3127; 3932; 5133; 5777; 80380; 915; 916; 917; 919; 920
**Tcells.CD8 Bindea Immunity.2013 PMID.24138885**	**T cells**	Signature	PMID.24138885	166; 321; 678; 687; 819; 925; 926; 1647; 1831; 2323; 3004; 3337; 5196; 5504; 5551; 5935; 6432; 6844; 6903; 6935; 7536; 7570; 7644; 7994; 9194; 11322; 23060; 23484; 29777; 51275; 54923; 55602; 55615; 55623; 79441; 91304

© 2023 Fernandez-Martinez A et al. *JAMA Oncology.*

**Cytotoxic.cells Bindea Immunity.2013 PMID.24138885**	**T cells**	Signature	PMID.24138885	321; 864; 1521; 1844; 2999; 3001; 3820; 3824; 4818; 6095; 7704; 10578; 22914; 51348; 59307; 80833; 339005
**Tcells Bindea Immunity.2013 PMID.24138885**	**T cells**	Signature	PMID.24138885	914; 915; 916; 917; 923; 940; 3932; 4068; 5588; 6955; 8631; 9452; 10225; 28639; 28755; 50852; 55340; 64919; 83988
**Tcells.Thelper Bindea Immunity.2013 PMID.24138885**	**T cells**	Signature	PMID.24138885	940; 987; 1386; 4676; 5527; 6117; 6955; 7332; 8604; 9452; 9632; 10538; 10541; 10730; 10772; 23015; 25842; 29851; 55274; 57122; 63901; 79009; 79866; 285527
**Tcells.Follicular.Helper Bindea Immunity.2013 PMID.24138885**	**T cells**	Signature	PMID.24138885	643; 753; 1114; 1117; 3382; 4086; 4094; 5783; 6489; 7253; 8362; 8645; 8851; 9760; 10563; 10585; 23178; 23462; 27087; 27347; 54436; 55423; 56301; 57496; 57535; 63892; 92595; 28986; 4646; 4647; 5133; 5816
**Tcells.Gamma.Delta Bindea Immunity.2013 PMID.24138885**	**T cells**	Signature	PMID.24138885	6964; 6983; 9638; 10485; 11126; 445347
**Tcells.Th1.cells Bindea Immunity.2013 PMID.24138885**	**T cells**	Signature	PMID.24138885	323; 347; 684; 952; 970; 1437; 1493; 1803; 1839; 1847; 2678; 3458; 3595; 4049; 6351; 6443; 7804; 8418; 9143; 9162; 10079; 10950; 25975; 27033; 50616; 54674; 55816
**Tcells.Th17.cells Bindea Immunity.2013 PMID.24138885**	**T cells**	Signature	PMID.24138885	3605; 6097; 23765
**Tcells.Th2.cells Bindea Immunity.2013 PMID.24138885**	**T cells**	Signature	PMID.24138885	107; 286; 332; 995; 1063; 1719; 2625; 2941; 3070; 3904; 4087; 4151; 5251; 5367; 5740; 6632; 7813; 8317; 9645; 10663; 11169; 23516; 51474; 54806; 55247; 55801; 728210
**Immune.Hot.vs.Cold.CD8 Cabrita Nature.2020 PMID.31942071**	**T cells**	Signature	PMID.31942071	4069; 5552; 962; 51316; 1075; 9595; 939; 3123; 3394; 2533; 2124; 5880; 6352; 3122; 3119; 11151; 3512; 91353; 951; 3575; 6039; 4283; 3117; 27299; 963; 7805; 2207; 7852;

© 2023 Fernandez-Martinez A et al. *JAMA Oncology.*

				3689; 3059; 2123; 1520; 5341; 64092; 6355; 713; 3587; 51816; 7351; 3115; 10875; 712; 2745; 51338; 241; 11040; 3001; 3003; 915; 3669; 9938; 969; 1043; 914; 3702; 6402; 11184; 1236; 27074; 1880; 9214; 26191; 50856; 4818; 64919; 6363; 9535; 3071; 3683; 2313; 925; 5551; 60489; 474344; 942; 8477; 9262; 10288; 91543; 6890; 3135; 3620; 5698; 3134; 3133; 3106; 9246; 6772; 3429; 5996; 8519; 5696; 3437; 10125; 9636; 6891; 4599; 10964; 55601; 4939; 3600; 54739; 2537; 3659; 3458; 1776; 7412; 4050; 3002; 973; 972; 10563; 4067; 330; 25816; 5734; 729230; 6387; 51237; 3113; 64231; 4689; 7133; 6362; 3109; 10437; 56833; 3128; 4688; 6348; 7941; 9332; 6648; 7453; 3126; 8942; 7128; 7305; 3108; 1436; 629; 240; 3936; 1512; 1536; 5265; 54440; 3055; 2219; 1675; 30835; 54855; 952; 1794; 4033; 5579; 80342; 3957; 7940; 199; 11262; 695; 55340; 716; 730; 1438; 919; 917; 4068; 10225; 29851; 3112; 3820; 3695; 26051; 120; 924; 6366; 8875; 5790; 834; 971; 4066; 11025; 10791; 55303; 23228; 6414; 81030; 50619; 387751; 54923; 3627; 9235; 6373; 10578; 9404; 2209; 9056; 7462; 6775; 11006; 9447; 2359; 2833; 2999; 8685; 6357; 5721; 10346; 5920; 80833; 10261; 51056; 79961; 54518; 83593; 80709; 5777; 714; 6001; 10870; 58475; 51296; 257106; 5294; 26157; 10320; 219285; 64098; 441168; 115362; 256236; 259307; 30061; 10507; 81704; 353514; 7318; 10068;

© 2023 Fernandez-Martinez A et al. *JAMA Oncology.*

				3433; 255231; 9619; 3635; 9450; 27071; 57823; 118788; 84433; 5336; 7454; 114836; 10537; 4938; 10148; 64407; 8638; 2643; 94240; 10538; 51225; 51284; 4973; 27128; 113791; 53829; 6688; 84803; 203328; 11309; 374403; 5583; 92241; 54900; 11320; 100131187; 286530; 64135; 3430; 3665; 717; 9476; 3107; 3125; 3120; 2210; 3824; 3902; 28874; 28896; 3111; 3904; 3805; 146722; 89790; 10990; 26270; 8743; 83666; 168537; 217; 3560; 2268; 149628; 51311; 2517; 54502; 7538; 64005; 128346; 3136; 115361; 414062; 64926; 8676; 79895; 160364; 3543; 4242; 56990; 10170; 170575; 10866; 27334; 2203; 114614; 160365; 89846; 118932; 51703; 154; 202309; 57552; 155038; 55840; 9050; 221472; 9560; 1318; 1050; 9806; 147463; 126306; 84969; 51755; 654817; 10800; 260436; 115352; 57172; 430; 376412; 387841; 400759; 79037
**Immune.87 Perez JCO.2015 PMID.2560586**	**T cells**	Signature	PMID.2560586	3635; 729230; 3575; 3133; 3689; 3487; 7454; 10870; 3071; 7494; 81793; 6352; 7133; 3385; 3821; 4049; 3458; 4050; 959; 3394; 7412; 5729; 5734; 941; 4068; 54106; 973; 2833; 313; 695; 9934; 120425; 1235; 4069; 4065; 7852; 3683; 915; 5788; 976; 3561; 3766; 3932; 913; 2534; 11126; 6693; 5336; 3115; 3823; 9437; 3620; 916; 26191; 3112; 1233; 2826; 919; 10563; 84174; 3001; 718; 5168; 6932; 917; 6387; 10225; 54900; 974; 29126; 952; 5551; 1493; 84632; 5588; 80833; 1236; 29851; 6402; 10219;

© 2023 Fernandez-Martinez A et al. *JAMA Oncology.*

				3543; 3702; 925; 115650; 6366; 6403; 6363
**LCK Rody BreastCancerResearch.2008 PMID.19272155**	**T cells**	Signature	PMID.19272155	55843; 22914; 54440; 9938; 6504; 55340; 4068; 1236; 3702; 28568; 5880; 11151; 5293; 9404; 5579; 6775; 9535; 6404; 10875; 2124; 1439; 3003; 64092; 55303; 3394; 3071; 3587; 28755; 3635; 51316; 6352; 919; 4050; 3561; 3059; 3575; 963; 1231; 3932; 915; 939; 3001; 6402; 28566; 914; 962; 6503; 3937; 5552; 5788
**MCD3.CD8 Fan BMCMedGenomics.2011 PMID.21214954**	**T cells**	Signature	PMID.21214954	3932; 390243; 151888; 5450; 4242; 115350; 84824; 9214; 51617; 1840; 940; 26999; 4068; 3111; 921; 9840; 27040; 89857; 2833; 4050; 84329; 6689; 1380; 30009; 10750; 10870; 3603; 643; 974; 51237; 931; 55843; 26228; 923; 8631; 926; 915; 914; 3702; 925; 917; 6402; 399; 3695; 3560; 1731; 27334; 939; 6775; 80342; 26279; 393; 26191; 9476; 1269; 168537; 84433
**MERCK.Immune.Signature Cristescu Science.2018 PMID.30309915**	**T cells**	Signature	PMID.30309915	6352; 939; 29126; 80381; 925; 1240; 4283; 10663; 3117; 3123; 3133; 3620; 3902; 4818; 80380; 5699; 6772; 201633
**Tcells.NK.Metagene Miller GenomeBiol.2013 PMID.23618380**	**T cells**	Signature	PMID.23618380	4242; 10875; 3587; 916; 5579; 2841; 2124; 1521; 3820; 6375; 3059; 3635; 5293; 962; 951; 6402; 3932; 54440; 1439; 939; 3003; 729230; 11151; 3603; 28517; 3702; 10730; 5788; 5880; 4818; 55340; 55843; 474344; 9938
**Tcells.ImmuneProfiles.Mouse.Human Shay PNAS.2013 PMID.23382184**	**T cells**	Signature	PMID.23382184	23; 120; 158; 166; 360; 399; 406; 472; 476; 596; 814; 841; 894; 914; 915; 916; 917; 919; 921; 923; 939; 940; 944; 955; 1107; 1236; 1455; 1477; 1493; 1540; 1606; 1643; 1778; 1936; 2071; 2073; 2113; 2145; 2308; 2534; 2580; 2617; 2625; 3097; 3268; 3337; 3384; 3396; 3561; 3572; 3575; 3603; 3655;

© 2023 Fernandez-Martinez A et al. *JAMA Oncology.*

				3661; 3695; 3702; 3707; 3738; 3932; 3985; 4063; 4068; 4216; 4242; 4552; 4676; 4775; 4798; 4839; 4891; 4926; 5001; 5140; 5144; 5293; 5297; 5335; 5527; 5528; 5578; 5583; 5588; 5727; 5965; 6095; 6117; 6197; 6232; 6301; 6304; 6415; 6448; 6489; 6504; 6609; 6709; 6772; 6777; 6829; 6932; 7049; 7174; 7277; 7294; 7433; 7535; 7597; 7841; 8216; 8445; 8449; 8480; 8514; 8525; 8631; 8651; 8718; 8720; 8821; 8871; 8888; 8890; 8893; 8906; 9047; 9057; 9267; 9368; 9402; 9452; 9730; 9903; 9913; 9919; 9923; 10048; 10123; 10125; 10225; 10235; 10291; 10531; 10641; 10666; 10671; 10723; 10767; 10801; 10868; 10947; 10985; 11059; 11123; 11322; 11329; 11336; 22859; 22887; 22890; 22993; 23033; 23046; 23214; 23215; 23248; 23338; 23344; 23348; 23352; 23368; 23370; 23381; 23484; 23492; 23523; 23526; 23774; 24148; 25777; 25948; 25996; 26009; 26018; 26119; 26207; 26249; 27040; 27240; 27347; 28951; 28984; 29851; 29980; 50852; 51070; 51174; 51176; 51185; 51367; 51466; 51531; 51586; 51654; 51669; 51696; 51735; 51761; 53347; 54093; 54509; 54541; 54606; 54870; 54941; 55037; 55049; 55080; 55102; 55127; 55253; 55275; 55303; 55324; 55623; 55667; 55695; 55703; 55795; 56829; 56948; 56965; 57062; 57185; 57194; 57198; 59307; 60487; 63892; 64132; 64210; 64425; 64601;

© 2023 Fernandez-Martinez A et al. *JAMA Oncology.*

				64745; 64750; 64784; 64919; 65083; 65983; 79573; 79595; 79695; 79726; 79913; 79918; 79961; 79991; 80007; 80152; 80198; 80335; 80342; 80762; 81606; 84162; 84859; 89941; 93210; 113791; 117246; 120526; 399665; 474344; 1039; 23020; 50649; 55718; 55843; 6687; 7067; 7267; 9138; 926; 9906; 101060399; 101929240; 101930241; 101060521; 101928361; 101930112; 9572; 286495; 100505585; 100996919; 728661
**Tcells.CD4.Activated Charoentong CellRep.2017 PMID.28052254**	**T cells**	Signature	PMID.28052254	83990; 9447; 330; 6364; 6351; 6352; 891; 1236; 1844; 157570; 2113; 9156; 54536; 3376; 3702; 3832; 9735; 83540; 9055; 29968; 5996; 219790; 64092; 6402; 50852
**Tcells.CD8.Activated Charoentong CellRep.2017 PMID.28052254**	**T cells**	Signature	PMID.28052254	917; 11047; 10598; 29071; 10693; 951; 915; 916; 969; 925; 1070; 1434; 79833; 10578; 84706; 3001; 2999; 3003; 3560; 3932; 9019; 4818; 113791; 51651; 26517; 7535
**Tcells.CD4.Central.Memory Charoentong CellRep.2017 PMID.28052254**	**T cells**	Signature	PMID.28052254	2937; 171586; 79026; 304; 360; 80162; 648; 28969; 967; 1282; 1540; 63916; 2534; 11010; 10581; 3688; 3689; 688; 4046; 4715; 5315; 81855; 55423; 4089; 6810; 8717; 7431; 2547
**Tcells.CD8.Central.Memory Charoentong CellRep.2017 PMID.28052254**	**T cells**	Signature	PMID.28052254	2288; 81; 8038; 115; 2162; 2207; 2215; 2252; 2746; 2760; 2990; 3557; 25926; 4914; 5914; 79589; 6614; 8792; 9878; 7311; 80329
**Tcells.CD4.Effector.Memory Charoentong CellRep.2017 PMID.28052254**	**T cells**	Signature	PMID.28052254	7857; 472; 836; 844; 342510; 1615; 23348; 5393; 2146; 51573; 146433; 8031; 4747; 5157; 5742; 85021; 8436; 100049587; 946; 6886; 22797; 54962; 27010; 7381; 8287; 7456; 85437
**Tcells.CD8.Effector.Memory Charoentong**	**T cells**	Signature	PMID.28052254	80833; 79658; 9744; 57205; 719; 1234; 11126; 1604; 8837; 1240; 27071; 343413; 2323; 3004;

© 2023 Fernandez-Martinez A et al. *JAMA Oncology.*

**CellRep.2017 PMID.28052254**				145864; 3109; 3113; 3115; 3428; 54923; 4058; 4792; 80854; 150094; 28951
**Tcells.Gamma.Delta Charoentong CellRep.2017 PMID.28052254**	**T cells**	Signature	PMID.28052254	2171; 54; 366; 11118; 79630; 22900; 6357; 6362; 30835; 945; 948; 1020; 28988; 2168; 8772; 3588; 55975; 51348; 144501; 3915; 254251; 3956; 4001; 5598; 4213; 9848; 9562; 4352; 6229; 6201; 6203
**Tcells.Regulatory Charoentong CellRep.2017 PMID.28052254**	**T cells**	Signature	PMID.28052254	3988; 4035; 6349; 971; 23601; 50943; 3676; 3897; 115353; 8685; 4321; 4332; 4360; 64231; 53918; 5341; 11098; 5739; 7903; 23166
**Tcells.Follicular.Helper Charoentong CellRep.2017 PMID.28052254**	**T cells**	Signature	PMID.28052254	2247; 8829; 8480; 27087; 604; 4345; 9308; 8832; 8851; 1050; 51816; 55501; 10462; 50856; 9022; 1436; 1520; 23336; 1803; 2841; 10625; 3803; 5641; 2615; 4161; 100507436; 4684; 9436; 5133; 80380; 10016; 5052; 135250; 27036; 27180; 7301
**Tcells.Th1.cells Charoentong CellRep.2017 PMID.28052254**	**T cells**	Signature	PMID.28052254	1600; 84085; 3357; 6403; 27240; 101; 23382; 214; 8706; 166379; 683; 800; 977; 961; 962; 1043; 963; 966; 923; 968; 924; 970; 10225; 976; 10878; 1131; 64581; 91522; 1286; 50509; 220107; 8447; 11072; 2012; 8507; 2161; 54463; 9103; 5045; 139716; 2628; 9945; 2848; 64388; 26762; 3290; 30811; 3385; 3481; 3592; 3659; 3691; 4056; 11253; 4190; 284207; 23531; 55034; 5026; 92241; 6004; 6261; 60485; 6401; 30011; 89790; 8631; 51000; 6769; 30009; 80745; 201633; 51311; 7132; 7185; 10758; 9839
**Tcells.Th17.cells Charoentong CellRep.2017 PMID.28052254**	**T cells**	Signature	PMID.28052254	10803; 1084; 19; 5243; 81792; 286; 118932; 8707; 145741; 388125; 760; 23261; 85478; 958; 11251; 57560; 3605; 27189; 112744; 23765; 84818; 132014; 51561; 286676; 79836; 284948; 79155

© 2023 Fernandez-Martinez A et al. *JAMA Oncology.*

**Tcells.Th2 Charoentong CellRep.2017 PMID.28052254**	T cells	Signature	PMID.28052254	10395; 8828; 64699; 51676; 332; 995; 8317; 1063; 1466; 10663; 1612; 1719; 56521; 1848; 7813; 2625; 2770; 2941; 3070; 55801; 3904; 27074; 114876; 5142; 22822; 5321; 5874; 27303; 54941
**Tcells ImSig.Nirmal CancerImmunolRes. 2018 PMID.30266715**	T cells	Signature	PMID.30266715	9595; 3603; 925; 9450; 6967; 1043; 79037; 84329; 55843; 128346; 695; 6503; 962; 10225; 4068; 197358; 89857; 2313; 28755; 2123; 139716; 10663; 2124; 55619; 64174; 399; 64333; 3820; 64098; 56253; 917; 915; 916; 2841; 29909; 6983; 5583; 50852; 11262; 9938; 10870; 3604; 3003; 1236; 55423; 445347; 51411; 3936; 3683; 4283; 654816; 5790; 3575; 54518; 1794; 51561; 3587; 29851; 9051; 914; 53347; 923; 939; 92241; 940; 2533; 26157; 3702; 55303; 374403; 55340; 474344; 168537; 6363; 64407; 81704; 83593; 11151; 2534; 9535; 80342; 54440; 441168
**Tcells.NK.51genes Miller GenomeBiol.2013 PMID.23618380**	T cells	Signature	PMID.23618380	55843; 9938; 729230; 1236; 919; 939; 951; 915; 916; 962; 11151; 1439; 1521; 2124; 10875; 2533; 55340; 474344; 9535; 2841; 3001; 3003; 3059; 3133; 3587; 3603; 51561; 3635; 3702; 3820; 3932; 9404; 4050; 4242; 4818; 5293; 5579; 5788; 5790; 79037; 5880; 54440; 6402; 4068; 7128; 6846; 10730; 57175; 6375; 100527949; 10734
**Tcells.CD8.Effector.vs.Naive.2 Pauken Science.2016 PMID.27789795**	T cells	Signature	PMID.27789795	54443; 378708; 9824; 84986; 55723; 259266; 79915; 6790; 9212; 332; 672; 83990; 699; 701; 57082; 836; 890; 891; 9133; 899; 991; 995; 990; 157313; 83461; 113130; 55143; 1033; 1058; 1062; 1063; 64946; 64105; 55839; 401541; 55165; 1111; 26586; 150468;

© 2023 Fernandez-Martinez A et al. *JAMA Oncology.*

				1163; 1164; 63967; 55635; 1719; 81624; 9787; 79075; 51514; 79733; 1894; 157570; 9700; 9156; 2146; 2171; 26271; 2237; 63979; 2305; 9837; 3014; 3070; 3161; 3619; 9768; 57650; 3832; 56992; 146909; 10112; 3835; 9493; 11004; 24137; 9735; 3838; 3978; 4001; 4085; 55388; 4172; 4174; 4176; 9833; 4288; 4603; 9918; 64151; 54892; 23397; 10403; 55247; 4751; 83540; 51203; 55872; 5347; 10733; 5422; 5426; 9055; 5557; 55771; 22949; 29127; 5888; 10635; 8438; 5932; 5983; 6240; 6241; 151648; 79801; 29015; 10592; 10615; 147841; 57405; 6491; 3925; 256126; 10460; 6941; 7153; 22974; 9319; 7272; 7298; 11065; 29128; 7468; 55055
**Tcells.CD8.Exhausted.vs.AntiPDL1.2 Pauken Science.2016 PMID.27789795**	**T cells**	Signature	PMID.27789795	132; 79026; 50808; 302; 302; 307; 259266; 332; 699; 701; 11238; 837; 890; 891; 899; 729230; 1234; 1235; 968; 941; 991; 994; 995; 990; 983; 1062; 55165; 1111; 11113; 26586; 1163; 1164; 1240; 1397; 2833; 1595; 23604; 10926; 1718; 1719; 115817; 81624; 9787; 1763; 80005; 11034; 79733; 1894; 133418; 2012; 2034; 54206; 11082; 116496; 2274; 63979; 2678; 2745; 2769; 10007; 3005; 3014; 55363; 3161; 51617; 8809; 7850; 3575; 10788; 3687; 3688; 9768; 57650; 23095; 10112; 3835; 9493; 10219; 3914; 3956; 3978; 9516; 4001; 122769; 4171; 4172; 255231; 4288; 84057; 51264; 4603; 4605; 4644; 9918; 64151; 54892; 10787; 10403; 55247; 4751; 83540; 51203; 55872; 10733; 5422; 5426; 5427; 5441; 9055; 5557;

© 2023 Fernandez-Martinez A et al. *JAMA Oncology.*

				22949; 8438; 54502; 5979; 23433; 23433; 6094; 6095; 6281; 6275; 6275; 10505; 220134; 28232; 6652; 10615; 6713; 6491; 10460; 6941; 7153; 22974; 9697; 6302; 125488; 145567; 11065; 137886; 29128; 9094; 51201
**Tcells.CD8.Exhausted.vs.Naive.2 Pauken Science.2016 PMID.27789795**	**T cells**	Signature	PMID.27789795	33; 54443; 9824; 84986; 55723; 259266; 79915; 580; 332; 83990; 699; 701; 57082; 836; 890; 891; 9133; 9134; 991; 995; 990; 157313; 83461; 113130; 55143; 1031; 1033; 1058; 1062; 1063; 64946; 64105; 401541; 55165; 1111; 26586; 150468; 1163; 1164; 63967; 55635; 1719; 81624; 9787; 79075; 51514; 1854; 79733; 1894; 30844; 54821; 157570; 9156; 2146; 2171; 26271; 2237; 63979; 2305; 9837; 3014; 3070; 3148; 3161; 7850; 3619; 3662; 9768; 57650; 3832; 56992; 3835; 9493; 24137; 9735; 3902; 3978; 4001; 4085; 9833; 4288; 4603; 4635; 64151; 54892; 23397; 10403; 55247; 4751; 83540; 51203; 132299; 55872; 5133; 5347; 10733; 5352; 5422; 5426; 9055; 5557; 55771; 5888; 10635; 5932; 5983; 79621; 6240; 6241; 151648; 79801; 10592; 10615; 147841; 57405; 6491; 3925; 256126; 10460; 6941; 22974; 9319; 7272; 7298; 11065; 7468; 6375
**Tcells.CD8.Memory.vs.Naive.1 Pauken Science.2016 PMID.27789795**	**T cells**	Signature	PMID.27789795	5243; 11332; 302; 8553; 55450; 822; 824; 834; 836; 837; 6352; 729230; 1234; 11126; 1154; 56253; 8530; 1493; 2833; 23191; 23258; 8411; 79180; 2012; 5167; 2123; 356; 2207; 10875; 115361; 2745; 9446; 3001; 3003; 3099; 84525; 3398; 10437; 3458; 3587; 8809; 9173; 10219; 3956; 3958; 9516; 56925; 23643;

© 2023 Fernandez-Martinez A et al. *JAMA Oncology.*

				256586; 29969; 8829; 4953; 8993; 123; 5359; 9055; 10549; 23433; 6281; 6275; 6277; 5269; 9047; 8935; 6645; 10402; 140738; 8784; 7133; 58485; 6302; 7305; 9094
**Tcells.Activation Petitprez Nature.2020 PMID.31942077**	T cells	Signature	PMID.31942077	4283; 3627; 3458; 3600; 58191
**Tcells.Cluster Iglesia CCR.2014 PMID.24916698**	T cells	Signature	PMID.24916698	100188949; 8530; 9806; 2672; 497189; 140947; 5133; 11184; 9051; 11151; 11262; 115352; 29851; 1493; 4283; 56253; 9840; 729230; 959; 952; 57823; 3662; 54900; 387751; 84636; 256380; 151888; 925; 3003; 29909; 128611; 5790; 374403; 10663; 4063; 9744; 27240; 201633; 3932; 55423; 919; 84174; 3702; 10225; 4068; 921; 916; 914; 915; 2833; 114836; 3561; 53347; 6504; 149628; 939; 50852; 3001; 30009; 5778; 6352; 4818; 5551; 962; 154075; 917; 7535; 22914; 283897; 923; 5579; 8698; 8320; 387357; 356; 120425; 3718; 26279; 4049; 924; 1043; 3004; 27040
**Tcells.Survival.2gene Petitprez Nature.2020 PMID.31942077**	T cells	Signature	PMID.31942077	939; 970
**Tcells.Regulatory.cell.2gene Petitprez Nature.2020 PMID.31942077**	T cells	Signature	PMID.31942077	50943; 8784
**Tcells.Resident.Memory.Single.cell Savas NatMed.2018 PMID.29942092**	T cells	Signature	PMID.29942092	199; 356; 444; 478; 486; 925; 926; 928; 952; 953; 967; 1124; 1130; 1230; 1234; 1521; 1627; 2115; 2171; 2494; 2530; 2589; 2590; 2793; 2820; 2975; 2999; 3001; 3002; 3117; 3119; 3122; 3123; 3127; 3162; 3458; 3672; 3673; 3695; 3805; 3821; 3822; 3824; 3887; 3892; 3902; 3984; 4647; 4681; 4753; 4818; 4929; 5133; 5175; 5445; 5446; 5551; 5763;

© 2023 Fernandez-Martinez A et al. *JAMA Oncology.*

				5921; 6003; 6348; 6351; 6983; 7273; 7292; 7412; 8110; 8214; 8302; 8320; 8395; 8441; 8482; 8538; 8543; 8572; 8611; 8848; 8875; 8903; 9022; 9242; 9289; 9495; 9523; 9560; 9576; 9666; 9760; 9788; 9901; 9976; 10253; 10447; 10510; 10529; 10578; 10839; 10863; 10962; 11098; 11145; 11182; 22914; 23604; 23665; 26228; 27333; 28531; 29083; 29988; 51225; 51232; 51655; 51744; 54072; 54438; 54674; 55084; 55501; 55653; 55784; 56253; 57121; 57196; 57406; 57493; 59342; 59345; 64127; 64218; 79070; 79895; 79899; 79931; 80119; 81553; 81563; 81618; 83903; 84056; 84329; 84632; 84868; 85302; 85379; 85458; 89987; 91937; 114614; 116841; 118788; 123355; 131450; 132332; 138948; 140739; 150763; 152100; 160335; 160365; 164668; 165186; 219931; 221184; 221188; 253782; 254559; 284406; 285025; 375775; 387923; 401124; 414062; 644150; 731247; 100287298; 100290966; 100505576; 100506776; 101927931
**Tcells.MCP Petitprez Nature.2020 PMID.31942077**	**T cells**	Signature	PMID.31942077	940; 915; 917; 921; 923; 100506915; 1493; 2323; 29851; 4118; 348035; 80714; 55423; 387357; 8718; 50852
**Tcells.CD8.Exhausted.Anti.PDL1.vs.Control.Metagene.1 Pauken Science.2016 PMID.27789795**	**T cells**	Signature	PMID.27789795	65987; 2766; 3428; 7444; 55803; 84875; 27074; 6672; 25865; 8775; 51296; 127544; 355; 715; 7318; 6373; 1267; 116071; 3601; 55013; 10865; 84930; 201176; 94240; 10135; 29126; 3659; 57169; 83666; 54625; 129607; 115361; 4283; 941; 57162; 85463; 9753; 51365; 256586; 26750; 3574;

© 2023 Fernandez-Martinez A et al. *JAMA Oncology.*

				79370; 602; 834; 64853; 388646; 1616; 163351; 146857; 345611; 7098; 25939; 2634; 55905; 4084; 5168; 51284; 56937; 4343; 3959; 55281; 81030; 3627; 79132; 64108; 6772; 64761; 684; 3669; 6737; 3433; 3430; 10379; 3434; 5610; 4938; 103; 91543; 4599; 4940; 10561; 8638; 4600; 55601; 3665; 6773; 4939; 11274; 3594; 8375; 8374
**Tcells.CD8.Exhausted.at.day.8.post.Imm.vs.Naive.Metagene.1 Pauken Science.2016 PMID.27789795**	**T cells**	Signature	PMID.27789795	967; 4635; 5355; 411; 84868; 56925; 952; 158219; 5359; 8411; 54502; 10549; 2745; 840; 3559; 824; 79180; 4783; 22822; 5734; 1326; 6351; 5341; 57823; 84419; 197259; 23710; 1493; 307; 196383; 5996; 214; 50515; 125488; 26031; 116496; 6310; 493; 201633; 8836; 2012; 5272; 11034; 29887; 639; 2591; 23692; 10809; 301; 6095; 195; 3005; 8993; 11332; 3915; 22926; 23589; 11163; 7905; 5911; 2669; 202; 55794; 10930; 8347; 11172; 83988; 8524; 493869; 11082; 1524; 9839; 53637; 4644; 3092; 3684; 1509; 3683; 4542; 3398; 960; 81615; 54843; 51201; 3595; 118788; 54476; 79158; 27293; 2151; 3903; 6118; 5795; 93663; 3672; 10663; 22837; 57105; 22856; 2081; 10402; 115361; 597; 2149; 54206; 356; 6352; 1234; 8553; 8829; 5783; 6284; 23175; 3764; 2213; 56994; 23604; 3687; 80005; 3001; 3003; 30009; 8809; 3821; 84525; 5551; 8807; 2833; 3458; 8810; 1231; 10219; 22936; 3587; 29957; 7133; 10875; 837; 834; 6280; 6279; 23191; 10602; 199; 2769; 23075; 23258; 953; 822; 3958; 6277; 6275; 6281; 3956; 302; 9516; 968; 23433;

© 2023 Fernandez-Martinez A et al. *JAMA Oncology.*

				941; 9173; 10995; 11110; 79026; 84564; 91663; 93992; 160851; 729230
**Tcells.CD8.Exhausted.vs.Naive.Metagene.1 Pauken Science.2016 PMID.27789795**	**T cells**	Signature	PMID.27789795	118788; 7227; 942; 2650; 941; 929; 952; 57823; 29992; 80219; 4067; 55300; 3552; 5272; 3606; 10410; 55281; 3601; 1390; 10950; 64135; 3627; 153020; 84419; 221079; 91543; 9246; 629; 3600; 8676; 51296; 3669; 6648; 840; 8743; 9636; 10581; 3587; 219972; 10561; 23429; 79931; 2820; 84930; 338339; 64108; 23424; 56829; 1326; 5359; 10260; 25932; 115361; 10018; 10135; 10538; 3428; 837; 3586; 597; 3559; 4938; 25801; 9034; 5610; 1536; 80231; 11274; 7128; 132864; 439996; 2643; 85463; 55013; 602; 10437; 7097; 3656; 3437; 54739; 4792; 8870; 1026; 4084; 3553; 6351; 3665; 23643; 26471; 1263; 6004; 3434; 6280; 2921; 3604; 9334; 29126; 64332; 127544; 64761; 1846; 5341; 64092; 196383; 2669; 5996; 6357; 4616; 5734; 1847; 8013; 65108; 7050; 4783; 22822; 79720; 467; 4929; 7832; 1843; 1958; 9592; 23764; 3164; 2355; 1051; 80824; 8376; 8374
**Tcells.CD8.Exhausted.vs.Naive.Metagene.3 Pauken Science.2016 PMID.27789795**	**T cells**	Signature	PMID.27789795	3001; 30009; 3897; 3458; 3683; 54843; 50515; 10219; 3672; 10663; 8809; 23191; 6284; 3903; 3092; 29887; 5795; 3915; 5551; 8411; 2207; 356; 4644; 158219; 54476; 56994; 960; 493869; 81615; 214; 23710; 2149; 2833; 64065; 9497; 8807; 22936; 1231; 6118; 93663; 3821; 195; 639; 53637; 22837; 79158; 29957; 307; 8836; 56925; 493; 80005; 3003; 23604; 27293; 54502; 3956; 5911; 1234; 411; 8993; 29851; 5243; 23589; 4542; 23208;

© 2023 Fernandez-Martinez A et al. *JAMA Oncology.*

				2591; 23175; 2745; 79156; 11034; 7133; 3684; 23692; 6279; 23433; 9516; 84868; 10549; 201633; 8320; 3800; 302; 8435; 2151; 10602; 8553; 8829; 3005; 333926; 5167; 7905; 57105; 11172; 834; 6310; 3764; 11082; 9749; 51201; 10930; 7498; 3595; 22856; 9839; 23075; 23258; 2213; 11163; 125488; 6095; 8530; 11332; 7703; 29969; 84525; 1154; 1493; 728392; 79180; 6352; 202; 5179; 116496; 6275; 140738; 197259; 1524; 3398; 11006; 3958; 22926; 6277; 55450; 824; 967; 51744; 11126; 10875; 4818; 199; 8819; 2012; 26031; 8810; 301; 5783; 10809; 953; 3687; 54206; 9173; 91663; 729230; 10995; 84564; 79026; 11110
**Tcells.CD8.Memory.vs.Naive.Metagene.1 Pauken Science.2016 PMID.27789795**	**T cells**	Signature	PMID.27789795	256586; 55450; 1154; 5269; 4953; 8784; 1493; 29969; 7133; 8810; 9446; 2012; 824; 56253; 140738; 837; 834; 5167; 115361; 8553; 1234; 10402; 3398; 79180; 8530; 9047; 11126; 8829; 8993; 84525; 8809; 356; 2833; 3458; 6352; 3001; 3003; 1231; 10219; 5243; 123; 6645; 836; 10549; 3587; 23258; 9094; 822; 6277; 6275; 6281; 3958; 3956; 302; 728392; 2745; 3099; 6302; 11332; 58485; 9055; 10875; 23643; 10437; 2207; 7305; 2123; 5359; 8411; 9516; 8935; 23433; 23191; 56925; 10995; 729230; 80792; 9173
**Tcells.CD8.Memory.vs.Naive.Metagene.2 Pauken Science.2016 PMID.27789795**	**T cells**	Signature	PMID.27789795	11238; 29851; 57105; 22856; 23092; 2769; 118788; 23075; 953; 8524; 23710; 27120; 94120; 6310; 493; 201633; 54836; 54843; 598; 26031; 245972; 411; 54476; 79158; 27293; 11082; 2081; 2591; 125488;

© 2023 Fernandez-Martinez A et al. *JAMA Oncology.*

				389072; 5576; 7703; 51201; 2151; 10663; 5795; 3903; 6118; 3897; 3672; 22837; 960; 5214; 4542; 6095; 195; 4644; 93663; 1509; 84650; 2213; 23692; 3687; 301; 23175; 10809; 23604; 3764; 3004; 80005; 11345; 3595; 30009; 4818; 2149; 5783; 3683; 5551; 56994; 8807; 3821; 9497; 57412; 493869; 5911; 1524; 9839; 53637; 154075; 2034; 79156; 197259; 54331; 158219; 33; 4635; 79634; 127124; 63951; 3006; 8819; 214; 50515; 116496; 307; 11163; 29887; 3005; 22926; 3915; 7905; 6284; 23589; 8836; 11034; 639; 81615; 202; 1891; 5613; 29957; 80150; 168537; 333926; 10930; 55794; 8347; 11172; 83988; 3820; 3684; 3459; 8905; 54502; 199; 644; 84868; 11110; 114242; 160851; 79026; 84564; 89875; 91663; 93992
**Tcells.CD8.Memory.vs.Naive.Metagene.3 Pauken Science.2016 PMID.27789795**	**T cells**	Signature	PMID.27789795	941; 23670; 3559; 3662; 6504; 10538; 64092; 112574; 81553; 3555; 22936; 7227; 952; 5606; 5272; 4860; 3627; 3600; 3665; 64135; 11274; 5341; 366; 153020; 5966; 11080; 23429; 3606; 10260; 8444; 221079; 9693; 80149; 56300; 8767; 1647; 10950; 29933; 1326; 255231; 3475; 54206; 597; 8870; 1847; 9034; 23135; 23764; 6351; 2643; 6648; 2180; 1844; 7124; 3553; 3552; 2921; 7128; 4792; 29126; 64332; 8740; 80231; 1992; 25932; 1051; 51296; 10602; 11213; 3303; 1536; 929; 6280; 6279; 59342; 2212; 3162; 29992; 84419; 11006; 942; 967; 4067; 6775; 57823; 132864; 196383; 1880; 25976; 57485; 79720; 7050; 30015; 4793; 5932; 3725;

© 2023 Fernandez-Martinez A et al. *JAMA Oncology.*

				6236; 10221; 6520; 2669; 5996; 1164; 2717; 9456; 255758; 4783; 388; 22822; 7832; 1843; 3726; 9592; 3164; 2355; 467; 80219; 2354; 1390; 4929; 5734; 8013; 84275; 10253; 6498; 10206; 9683; 80824; 7185; 3383; 330; 65108; 4616; 85463; 4794; 1026; 6675; 5292; 127544; 338339; 8878; 4780; 222255; 51278; 7850
**Serum.Response.Up TCGA Immunity.2018 PMID.29628290**	**T cells**	Signature	PMID.29 628290	84131; 27258; 55631; 9263; 6184; 3726; 79902; 2318; 3151; 10799; 29796; 7965; 1111; 51534; 11340; 79172; 56902; 6566; 79084; 51465; 51491; 4521; 10592; 292; 9818; 8847; 11333; 56267; 10063; 56647; 5340; 85397; 6631; 10733; 10204; 25804; 6611; 10969; 93081; 7416; 10213; 26292; 64750; 2791; 2152; 3575; 83990; 8399183990; 83991; 3181; 1633; 84266; 56263; 9086156263; 90861; 4478; 7168; 219844; 115106; 9688; 6635; 3655; 55839; 53838; 8836; 5214; 2193; 27102; 387103; 7277; 6434; 7372; 51154; 55270; 5237; 55276; 79007; 6628; 4331; 129401; 6921; 51668; 84798; 3399; 79711; 10056; 1981; 220134; 79157; 5329; 153562; 4172; 1719; 10193; 3398; 3015; 1017; 56926; 55055; 6993; 56942; 55972; 389; 22948; 9601; 6626; 10432; 9260; 26207; 7170; 23603; 10613; 5059; 106065059; 10606; 51002; 348235; 4603; 80851; 675; 56681; 51728; 28957; 6494728957; 64947; 57122; 7283; 5411; 83648; 5983; 4637; 4176; 55110; 23625; 10452; 55038; 4504; 4522; 5718; 4605; 51192; 56675; 7430;

© 2023 Fernandez-Martinez A et al. *JAMA Oncology.*

				29902; 2822; 6723; 5865; 57486; 4494; 51330; 7167; 3037; 79135; 150726; 51253; 79807; 6382; 84058; 51444; 378708; 64777; 2023; 1326; 222865; 9784; 11103; 6876; 5036; 8607; 6632; 4017; 5427; 22919; 92856; 2013; 5708; 4233; 7866; 3999; 848233999; 84823; 5352; 57552; 4830; 6800; 201254; 86; 10301; 6627; 10951; 55646; 9199; 5216; 55835; 23406; 57213; 10208; 6182; 9510; 10539; 55884; 51021; 6495951021; 64959; 64858; 8195; 84319; 80315; 200162; 359815; 6675; 27235; 11169; 131566; 23065; 51128; 5688; 5702; 10980; 1854; 10465; 26147; 7169; 28985; 1978; 10236
**Lymphocyte.Infiltration.Expression.Score TCGA Immunity.2018 PMID.29628290**	**T cells**	Signature	PMID.29628290	6352; 930; 951; 915; 916; 917; 919; 973; 974; 925; 926; 3502; 28219; 3512; 3537; 28840; 929; 3932; 4050; 931
**Tcells.Follicular.Helper TCGA Immunity.2018 PMID.29628290**	**T cells**	Signature	PMID.29628290	27087; 643; 753; 8851; 1114; 1117; 10563; 23462; 8362; 3382; 8645; 57535; 4094; 28986; 57496; 4646; 4647; 23178; 5133; 10585; 5783; 5816; 54436; 55423; 56301; 4086; 7972; 6489; 27347; 63892; 9760; 7253; 92595
**Tcells.Gamma.Delta TCGA Immunity.2018 PMID.29628290**	**T cells**	Signature	PMID.29628290	10485; 11126; 9638; 445347; 6964; 6983; 28531
**Immune.CD8.GZMK TCGA.BRCA.1198 JCI.2020 PMID.32573490**	**T cells**	Signature	PMID.32573490	729230; 497189; 1236; 2672; 387357; 84636; 387751; 10666; 959; 115352; 5133; 100188949; 120425; 9840; 56253; 8320; 283897; 5579; 22914; 256380; 29851; 201633; 29909; 151888; 128611; 925; 10663; 154075; 50852; 3003; 149628; 30009; 3001; 939; 4063; 6504; 4068; 114836; 917; 3702; 10225; 55423; 53347; 3932; 2833; 27240;

© 2023 Fernandez-Martinez A et al. *JAMA Oncology.*

				915; 914; 916; 919; 921; 3561; 962; 84174; 6352; 4818; 5551; 923; 79037; 3004; 5790; 8698; 7535; 9744; 1731; 374403; 64926; 147138; 11184; 11151; 9051; 27040; 3718; 9806; 11262; 8530; 1043; 924; 80342
**Immune.Cell.Content Verhaak NatCommun.2013 PMID.24113773**	**T cells**	Signature	PMID.24113773	10320; 9910; 3575; 55303; 3106; 3937; 4046; 2533; 5341; 3055; 3587; 10859; 3071; 3903; 4688; 1536; 5788; 7805; 963; 2124; 6503; 3689; 4542; 3059; 4332; 3561; 962; 313; 6352; 4050; 9535; 474344; 3003; 7940; 8477; 10288; 7456; 951; 51411; 2207; 7305; 10875; 2313; 3394; 55843; 10019; 7133; 1794; 914; 9459; 11151; 23643; 4069; 3683; 7128; 6039; 7040; 9051; 8530; 5996; 2268; 6402; 64780; 80342; 3676; 9935; 397; 3566; 399; 3113; 4818; 4689; 9404; 3702; 6404; 3115; 915; 11314; 3560; 113; 5734; 5552; 919; 1236; 4478; 241; 5732; 5880; 2634; 7409; 9976; 9934; 4792; 6280; 10437; 64747; 9770; 1200; 391; 50856; 3002; 79037; 6279; 834; 597; 3133; 3820; 10578; 5873; 8807; 8459; 2014; 51291; 3932; 9235; 5790; 3965; 26112; 25939; 6890; 2633; 1520; 2999; 101; 2745; 5551; 969; 3108; 972; 22914; 5791; 3122; 8875; 10312; 1475; 7535; 3134; 3135; 1043; 9936; 939
**TLS.Hallmark Cabrita Nature.2020 PMID.31942071**	**TLS**	Signature	PMID.31942071	6363; 6366; 10563; 1236; 643; 27074; 6402
**TLS.Known.Markers Cabrita Nature.2020 PMID.31942071**	**TLS**	Signature	PMID.31942071	1236; 10563; 6363; 7852; 6366; 27074; 942; 604; 6402
**TLS.12genes.Chemokine Zhu FrontImmunol.2017 PMID.28713385**	**TLS**	Signature	PMID.28713385	6348; 6347; 10563; 6366; 4283; 6355; 6363; 6352; 6373; 6351; 6362; 3627

© 2023 Fernandez-Martinez A et al. *JAMA Oncology.*

**TLS.High.In.No.Response Helmink Nature.2020 PMID.31942075**	TLS	Signature	PMID.31942075	51129; 7079; 80760; 4017; 5046; 5055; 374; 3451; 5212; 347; 8534; 133; 1917; 79884; 7056; 5522; 8862; 57722; 147495; 6098; 27295; 4099; 8515; 154664; 2260; 27237; 63982; 79054; 374946; 84929; 148281; 200162; 6620; 5009; 114788; 23743; 7087; 89886; 1770; 55511
**TLS.High.In.Response.MCP Helmink Nature.2020 PMID.31942075**	TLS	Signature	PMID.31942075	4283; 2633; 3001; 915; 3627; 8115; 973; 9235; 3620; 3512; 5450; 6890; 710; 51237; 3821; 923; 158471; 3659; 3458; 5920; 115361; 10396; 151888; 3824; 54900; 83416; 26279; 8368; 56145; 56146; 8395; 55118; 260436; 3738; 100423062; 91319; 389643
**TLS.9genes Cabrita Nature.2020 PMID.31942071**	TLS	Signature	PMID.31942071	5730; 912; 974; 1235; 27040; 1071; 83758; 9086; 8631
**TLS.Tumors.w.TLS.and.CD8.vs.CD8.alone Cabrita Nature.2020 PMID.31942071**	TLS	Signature	PMID.31942071	962; 4050; 1043; 915; 6402; 80342; 939; 51316; 9595; 973; 1236; 974; 4033; 952; 3957; 917; 919; 3512; 330; 51237; 6363; 8875; 6366; 730; 5790; 3112; 3394; 923; 27040; 912; 5730; 971; 54923; 1235; 7351; 54900; 64333; 374403; 81704; 11040; 9086; 54855; 1776; 5777; 5336; 8631; 55840; 114836; 160365; 202309; 115352; 83758; 1071; 79037

© 2023 Fernandez-Martinez A et al. *JAMA Oncology.*

eTable 2. Comparison of Baseline Characteristics of the Patients From the CALGB 40601 Event-Free Survival (EFS) and Landmark Subpopulations

Characteristic	CALGB40601 EFS cohort (N = 230)	CALGB40601 Landmark cohort (N = 227)	p-value
**Age (median, IQ range)**	49 (41, 56)	49 (41, 56)	NS
**Menopause status**			NS
Postmenopausal	89 (38.70%)	87 (38.33%)	
Premenopausal	141 (61.30%)	140 (61.67%)	
**HR status**			NS
HR-negative	93 (40.43%)	91 (40.09%)	
HR-positive	137 (59.57%)	136 (59.91%)	
**Clinical Stage**			NS
Stage I	0 (0%)	0 (0%)	
Stage II	157 (68.26%)	156 (68.72%)	
Stage III	73 (31.74%)	71 (31.28%)	
**Treatment**			NS
HL +/- ET	0 (0%)	0 (0%)	
TH	89 (38.70%)	87 (38.33%)	
THL	95 (41.30%)	95 (41.85%)	
TL	46 (20%)	45 (19.82%)	
**Intrinsic subtype**			NS
Basal-like	19 (8.26%)	19 (8.37%)	
HER2-Enriched	131 (56.96%)	129 (56.83%)	
Luminal A	26 (11.30%)	26 (11.45%)	
Luminal B	32 (13.91%)	31 (13.66%)	
Normal-like	22 (9.57%)	22 (9.69%)	

Statistical differences were assessed using a Wilcoxon rank sum test (for the age) and Pearson's Chi-squared test (for the rest of the variables).
EFS: event-free survival; IQ: interquartile range; HR: hormone receptor; H: trastuzumab; L: lapatinib; ET: endocrine therapy; T: weekly paclitaxel; NS: no significant.

© 2023 Fernandez-Martinez A et al. *JAMA Oncology.*

eTable 3. Correlation of Tumor-Infiltrating Lymphocytes (TILs) and Immune Gene Expression Signatures (iGES) in CALGB 40601 and PAMELA

CALGB 40601	Coefficient	p-value	Coefficient tertiles	p-value tertiles
Immune1 TCGA.BRCA.1198 JCI.2020 PMID.32573490	0.61	<0.001	0.53	<0.001
Stromal.Inflammation Heng JPathol.2017 PMID.27861902	0.6	<0.001	0.54	<0.001
TLS.High.In.Response.MCP Helmink Nature.2020 PMID.31942075	0.6	<0.001	0.53	<0.001
IFN.5.ImmLandscape Wolf PlosOne.2014 PMID.24516633	0.59	<0.001	0.55	<0.001
Tcells.CD4.Memory.Activated CIBERSORT NatMethods.2015 PMID.25822800	0.59	<0.001	0.57	<0.001
Plasma.cells ImSig.Nirmal CancerImmunolRes.2018 PMID.30266715	0.59	<0.001	0.51	<0.001
NK.CD56dim Bindea Immunity.2013 PMID.24138885	0.58	<0.001	0.52	<0.001
Immune.Active Hollern Cell.2019 PMID.31730857	0.57	<0.001	0.50	<0.001
CTLA4.Single.Gene Hollern Cell.2019 PMID.31730857	0.57	<0.001	0.51	<0.001
Tcells.Cluster Iglesia CCR.2014 PMID.24916698	0.56	<0.001	0.49	<0.001
Bcells.Plasma.cells.52genes Miller GenomeBiol.2013 PMID.23618380	0.56	<0.001	0.52	<0.001
IGG.Cluster Fan BMCMedGenomics.2011 PMID.21214954	0.56	<0.001	0.51	<0.001
PDCD1.Single.Gene Pare AnnOncol.2019 PMID.30165419	0.56	<0.001	0.50	<0.001
Macrophages.M1 CIBERSORT NatMethods.2015 PMID.25822800	0.55	<0.001	0.51	<0.001
Immune.CD8.GZMK TCGA.BRCA.1198 JCI.2020 PMID.32573490	0.55	<0.001	0.48	<0.001
CD8.Cluster Iglesia CCR.2014 PMID.24916698	0.55	<0.001	0.47	<0.001
Cytotoxic.Lymphocytes.MCP Petitprez Nature.2020 PMID.31942077	0.55	<0.001	0.51	<0.001
Cytotoxic.cells Bindea Immunity.2013 PMID.24138885	0.55	<0.001	0.49	<0.001
Tcells.Survival.2gene Petitprez Nature.2020 PMID.31942077	0.55	<0.001	0.49	<0.001
CTLA4.Pathway GSEA.BIOCARTA ProcNatlAcadSciUSA.2005 PMID.16199517	0.55	<0.001	0.49	<0.001
Immune.CTLA4.CXCL.FOXP3 TCGA.BRCA.1198 JCI.2020 PMID.32573490.	0.55	<0.001	0.52	<0.001
Immune.Suppression Kardos JCIInsight.2016 PMID.27699256	0.54	<0.001	0.52	<0.001
Tcells.CD8.Memory.vs.Naive.Metagene.3 Pauken Science.2016 PMID.27789795	0.54	<0.001	0.50	<0.001
Ig TCGA.BRCA.1198 Cell.2015 PMID.26451490	0.54	<0.001	0.49	<0.001

© 2023 Fernandez-Martinez A et al. *JAMA Oncology.*

Tcells.Gamma.Delta CIBERSORT NatMethods.2015 PMID.25822800	0.54	<0.001	0.45	<0.001
MERCK.Immune.Signature Cristescu Science.2018 PMID.30309915	0.54	<0.001	0.45	<0.001
Immune.87 Perez JCO.2015 PMID.2560586	0.54	<0.001	0.46	<0.001
Tcells.Th1.cells Bindea Immunity.2013 PMID.24138885	0.54	<0.001	0.53	<0.001
CD274.Single.Gene Hollern Cell.2019 PMID.31730857	0.54	<0.001	0.51	<0.001
Bcells.Immature Charoentong CellRep.2017 PMID.28052254	0.54	<0.001	0.48	<0.001
Tcells.CD8 CIBERSORT NatMethods.2015 PMID.25822800	0.53	<0.001	0.47	<0.001
NK.Resting CIBERSORT NatMethods.2015 PMID.25822800	0.53	<0.001	0.45	<0.001
Immune.cell.Cluster.PerouLab Fan.GSEA.GP2 BMCMedGenomics.2011 PMID.21214954	0.53	<0.001	0.51	<0.001
Tcells.MCP Petitprez Nature.2020 PMID.31942077	0.53	<0.001	0.48	<0.001
Immune.Hot.vs.Cold.CD8 Cabrita Nature.2020 PMID.31942071	0.53	<0.001	0.48	<0.001
Immune.CD4.CD53.CD84.BTK TCGA.BRCA.1198 JCI.2020 PMID.32573490	0.53	<0.001	0.48	<0.001
MHC.II Rody BreastCancerResearch.2008 PMID.19272155	0.53	<0.001	0.47	<0.001
Tcells.Resident.Memory.Single.cell Savas NatMed.2018 PMID.29942092	0.52	<0.001	0.46	<0.001
Tcells.Follicular.Helper CIBERSORT NatMethods.2015 PMID.25822800	0.52	<0.001	0.45	<0.001
Tcells.CD4.Memory.Resting CIBERSORT NatMethods.2015 PMID.25822800	0.52	<0.001	0.42	<0.001
Tcells Bindea Immunity.2013 PMID.24138885	0.52	<0.001	0.42	<0.001
Tcells.Th1.cells Charoentong CellRep.2017 PMID.28052254	0.52	<0.001	0.46	<0.001
Cytolytic.Activity Rooney Cell.2015 PMID.25594174	0.51	<0.001	0.45	<0.001
NK ImSig.Nirmal CancerImmunolRes.2018 PMID.30266715	0.51	<0.001	0.48	<0.001
MCD3.CD8 Fan BMCMedGenomics.2011 PMID.21214954	0.51	<0.001	0.43	<0.001
NK.Activated CIBERSORT NatMethods.2015 PMID.25822800	0.51	<0.001	0.46	<0.001
Dendritic.cells.Activated Bindea Immunity.2013 PMID.24138885	0.51	<0.001	0.51	<0.001
Macrophages.Th1.Cluster Iglesia CCR.2014 PMID.24916698	0.51	<0.001	0.48	<0.001
Tcells.CD8.Activated Charoentong CellRep.2017 PMID.28052254	0.51	<0.001	0.45	<0.001
STAT1 Rody BreastCancerResearch.2008 PMID.19272155	0.5	<0.001	0.48	<0.001
Tcells.Regulatory.Tregs CIBERSORT NatMethods.2015 PMID.25822800	0.5	<0.001	0.41	<0.001

© 2023 Fernandez-Martinez A et al. *JAMA Oncology.*

TLS.Tumors.w.TLS.and.CD8.vs.CD8.alone Cabrita Nature.2020 PMID.31942071	0.5	<0.001	0.44	<0.001
Plasma.cells CIBERSORT NatMethods.2015 PMID.25822800	0.5	<0.001	0.45	<0.001
Bcells.IL10.Plus Lin JImmunol.2014 PMID.25080484	0.5	<0.001	0.48	<0.001
Macrophages.Monocytes.CSF1.Response Beck CCR.2009 PMID.19188147	0.5	<0.001	0.49	<0.001
CSF1.Response TCGA Immunity.2018 PMID.29628290	0.5	<0.001	0.49	<0.001
MDSC Charoentong CellRep.2017 PMID.28052254	0.5	<0.001	0.50	<0.001
Tcells.CD8.Exhausted.at.day.8.post.Imm.vs. Naive.Metagene.1 Pauken Science.2016 PMID.27789795	0.5	<0.001	0.47	<0.001
Bcells.Cluster Iglesia CCR.2014 PMID.24916698	0.5	<0.001	0.48	<0.001
Tcells.CD8.Memory.vs.Naive.1 Pauken Science.2016 PMID.27789795	0.5	<0.001	0.45	<0.001
Tcells.CD8.Memory.vs.Naive.Metagene.1 Pauken Science.2016 PMID.27789795	0.5	<0.001	0.45	<0.001
Tcells.NK.51genes Miller GenomeBiol.2013 PMID.23618380	0.49	<0.001	0.42	<0.001
Immune.Cell.Content Verhaak NatCommun.2013 PMID.24113773	0.49	<0.001	0.46	<0.001
Tcells ImSig.Nirmal CancerImmunolRes.2018 PMID.30266715	0.49	<0.001	0.42	<0.001
LCK Rody BreastCancerResearch.2008 PMID.19272155	0.49	<0.001	0.46	<0.001
Tcells.Bcells.Lymphocyte.Infiltration Calabro BreastCancerResTreat.2009 PMID.18592372	0.49	<0.001	0.39	<0.001
Lymphocyte.Infiltration.Expression.Score TCGA Immunity.2018 PMID.29628290	0.49	<0.001	0.39	<0.001
Tcells.CD8.Exhausted.vs.Naive.Metagene.1 Pauken Science.2016 PMID.27789795	0.49	<0.001	0.46	<0.001
Tcells.CD4.Activated Charoentong CellRep.2017 PMID.28052254	0.49	<0.001	0.48	<0.001
Tcells.CD8.Exhausted.vs.Naive.Metagene.3 Pauken Science.2016 PMID.27789795	0.49	<0.001	0.43	<0.001
IgG Rody BreastCancerResearch.2008 PMID.19272155	0.48	<0.001	0.44	<0.001
TLS.12genes.Chemokine Zhu FrontImmunol.2017 PMID.28713385	0.48	<0.001	0.45	<0.001
Tcells.Bcell.KEGG.hematopoietic.cell.lineage GSEA.GP2 ProcNatlAcadSciUSA.2005 PMID.16199517	0.48	<0.001	0.42	<0.001
Tcells.NK.Metagene Miller GenomeBiol.2013 PMID.23618380	0.48	<0.001	0.44	<0.001
Monocytes.Dendritic.25genes Miller GenomeBiol.2013 PMID.23618380	0.48	<0.001	0.46	<0.001
Bcells ImSig.Nirmal CancerImmunolRes.2018 PMID.30266715	0.48	<0.001	0.43	<0.001
Tcells.CD4.Naive CIBERSORT NatMethods.2015 PMID.25822800	0.47	<0.001	0.41	<0.001

© 2023 Fernandez-Martinez A et al. *JAMA Oncology.*

Bcells.Plasma.cells.Metagene Miller GenomeBiol.2013 PMID.23618380	0.47	<0.001	0.43	<0.001
Tcells.CD8.MCP Petitprez Nature.2020 PMID.31942077	0.46	<0.001	0.35	<0.001
Bcells.Plasmablast Dybaer JCO.2015 PMID.25800755	0.46	<0.001	0.39	<0.001
Dendritic.cells.Activated CIBERSORT NatMethods.2015 PMID.25822800	0.46	<0.001	0.44	<0.001
Bcells.Memory CIBERSORT NatMethods.2015 PMID.25822800	0.46	<0.001	0.41	<0.001
Tcells.CD8.Memory.vs.Naive.Metagene.2 Pauken Science.2016 PMID.27789795	0.46	<0.001	0.41	<0.001
MHC.11genes Forero CancerImmunolRes.2016 PMID.26980599	0.46	<0.001	0.42	<0.001
Immune.HLA.D TCGA.BRCA.1198 JCI.2020 PMID.32573490	0.46	<0.001	0.38	<0.001
Tcells.CD8.Effector.Memory Charoentong CellRep.2017 PMID.28052254	0.45	<0.001	0.40	<0.001
Immune.CD19 TCGA.BRCA.1198 JCI.2020 PMID.32573490	0.45	<0.001	0.38	<0.001
Tcells.Regulatory Charoentong CellRep.2017 PMID.28052254	0.45	<0.001	0.43	<0.001
Tcells.Activation Petitprez Nature.2020 PMID.31942077	0.45	<0.001	0.42	<0.001
Dendritic.cells.Activated Charoentong CellRep.2017 PMID.28052254	0.45	<0.001	0.44	<0.001
Bcells Bindea Immunity.2013 PMID.24138885	0.44	<0.001	0.37	<0.001
Bcells.Naive CIBERSORT NatMethods.2015 PMID.25822800	0.44	<0.001	0.39	<0.001
PD1.Signaling.Reactome GSEA ProcNatlAcadSciUSA.2005 PMID.16199517	0.44	<0.001	0.41	<0.001
Bcells.ImmuneProfiles.Mouse.Human Shay PNAS.2013 PMID.23382184	0.43	<0.001	0.34	<0.001
Immune.HLA.A.F TCGA.BRCA.1198 JCI.2020 PMID.32573490	0.43	<0.001	0.41	<0.001
Bcells.Activated Charoentong CellRep.2017 PMID.28052254	0.43	<0.001	0.36	<0.001
Macrophages ImSig.Nirmal CancerImmunolRes.2018 PMID.30266715	0.43	<0.001	0.46	<0.001
Monocytes.Dendritic.cell.Metagene Miller GenomeBiol.2013 PMID.23618380	0.43	<0.001	0.34	<0.001
MHC.I Rody BreastCancerResearch.2008 PMID.19272155	0.42	<0.001	0.35	<0.001
Neutrophils.Activated.Lung Janiszewska NatCellBiol.2019 PMID.31263265	0.42	<0.001	0.36	<0.001
MHC.I.CoreGenes Lauss NatCommun.2017 PMID29170503	0.41	<0.001	0.38	<0.001
Bcells Garber CellMolGastroenterolHepatol.2017 PMID.28508029	0.41	<0.001	0.38	<0.001
NK.MCP Helmink Nature.2020 PMID.31942077	0.41	<0.001	0.29	<0.001
Monocytes CIBERSORT NatMethods.2015 PMID.25822800	0.41	<0.001	0.45	<0.001

© 2023 Fernandez-Martinez A et al. *JAMA Oncology.*

HCK Rody BreastCancerResearch.2008 PMID.19272155	0.41	<0.001	0.43	<0.001
TLS.Known.Markers Cabrita Nature.2020 PMID.31942071	0.4	<0.001	0.36	<0.001
Monocytes ImSig.Nirmal CancerImmunolRes.2018 PMID.30266715	0.4	<0.001	0.35	<0.001
MDSC.Neutrophil Youn LeukocBiol.2012 PMID.21954284	0.39	<0.001	0.36	<0.001
Macrophages.M0 CIBERSORT NatMethods.2015 PMID.25822800	0.39	<0.001	0.44	<0.001
TLS.Hallmark Cabrita Nature.2020 PMID.31942071	0.37	<0.001	0.30	<0.001
Tcells.ImmuneProfiles.Mouse.Human Shay PNAS.2013 PMID.23382184	0.37	<0.001	0.34	<0.001
Tcells.Central.Memory Bindea Immunity.2013 PMID.24138885	0.37	<0.001	0.26	<0.001
Neutrophils CIBERSORT NatMethods.2015 PMID.25822800	0.37	<0.001	0.36	<0.001
Bcells.Tcells.Cooperation Hollern Cell.2019 PMID.31730857	0.35	<0.001	0.28	<0.001
Immune.GIMAP.IL16 TCGA.BRCA.1198 JCI.2020 PMID.32573490	0.35	<0.001	0.32	<0.001
Tcells.CD8.Exhausted.Anti.PDL1.vs.Control. Metagene.1 Pauken Science.2016 PMID.27789795	0.35	<0.001	0.39	<0.001
IFNg.Module11 Gatza ProcNatlAcadSciUSA.2010 PMID.20335537	0.35	<0.001	0.36	<0.001
IFN.Cluster.GSEA.GP11 Fan BMCMedGenomics.2011 PMID.21214954	0.35	<0.001	0.37	<0.001
Bcells.IL10.Minus Lin JImmunol.2014 PMID.25080484	0.33	<0.001	0.27	<0.001
Monocytes Charoentong CellRep.2017 PMID.28052254	0.33	<0.001	0.27	<0.001
Dendritic.cells.Resting CIBERSORT NatMethods.2015 PMID.25822800	0.33	<0.001	0.32	<0.001
Eosinophils CIBERSORT NatMethods.2015 PMID.25822800	0.33	<0.001	0.31	<0.001
TLS.9genes Cabrita Nature.2020 PMID.31942071	0.32	<0.001	0.24	<0.001
Bcells.Extended Garber CellMolGastroenterolHepatol.2017 PMID.28508029	0.31	<0.001	0.29	<0.001
Macrophages Bindea Immunity.2013 PMID.24138885	0.31	<0.001	0.31	<0.001
Tcells.Gamma.Delta Bindea Immunity.2013 PMID.24138885	0.31	<0.001	0.26	<0.001
Tcells.Gamma.Delta TCGA Immunity.2018 PMID.29628290	0.31	<0.001	0.26	<0.001
Tcells.CD8.Exhausted.vs.AntiPDL1.2 Pauken Science.2016 PMID.27789795	0.31	<0.001	0.28	<0.001
Tcells.Follicular.Helper Bindea Immunity.2013 PMID.24138885	0.3	<0.001	0.26	<0.001
Tcells.Follicular.Helper TCGA Immunity.2018 PMID.29628290	0.3	<0.001	0.26	<0.001

© 2023 Fernandez-Martinez A et al. *JAMA Oncology.*

Macrophages.M2 CIBERSORT NatMethods.2015 PMID.25822800	0.3	<0.001	0.27	<0.001
Bcells.Centrocyte Dybaer JCO.2015 PMID.25800755	0.28	<0.001	0.25	<0.001
MDSC.Tumor.Macrophages Schlecker JImmunol.2012 PMID.23152559	0.28	<0.001	0.30	<0.001
Bcells.Memory Dybaer JCO.2015 PMID.25800755	0.28	<0.001	0.27	<0.001
IFNa.Module10 Gatza ProcNatlAcadSciUSA.2010 PMID.20335537	0.28	<0.001	0.33	<0.001
Granulocytes.ImmuneProfiles.Mouse.Human Shay PNAS.2013 PMID.23382184	0.28	<0.001	0.28	<0.001
Dendritic.cells Bindea Immunity.2013 PMID.24138885	0.26	<0.001	0.26	<0.001
IFN.Pathway ImSig.Nirmal CancerImmunolRes.2018 PMID.30266715	0.26	<0.001	0.29	<0.001
Tcells.CD8.Exhausted.vs.Naive.2 Pauken Science.2016 PMID.27789795	0.25	0.001	0.23	0.001
Influenza.11genes Khatri Immunity.2015 PMID.26682989	0.25	<0.001	0.26	<0.001
Immune.14 Perez JCO.2015 PMID.2560586	0.24	<0.001	0.23	0.001
Macrophages.M2 Ghassabeh Blood.2006 PMID.16556895	0.23	0.001	0.22	0.001
CD68.Cluster Iglesia CCR.2014 PMID.24916698	0.23	0.001	0.21	0.002
Proliferation.Pathway ImSig.Nirmal CancerImmunolRes.2018 PMID.30266715	0.23	0.003	0.22	0.001
Tcells.CD8.Effector.vs.Naive.2 Pauken Science.2016 PMID.27789795	0.23	0.003	0.22	0.001
Tcells.CD4.Effector.Memory Charoentong CellRep.2017 PMID.28052254	0.23	<0.001	0.25	<0.001
Mast.cells.Resting CIBERSORT NatMethods.2015 PMID.25822800	0.23	<0.001	0.18	0.01
Mast.cell Charoentong CellRep.2017 PMID.28052254	0.22	0.001	0.23	0.001
Tcells.Regulatory.cell.2gene Petitprez Nature.2020 PMID.31942077	0.22	0.001	0.17	0.01
Neutrophils ImSig.Nirmal CancerImmunolRes.2018 PMID.30266715	0.22	<0.001	0.27	<0.001
Denditic.cells.ImmuneProfiles.Mouse.Human Shay PNAS.2013 PMID.23382184	0.21	0.003	0.24	<0.001
Bcells.Centroblast Dybaer JCO.2015 PMID.25800755	0.21	0.004	0.16	0.02
Tcells.Follicular.Helper Charoentong CellRep.2017 PMID.28052254	0.2	0.001	0.21	0.002
MDSC.Tumor Schlecker JImmunol.2012 PMID.23152559	0.2	0.001	0.22	0.001
Serum.Response.Up TCGA Immunity.2018 PMID.29628290	0.2	0.001	0.12	0.07
Neutrophils Bindea Immunity.2013 PMID.24138885	0.2	0.013	0.23	0.001
Tcells.Th2 Charoentong CellRep.2017 PMID.28052254	0.18	<0.001	0.17	0.01

© 2023 Fernandez-Martinez A et al. *JAMA Oncology.*

Bcells.Naive Dybaer JCO.2015 PMID.25800755	0.17	0.002	0.14	0.04
NK.ImmuneProfiles.Mouse.Human Shay PNAS.2013 PMID.23382184	0.17	0.002	0.17	0.01
Wound.Healing Chang PlosBiol.2004 PMID.14737219	0.17	0.003	0.15	0.03
MHC.24genes Forero CancerImmunolRes.2016 PMID.26980599	0.17	0.008	0.15	0.03
Mast.cells.Activated CIBERSORT NatMethods.2015 PMID.25822800	0.17	0.009	0.13	0.07
NK Charoentong CellRep.2017 PMID.28052254	0.17	0.01	0.17	0.01
NK.Tcell Charoentong CellRep.2017 PMID.28052254	0.17	0.01	0.22	0.001
IFN.Cluster Fan BMCMedGenomics.2011 PMID.21214954	0.17	0.02	0.20	0.003
Immune.IFN TCGA.BRCA.1198 JCI.2020 PMID.32573490	0.17	0.03	0.20	0.003
Tcells.CD8 Bindea Immunity.2013 PMID.24138885	0.17	0.03	0.05	0.47
IFN.3.ImmLandscape Wolf PlosOne.2014 PMID.24516633	0.17	0.04	0.20	0.002
IFN.Score.module3 TCGA Immunity.2018 PMID.29628290	0.17	0.04	0.20	0.002
Eosinophils Charoentong CellRep.2017 PMID.28052254	0.15	0.03	0.17	0.014
Tcells.Thelper Bindea Immunity.2013 PMID.24138885	0.14	0.012	0.07	0.29
Tcells.Th17.cells Charoentong CellRep.2017 PMID.28052254	0.11	0.03	0.08	0.22
Dendritic.cells.Immature. Charoentong CellRep.2017 PMID.28052254	0.1	0.04	0.14	0.05
Mast.cells Bindea Immunity.2013 PMID.24138885	-0.14	0.04	-0.10	0.14
Eosinophils Bindea Immunity.2013 PMID.24138885	-0.16	0.03	-0.07	0.34
Immunosuppression Petitprez Nature.2020 PMID.31942077	-0.17	0.01	-0.11	0.10
Tcells.Th17.cells Bindea Immunity.2013 PMID.24138885	-0.18	0.04	-0.16	0.02
TGFB.score TCGA Immunity.2018 PMID.29628290	-0.26	<0.001	-0.19	0.006
TLS.High.In.No.Response Helmink Nature.2020 PMID.31942075	-0.29	<0.001	-0.24	<0.001
**PAMELA**	**Coefficient**	**p-value**		
IFN.5.ImmLandscape Wolf PlosOne.2014 PMID.24516633	0.71	<0.001	0.64	<0.001
Stromal.Inflammation Heng JPathol.2017 PMID.27861902	0.7	<0.001	0.63	<0.001
TLS.High.In.Response.MCP Helmink Nature.2020 PMID.31942075	0.65	<0.001	0.58	<0.001
Tcells.Bcell.Cluster Fan BMCMedGenomics.2011 PMID.21214954	0.68	<0.001	0.62	<0.001

© 2023 Fernandez-Martinez A et al. *JAMA Oncology.*

Tcells.CD4.Memory.Activated CIBERSORT NatMethods.2015 PMID.25822800	0.69	<0.001	0.62	<0.001
Immune.Active Hollern Cell.2019 PMID.31730857	0.67	<0.001	0.62	<0.001
Tcells.Cluster Iglesia CCR.2014 PMID.24916698	0.66	<0.001	0.64	<0.001
Tcells.CD8 CIBERSORT NatMethods.2015 PMID.25822800	0.65	<0.001	0.64	<0.001
CD8.Cluster Iglesia CCR.2014 PMID.24916698	0.65	<0.001	0.62	<0.001
Cytotoxic.cells Bindea Immunity.2013 PMID.24138885	0.66	<0.001	0.62	<0.001
Immune.CD8.GZMK TCGA.BRCA.1198 JCI.2020 PMID.32573490	0.65	<0.001	0.62	<0.001
Macrophages.M1 CIBERSORT NatMethods.2015 PMID.25822800	0.68	<0.001	0.62	<0.001
Tcells ImSig.Nirmal CancerImmunolRes.2018 PMID.30266715	0.63	<0.001	0.63	<0.001
NK.Activated CIBERSORT NatMethods.2015 PMID.25822800	0.63	<0.001	0.59	<0.001
Tcells.Resident.Memory.Single.cell Savas NatMed.2018 PMID.29942092	0.64	<0.001	0.60	<0.001
Immune.Hot.vs.Cold.CD8 Cabrita Nature.2020 PMID.31942071	0.66	<0.001	0.64	<0.001
Tcells.Follicular.Helper CIBERSORT NatMethods.2015 PMID.25822800	0.65	<0.001	0.61	<0.001
Immune.87 Perez JCO.2015 PMID.2560586	0.65	<0.001	0.62	<0.001
Immune.Cell.Content Verhaak NatCommun.2013 PMID.24113773	0.64	<0.001	0.63	<0.001
Tcells.Gamma.Delta CIBERSORT NatMethods.2015 PMID.25822800	0.61	<0.001	0.54	<0.001
LCK Rody BreastCancerResearch.2008 PMID.19272155	0.64	<0.001	0.59	<0.001
Tcells.CD4.Memory.Resting CIBERSORT NatMethods.2015 PMID.25822800	0.63	<0.001	0.63	<0.001
MCD3.CD8 Fan BMCMedGenomics.2011 PMID.21214954	0.62	<0.001	0.54	<0.001
Tcells.MCP Petitprez Nature.2020 PMID.31942077	0.66	<0.001	0.57	<0.001
Tcells.CD8.Activated Charoentong CellRep.2017 PMID.28052254	0.65	<0.001	0.57	<0.001
TLS.Tumors.w.TLS.and.CD8.vs.CD8.alone Cabrita Nature.2020 PMID.31942071	0.62	<0.001	0.59	<0.001
Tcells Bindea Immunity.2013 PMID.24138885	0.64	<0.001	0.62	<0.001
Tcells.NK.51genes Miller GenomeBiol.2013 PMID.23618380	0.63	<0.001	0.59	<0.001
MERCK.Immune.Signature Cristescu Science.2018 PMID.30309915	0.68	<0.001	0.68	<0.001
NK.Resting CIBERSORT NatMethods.2015 PMID.25822800	0.62	<0.001	0.55	<0.001
Tcells.Bcells.Lymphocyte.Infiltration Calabro BreastCancerResTreat.2009 PMID.18592372	0.63	<0.001	0.57	<0.001

© 2023 Fernandez-Martinez A et al. *JAMA Oncology.*

Lymphocyte.Infiltration.Expression.Score TCGA Immunity.2018 PMID.29628290	0.63	<0.001	0.57	<0.001
Tcells.NK.Metagene Miller GenomeBiol.2013 PMID.23618380	0.61	<0.001	0.56	<0.001
Immune.CTLA4.CXCL.FOXP3 TCGA.BRCA.1198 JCI.2020 PMID.32573490.	0.71	<0.001	0.67	<0.001
NK.CD56dim Bindea Immunity.2013 PMID.24138885	0.64	<0.001	0.61	<0.001
CTLA4.Pathway GSEA.BIOCARTA ProcNatlAcadSciUSA.2005 PMID.16199517	0.65	<0.001	0.61	<0.001
Dendritic.cells.Activated Bindea Immunity.2013 PMID.24138885	0.65	<0.001	0.65	<0.001
Immune.CD4.CD53.CD84.BTK TCGA.BRCA.1198 JCI.2020 PMID.32573490	0.62	<0.001	0.62	<0.001
Cytotoxic.Lymphocytes.MCP Petitprez Nature.2020 PMID.31942077	0.6	<0.001	0.57	<0.001
TLS.12genes.Chemokine Zhu FrontImmunol.2017 PMID.28713385	0.66	<0.001	0.64	<0.001
Tcells.Regulatory.Tregs CIBERSORT NatMethods.2015 PMID.25822800	0.61	<0.001	0.57	<0.001
IGG.Cluster Fan BMCMedGenomics.2011 PMID.21214954	0.62	<0.001	0.52	<0.001
Macrophages.Th1.Cluster Iglesia CCR.2014 PMID.24916698	0.63	<0.001	0.62	<0.001
Tcells.CD4.Naive CIBERSORT NatMethods.2015 PMID.25822800	0.59	<0.001	0.59	<0.001
Immune.Suppression Kardos JCIInsight.2016 PMID.27699256	0.67	<0.001	0.64	<0.001
Immune1 TCGA.BRCA.1198 JCI.2020 PMID.32573490	0.61	<0.001	0.54	<0.001
Tcells.CD4.Activated Charoentong CellRep.2017 PMID.28052254	0.64	<0.001	0.60	<0.001
Macrophages.Monocytes.CSF1.Response Beck CCR.2009 PMID.19188147	0.62	<0.001	0.60	<0.001
CSF1.Response TCGA Immunity.2018 PMID.29628290	0.62	<0.001	0.60	<0.001
Tcells.Bcell.KEGG.hematopoietic.cell.lineage GSEA.GP2 ProcNatlAcadSciUSA.2005 PMID.16199517	0.62	<0.001	0.59	<0.001
STAT1 Rody BreastCancerResearch.2008 PMID.19272155	0.68	<0.001	0.66	<0.001
CD274.Single.Gene Hollern Cell.2019 PMID.31730857	0.65	<0.001	0.64	<0.001
Bcells.IL10.Plus Lin JImmunol.2014 PMID.25080484	0.58	<0.001	0.51	<0.001
Tcells.CD8.Effector.Memory Charoentong CellRep.2017 PMID.28052254	0.59	<0.001	0.58	<0.001
MHC.II Rody BreastCancerResearch.2008 PMID.19272155	0.65	<0.001	0.63	<0.001
Tcells.CD8.Exhausted.at.day.8.post.Imm.vs. Naive.Metagene.1 Pauken Science.2016 PMID.27789795	0.62	<0.001	0.56	<0.001
Dendritic.cells.Activated CIBERSORT NatMethods.2015 PMID.25822800	0.64	<0.001	0.61	<0.001

© 2023 Fernandez-Martinez A et al. *JAMA Oncology.*

Cytolytic.Activity Rooney Cell.2015 PMID.25594174	0.61	<0.001	0.59	<0.001
Tcells.Th1.cells Bindea Immunity.2013 PMID.24138885	0.57	<0.001	0.54	<0.001
Tcells.CD8.Memory.vs.Naive.1 Pauken Science.2016 PMID.27789795	0.64	<0.001	0.60	<0.001
Tcells.CD8.Memory.vs.Naive.Metagene.1 Pauken Science.2016 PMID.27789795	0.64	<0.001	0.60	<0.001
Bcells.Immature Charoentong CellRep.2017 PMID.28052254	0.57	<0.001	0.56	<0.001
Immune.GIMAP.IL16 TCGA.BRCA.1198 JCI.2020 PMID.32573490	0.51	<0.001	0.51	<0.001
TLS.Known.Markers Cabrita Nature.2020 PMID.31942071	0.58	<0.001	0.53	<0.001
Immune.HLA.A.F TCGA.BRCA.1198 JCI.2020 PMID.32573490	0.62	<0.001	0.60	<0.001
NK ImSig.Nirmal CancerImmunolRes.2018 PMID.30266715	0.55	<0.001	0.52	<0.001
Tcells.CD8.Exhausted.vs.Naive.Metagene.3 Pauken Science.2016 PMID.27789795	0.61	<0.001	0.58	<0.001
Bcells.Memory CIBERSORT NatMethods.2015 PMID.25822800	0.57	<0.001	0.47	<0.001
Tcells.CD8.Memory.vs.Naive.Metagene.3 Pauken Science.2016 PMID.27789795	0.61	<0.001	0.55	<0.001
Tcells.CD8.MCP Petitprez Nature.2020 PMID.31942077	0.65	<0.001	0.66	<0.001
Plasma.cells CIBERSORT NatMethods.2015 PMID.25822800	0.55	<0.001	0.47	<0.001
Monocytes.Dendritic.25genes Miller GenomeBiol.2013 PMID.23618380	0.6	<0.001	0.59	<0.001
Bcells ImSig.Nirmal CancerImmunolRes.2018 PMID.30266715	0.54	<0.001	0.47	<0.001
TLS.Hallmark Cabrita Nature.2020 PMID.31942071	0.54	<0.001	0.48	<0.001
MDSC Charoentong CellRep.2017 PMID.28052254	0.57	<0.001	0.50	<0.001
HCK Rody BreastCancerResearch.2008 PMID.19272155	0.61	<0.001	0.56	<0.001
Bcells.Naive CIBERSORT NatMethods.2015 PMID.25822800	0.53	<0.001	0.44	<0.001
Macrophages.M0 CIBERSORT NatMethods.2015 PMID.25822800	0.62	<0.001	0.60	<0.001
CTLA4.Single.Gene Hollern Cell.2019 PMID.31730857	0.66	<0.001	0.63	<0.001
Macrophages ImSig.Nirmal CancerImmunolRes.2018 PMID.30266715	0.63	<0.001	0.63	<0.001
Bcells.Plasma.cells.52genes Miller GenomeBiol.2013 PMID.23618380	0.57	<0.001	0.49	<0.001
Tcells.Th1.cells Charoentong CellRep.2017 PMID.28052254	0.57	<0.001	0.55	<0.001
Bcells.Cluster Iglesia CCR.2014 PMID.24916698	0.53	<0.001	0.44	<0.001
Tcells.Regulatory Charoentong CellRep.2017 PMID.28052254	0.55	<0.001	0.50	<0.001

© 2023 Fernandez-Martinez A et al. *JAMA Oncology.*

Ig TCGA.BRCA.1198 Cell.2015 PMID.26451490	0.55	<0.001	0.49	<0.001
Monocytes ImSig.Nirmal CancerImmunolRes.2018 PMID.30266715	0.6	<0.001	0.57	<0.001
Tcells.CD8.Exhausted.vs.Naive.Metagene.1 Pauken Science.2016 PMID.27789795	0.56	<0.001	0.53	<0.001
Bcells.Tcells.Cooperation Hollern Cell.2019 PMID.31730857	0.5	<0.001	0.40	<0.001
Bcells.Activated Charoentong CellRep.2017 PMID.28052254	0.52	<0.001	0.41	<0.001
Bcells Bindea Immunity.2013 PMID.24138885	0.5	<0.001	0.42	<0.001
Macrophages.M2 CIBERSORT NatMethods.2015 PMID.25822800	0.54	<0.001	0.61	<0.001
Tcells.CD8.Memory.vs.Naive.Metagene.2 Pauken Science.2016 PMID.27789795	0.54	<0.001	0.56	<0.001
MHC.I Rody BreastCancerResearch.2008 PMID.19272155	0.56	<0.001	0.55	<0.001
Immune.HLA.D TCGA.BRCA.1198 JCI.2020 PMID.32573490	0.55	<0.001	0.50	<0.001
Dendritic.cells.Activated Charoentong CellRep.2017 PMID.28052254	0.54	<0.001	0.52	<0.001
PD1.Signaling.Reactome GSEA ProcNatlAcadSciUSA.2005 PMID.16199517	0.5	<0.001	0.50	<0.001
Dendritic.cells.Resting CIBERSORT NatMethods.2015 PMID.25822800	0.55	<0.001	0.55	<0.001
TLS.9genes Cabrita Nature.2020 PMID.31942071	0.45	<0.001	0.42	<0.001
MHC.I.CoreGenes Lauss NatCommun.2017 PMID29170503	0.57	<0.001	0.59	<0.001
Tcells.ImmuneProfiles.Mouse.Human Shay PNAS.2013 PMID.23382184	0.39	<0.001	0.43	<0.001
Bcells.Plasma.cells.Metagene Miller GenomeBiol.2013 PMID.23618380	0.52	<0.001	0.44	<0.001
Immune.CD19 TCGA.BRCA.1198 JCI.2020 PMID.32573490	0.5	<0.001	0.43	<0.001
Bcells Garber CellMolGastroenterolHepatol.2017 PMID.28508029	0.51	<0.001	0.45	<0.001
MDSC.Neutrophil Youn LeukocBiol.2012 PMID.21954284	0.55	<0.001	0.54	<0.001
MHC.11genes Forero CancerImmunolRes.2016 PMID.26980599	0.47	<0.001	0.39	<0.001
Monocytes CIBERSORT NatMethods.2015 PMID.25822800	0.55	<0.001	0.51	<0.001
Bcells.Plasmablast Dybaer JCO.2015 PMID.25800755	0.47	<0.001	0.40	<0.001
Tcells.CD8.Exhausted.Anti.PDL1.vs.Control. Metagene.1 Pauken Science.2016 PMID.27789795	0.52	<0.001	0.53	<0.001
IFN.Cluster.GSEA.GP11 Fan BMCMedGenomics.2011 PMID.21214954	0.53	<0.001	0.48	<0.001
Plasma.cells ImSig.Nirmal CancerImmunolRes.2018 PMID.30266715	0.52	<0.001	0.45	<0.001

© 2023 Fernandez-Martinez A et al. *JAMA Oncology.*

IFNg.Module11 Gatza ProcNatlAcadSciUSA.2010 PMID.20335537	0.51	<0.001	0.49	<0.001
Monocytes.Dendritic.cell.Metagene Miller GenomeBiol.2013 PMID.23618380	0.53	<0.001	0.59	<0.001
Tcells.Activation Petitprez Nature.2020 PMID.31942077	0.59	<0.001	0.57	<0.001
Eosinophils CIBERSORT NatMethods.2015 PMID.25822800	0.5	<0.001	0.42	<0.001
IgG Rody BreastCancerResearch.2008 PMID.19272155	0.51	<0.001	0.41	<0.001
Tcells.Survival.2gene Petitprez Nature.2020 PMID.31942077	0.5	<0.001	0.49	<0.001
Immune.14 Perez JCO.2015 PMID.2560586	0.45	<0.001	0.45	<0.001
Dendritic.cells Bindea Immunity.2013 PMID.24138885	0.48	<0.001	0.46	<0.001
Bcells.ImmuneProfiles.Mouse.Human Shay PNAS.2013 PMID.23382184	0.38	<0.001	0.39	<0.001
Tcells.Th17.cells Charoentong CellRep.2017 PMID.28052254	0.44	<0.001	0.33	<0.001
PDCD1.Single.Gene Pare AnnOncol.2019 PMID.30165419	0.52	<0.001	0.45	<0.001
NK.ImmuneProfiles.Mouse.Human Shay PNAS.2013 PMID.23382184	0.39	<0.001	0.40	<0.001
IFNa.Module10 Gatza ProcNatlAcadSciUSA.2010 PMID.20335537	0.44	<0.001	0.39	<0.001
Mast.cells.Resting CIBERSORT NatMethods.2015 PMID.25822800	0.38	<0.001	0.39	<0.001
Mast.cell Charoentong CellRep.2017 PMID.28052254	0.42	<0.001	0.38	<0.001
TLS.High.In.No.Response Helmink Nature.2020 PMID.31942075	-0.37	<0.001	-0.26	<0.001
Macrophages.M2 Ghassabeh Blood.2006 PMID.16556895	0.43	<0.001	0.40	<0.001
Neutrophils CIBERSORT NatMethods.2015 PMID.25822800	0.41	<0.001	0.39	<0.001
Granulocytes.ImmuneProfiles.Mouse.Human Shay PNAS.2013 PMID.23382184	0.48	<0.001	0.53	<0.001
Influenza.11genes Khatri Immunity.2015 PMID.26682989	0.43	<0.001	0.37	<0.001
MHC.24genes Forero CancerImmunolRes.2016 PMID.26980599	0.36	<0.001	0.35	<0.001
IFN.Pathway ImSig.Nirmal CancerImmunolRes.2018 PMID.30266715	0.42	<0.001	0.34	<0.001
Bcells.Extended Garber CellMolGastroenterolHepatol.2017 PMID.28508029	0.39	<0.001	0.34	<0.001
Mast.cells.Activated CIBERSORT NatMethods.2015 PMID.25822800	0.35	<0.001	0.37	<0.001
Macrophages Bindea Immunity.2013 PMID.24138885	0.47	<0.001	0.48	<0.001
MDSC.Tumor.Macrophages Schlecker JImmunol.2012 PMID.23152559	0.37	<0.001	0.37	<0.001
Monocytes Charoentong CellRep.2017 PMID.28052254	0.29	<0.001	0.30	0.001

© 2023 Fernandez-Martinez A et al. *JAMA Oncology.*

Tcells.CD4.Effector.Memory Charoentong CellRep.2017 PMID.28052254	0.41	<0.001	0.36	<0.001
Neutrophils.Activated.Lung Janiszewska NatCellBiol.2019 PMID.31263265	0.33	<0.001	0.40	<0.001
Tcells.Central.Memory Bindea Immunity.2013 PMID.24138885	0.29	<0.001	0.26	0.003
Tcells.Follicular.Helper Bindea Immunity.2013 PMID.24138885	0.34	<0.001	0.36	<0.001
Tcells.Follicular.Helper TCGA Immunity.2018 PMID.29628290	0.34	<0.001	0.36	<0.001
Neutrophils.Activated.Blood Janiszewska NatCellBiol.2019 PMID.31263265	0.33	<0.001	0.28	0.002
Neutrophils ImSig.Nirmal CancerImmunolRes.2018 PMID.30266715	0.38	<0.001	0.38	<0.001
Bcells.Naive Dybaer JCO.2015 PMID.25800755	0.28	<0.001	0.23	0.008
Tcells.CD4.Central.Memory Charoentong CellRep.2017 PMID.28052254	0.25	<0.001	0.32	<0.001
Tcells.CD8.Exhausted.vs.AntiPDL1.2 Pauken Science.2016 PMID.27789795	0.29	0.001	0.23	0.008
NK.MCP Helmink Nature.2020 PMID.31942077	0.21	0.001	0.13	0.14
TGFB.score TCGA Immunity.2018 PMID.29628290	-0.22	0.001	-0.10	0.25
Tcells.CD8 Bindea Immunity.2013 PMID.24138885	0.24	0.001	0.19	0.03
Tcells.Follicular.Helper Charoentong CellRep.2017 PMID.28052254	0.27	0.001	0.31	<0.001
Bcells.IL10.Minus Lin JImmunol.2014 PMID.25080484	0.34	0.002	0.28	0.001
Immune.IFN TCGA.BRCA.1198 JCI.2020 PMID.32573490	0.28	0.002	0.23	0.01
IFN.Cluster Fan BMCMedGenomics.2011 PMID.21214954	0.27	0.003	0.24	0.007
NK.CD56bright Bindea Immunity.2013 PMID.24138885	0.26	0.003	0.21	0.02
NK.Tcell Charoentong CellRep.2017 PMID.28052254	0.36	0.003	0.37	<0.001
IFN.3.ImmLandscape Wolf PlosOne.2014 PMID.24516633	0.27	0.004	0.25	0.004
IFN.Score.module3 TCGA Immunity.2018 PMID.29628290	0.27	0.004	0.25	0.004
MDSC.Tumor Schlecker JImmunol.2012 PMID.23152559	0.33	0.004	0.30	0.001
Tcells.Regulatory.cell.2gene Petitprez Nature.2020 PMID.31942077	0.31	0.007	0.34	<0.001
Immunosuppression Petitprez Nature.2020 PMID.31942077	-0.23	0.009	-0.14	0.13
Bcells.Memory Dybaer JCO.2015 PMID.25800755	0.23	0.01	0.20	0.02
Tcells.Th2 Charoentong CellRep.2017 PMID.28052254	0.21	0.01	0.22	0.01
Tcells.CD8.Exhausted.vs.Naive.2 Pauken Science.2016 PMID.27789795	0.22	0.01	0.17	0.05

© 2023 Fernandez-Martinez A et al. *JAMA Oncology.*

NK Charoentong CellRep.2017 PMID.28052254	0.25	0.01	0.24	0.006
Bcells.Memory Charoentong CellRep.2017 PMID.28052254	0.22	0.02	0.16	0.08
Neutrophils Bindea Immunity.2013 PMID.24138885	0.24	0.03	0.24	0.006
IFN Rody BreastCancerResearch.2008 PMID.19272155	0.21	0.03	0.21	0.02
Bcells.Centroblast Dybaer JCO.2015 PMID.25800755	0.19	0.03	0.12	0.20
Tcells.Effector.Memory Bindea Immunity.2013 PMID.24138885	0.14	0.03	0.22	0.01
Hematopoietic.Stem.cells.ImmuneProfiles.Mouse.Human Shay PNAS.2013 PMID.23382184	-0.14	0.04	-0.17	0.06
Proliferation.Pathway ImSig.Nirmal CancerImmunolRes.2018 PMID.30266715	0.2	0.04	0.11	0.22
CD103.Negative Broz CancerCell.2014 PMID.25446897	0.2	0.04	0.21	0.02
Tcells.CD8.Effector.vs.Naive.2 Pauken Science.2016 PMID.27789795	0.19	0.04	0.09	0.32

Only the immune signatures significantly associated with TILs as continuous variables in each study are shown. Spearman's correlation coefficients and p-values (95% CI) are included. P-values are adjusted for multiple testing using a Benjamini & Hochberg method to control the False Discovery Rate.

© 2023 Fernandez-Martinez A et al. *JAMA Oncology.*

eTable 4. Association of Tumor-Infiltrating Lymphocytes (TILs) and Immune Gene Expression Signatures (iGES) With Pathologic Complete Response (pCR) in the Combined CALGB 40601 and PAMELA Data Set

All Signatures	OR	Lower CI	Upper CI	p value	AIC
Bcells.Plasma.cells.52genes Miller GenomeBiol.2013 PMID.23618380	1.93	1.51	2.51	<0.001	453.15
Ig TCGA.BRCA.1198 Cell.2015 PMID.26451490	1.94	1.51	2.51	<0.001	453.11
Plasma.cells ImSig.Nirmal CancerImmunolRes.2018 PMID.30266715	2.01	1.57	2.63	<0.001	450.06
IgG Rody BreastCancerResearch.2008 PMID.19272155	1.81	1.42	2.33	<0.001	458.11
Immune1 TCGA.BRCA.1198 JCI.2020 PMID.32573490	1.77	1.40	2.27	<0.001	459.16
IGG.Cluster Fan BMCMedGenomics.2011 PMID.21214954	1.64	1.30	2.09	0.001	464.74
NK.CD56dim Bindea Immunity.2013 PMID.24138885	1.65	1.31	2.12	0.001	464.57
Bcells.Plasma.cells.Metagene Miller GenomeBiol.2013 PMID.23618380	1.65	1.30	2.11	0.001	464.76
TLS.High.In.Response.MCP Helmink Nature.2020 PMID.31942075	1.64	1.30	2.10	0.001	465.05
Immune.CTLA4.CXCL.FOXP3 TCGA.BRCA.1198 JCI.2020 PMID.32573490.	1.63	1.28	2.10	0.002	466.12
CD274.Single.Gene Hollern Cell.2019 PMID.31730857	1.55	1.23	1.98	0.005	468.41
Bcells.Centroblast Dybkae JCO.2015 PMID.25800755	1.55	1.23	1.98	0.005	468.37
Denditic.cells.ImmuneProfiles.Mouse.Human Shay PNAS.2013 PMID.23382184	1.48	1.18	1.88	0.02	471.30
TILs 40	2.29	1.40	3.77	0.02	471.68
TILs	1.01	1.01	1.02	0.02	472.23
Macrophages.M1 CIBERSORT NatMethods.2015 PMID.25822800	1.47	1.16	1.86	0.02	472.10
STAT1 Rody BreastCancerResearch.2008 PMID.19272155	1.46	1.16	1.86	0.02	472.11
IFN.5.ImmLandscape Wolf PlosOne.2014 PMID.24516633	1.44	1.15	1.83	0.02	472.63
Bcells.Plasmablast Dybaer JCO.2015 PMID.25800755	1.43	1.14	1.80	0.02	472.66
Tcells.CD4.Memory.Activated CIBERSORT NatMethods.2015 PMID.25822800	1.44	1.14	1.82	0.02	472.81
Stromal.Inflammation Heng JPathol.2017 PMID.27861902	1.43	1.14	1.80	0.03	473.18
Bcells.Cluster Iglesia CCR.2014 PMID.24916698	1.40	1.12	1.77	0.03	473.77

© 2023 Fernandez-Martinez A et al. *JAMA Oncology.*

Dendritic.cells.Activated Bindea Immunity.2013 PMID.24138885	1.41	1.12	1.79	0.03	473.91
Immune.Suppression Kardos JCIInsight.2016 PMID.27699256	1.41	1.12	1.79	0.03	474.03
Proliferation.Pathway ImSig.Nirmal CancerImmunolRes.2018 PMID.30266715	1.41	1.12	1.78	0.03	473.78
Tcells.CD8.Effector.vs.Naive.2 Pauken Science.2016 PMID.27789795	1.40	1.12	1.78	0.03	473.84
Tcells.CD8.Exhausted.vs.Naive.2 Pauken Science.2016 PMID.27789795	1.40	1.12	1.78	0.03	473.88
MDSC Charoentong CellRep.2017 PMID.28052254	1.41	1.12	1.79	0.03	474.17
Plasma.cells CIBERSORT NatMethods.2015 PMID.25822800	1.38	1.11	1.74	0.03	474.31
TLS.12genes.Chemokine Zhu FrontImmunol.2017 PMID.28713385	1.39	1.11	1.76	0.03	474.40
Tcells.CD4.Activated Charoentong CellRep.2017 PMID.28052254	1.39	1.11	1.77	0.03	474.35
TILs 20	1.86	1.20	2.91	0.04	474.98
Immune.Active Hollern Cell.2019 PMID.31730857	1.37	1.09	1.73	0.04	475.04
Dendritic.cells.Activated CIBERSORT NatMethods.2015 PMID.25822800	1.37	1.09	1.74	0.04	475.31
PDCD1.Single.Gene Pare AnnOncol.2019 PMID.30165419	1.39	1.10	1.78	0.04	475.19
Cytotoxic.Lymphocytes.MCP Petitprez Nature.2020 PMID.31942077	1.37	1.09	1.73	0.04	475.18
Tcells.Activation Petitprez Nature.2020 PMID.31942077	1.38	1.09	1.77	0.04	475.16
MHC.I.CoreGenes Lauss NatCommun.2017 PMID29170503	1.36	1.08	1.71	0.04	475.56
MERCK.Immune.Signature Cristescu Science.2018 PMID.30309915	1.36	1.09	1.73	0.04	475.44
Tcells.CD8.Exhausted.vs.AntiPDL1.2 Pauken Science.2016 PMID.27789795	1.35	1.08	1.70	0.05	475.70
Tcells.Cluster Iglesia CCR.2014 PMID.24916698	1.35	1.08	1.70	0.05	475.80
Tcells.Th1.cells Bindea Immunity.2013 PMID.24138885	1.34	1.07	1.68	0.06	476.16
Tcells.CD8.Memory.vs.Naive.Metagene.3 Pauken Science.2016 PMID.27789795	1.34	1.07	1.70	0.06	476.18
Monocytes Charoentong CellRep.2017 PMID.28052254	1.34	1.07	1.68	0.06	476.30
Tcells.Central.Memory Bindea Immunity.2013 PMID.24138885	1.32	1.06	1.66	0.07	476.47
Tcells.Th17.cells Charoentong CellRep.2017 PMID.28052254	1.32	1.06	1.66	0.07	476.47
Immune.CD19 TCGA.BRCA.1198 JCI.2020 PMID.32573490	1.32	1.06	1.66	0.07	476.68
Tcells.Follicular.Helper Bindea Immunity.2013 PMID.24138885	1.33	1.06	1.68	0.07	476.67

© 2023 Fernandez-Martinez A et al. *JAMA Oncology.*

Tcells.Follicular.Helper TCGA Immunity.2018 PMID.29628290	1.33	1.06	1.68	0.07	476.67
TLS.High.In.No.Response Helmink Nature.2020 PMID.31942075	0.76	0.61	0.95	0.07	476.90
Mast.cells Bindea Immunity.2013 PMID.24138885	0.77	0.61	0.95	0.08	476.97
Macrophages.M0 CIBERSORT NatMethods.2015 PMID.25822800	1.31	1.05	1.64	0.08	477.07
MHC.11genes Forero CancerImmunolRes.2016 PMID.26980599	1.30	1.04	1.63	0.08	477.42
NK.Activated CIBERSORT NatMethods.2015 PMID.25822800	1.30	1.04	1.63	0.08	477.42
CTLA4.Single.Gene Hollern Cell.2019 PMID.31730857	1.32	1.04	1.69	0.08	477.31
Tcells.CD8 CIBERSORT NatMethods.2015 PMID.25822800	1.30	1.04	1.63	0.08	477.45
Tcells.Follicular.Helper CIBERSORT NatMethods.2015 PMID.25822800	1.30	1.04	1.64	0.08	477.44
CTLA4.Pathway GSEA.BIOCARTA ProcNatlAcadSciUSA.2005 PMID.16199517	1.31	1.04	1.65	0.08	477.17
Immune.87 Perez JCO.2015 PMID.2560586	1.30	1.04	1.64	0.08	477.24
Tcells.CD8.Activated Charoentong CellRep.2017 PMID.28052254	1.30	1.04	1.64	0.08	477.34
Tcells.MCP Petitprez Nature.2020 PMID.31942077	1.30	1.04	1.65	0.08	477.38
Immune.CD8.GZMK TCGA.BRCA.1198 JCI.2020 PMID.32573490	1.30	1.04	1.63	0.08	477.33
Tcells.Effector.Memory Bindea Immunity.2013 PMID.24138885	0.78	0.62	0.97	0.08	477.50
CD8.Cluster Iglesia CCR.2014 PMID.24916698	1.29	1.03	1.62	0.09	477.64
Bcells.Centrocyte Dybkaer JCO.2015 PMID.25800755	1.28	1.02	1.61	0.10	478.00
Bcells.Memory CIBERSORT NatMethods.2015 PMID.25822800	1.27	1.02	1.60	0.10	478.03
Bcells.Activated Charoentong CellRep.2017 PMID.28052254	1.28	1.02	1.60	0.10	477.98
Plasmacytoid.Dendritic.cell Charoentong CellRep.2017 PMID.28052254	0.78	0.62	0.98	0.10	477.92
MDSC.Tumor.Macrophages Schlecker JImmunol.2012 PMID.23152559	1.29	1.02	1.63	0.10	478.03
Cytotoxic.cells Bindea Immunity.2013 PMID.24138885	1.28	1.02	1.60	0.10	477.99
Tcells.CD8.Central.Memory Charoentong CellRep.2017 PMID.28052254	0.79	0.63	0.98	0.10	478.03
Tcells.Th2 Charoentong CellRep.2017 PMID.28052254	1.27	1.02	1.60	0.10	478.03
TLS.Tumors.w.TLS.and.CD8.vs.CD8.alone Cabrita Nature.2020 PMID.31942071	1.27	1.02	1.60	0.10	478.03

© 2023 Fernandez-Martinez A et al. *JAMA Oncology.*

MDSC.Granulocytic Youn LeukocBiol.2012 PMID.21954284	1.27	1.02	1.60	0.10	478.07
Tcells.Gamma.Delta CIBERSORT NatMethods.2015 PMID.25822800	1.27	1.02	1.59	0.10	478.15
MHC.II Rody BreastCancerResearch.2008 PMID.19272155	1.27	1.02	1.60	0.10	478.20
Immune.CD34.TIE1 TCGA.BRCA.1198 JCI.2020 PMID.32573490	0.80	0.64	0.99	0.11	478.37
Tcells.Survival.2gene Petitprez Nature.2020 PMID.31942077	1.27	1.01	1.60	0.11	478.36
Tcells.NK.51genes Miller GenomeBiol.2013 PMID.23618380	1.26	1.01	1.58	0.11	478.44
Bcells ImSig.Nirmal CancerImmunolRes.2018 PMID.30266715	1.26	1.01	1.58	0.11	478.48
PD1.Signaling.Reactome GSEA ProcNatlAcadSciUSA.2005 PMID.16199517	1.26	1.01	1.59	0.11	478.51
Tcells.Bcells.Lymphocyte.Infiltration Calabro BreastCancerResTreat.2009 PMID.18592372	1.25	1.01	1.57	0.12	478.59
Lymphocyte.Infiltration.Expression.Score TCGA Immunity.2018 PMID.29628290	1.25	1.01	1.57	0.12	478.59
Bcells.Memory Charoentong CellRep.2017 PMID.28052254	1.25	1.00	1.57	0.12	478.66
Macrophages Charoentong CellRep.2017 PMID.28052254	0.80	0.64	0.99	0.12	478.58
NK.CD56bright Bindea Immunity.2013 PMID.24138885	1.25	1.00	1.57	0.12	478.64
MHC.I Rody BreastCancerResearch.2008 PMID.19272155	1.26	1.00	1.58	0.12	478.75
Tcells.CD8.Effector.Memory Charoentong CellRep.2017 PMID.28052254	1.25	1.00	1.57	0.12	478.75
NK.Resting CIBERSORT NatMethods.2015 PMID.25822800	1.25	1.00	1.57	0.12	478.79
TILs 60	1.73	0.99	3.04	0.12	478.89
Bcells.Tcells.Cooperation Hollern Cell.2019 PMID.31730857	1.24	1.00	1.55	0.12	478.86
MCD3.CD8 Fan BMCMedGenomics.2011 PMID.21214954	1.24	1.00	1.56	0.12	478.89
Tcells.CD8.Memory.vs.Naive.Metagene.2 Pauken Science.2016 PMID.27789795	1.24	1.00	1.56	0.12	478.88
Tcells.Resident.Memory.Single.cell Savas NatMed.2018 PMID.29942092	1.25	1.00	1.57	0.12	478.91
NK.CD56dim Charoentong CellRep.2017 PMID.28052254	1.23	0.99	1.54	0.13	479.07
NK Charoentong CellRep.2017 PMID.28052254	0.81	0.65	1.01	0.13	479.13
Tcells.Th2.cells Bindea Immunity.2013 PMID.24138885	1.23	0.99	1.54	0.13	479.12

© 2023 Fernandez-Martinez A et al. *JAMA Oncology.*

Bcells.ImmuneProfiles.Mouse.Human Shay PNAS.2013 PMID.23382184	1.23	0.98	1.53	0.14	479.29
Bcells.Immature Charoentong CellRep.2017 PMID.28052254	1.23	0.98	1.53	0.14	479.32
Immune.HLA.A.F TCGA.BRCA.1198 JCI.2020 PMID.32573490	1.23	0.98	1.55	0.14	479.28
Bcells.Memory Dybaer JCO.2015 PMID.25800755	1.23	0.98	1.54	0.14	479.31
Macrophages.Th1.Cluster Iglesia CCR.2014 PMID.24916698	1.23	0.98	1.54	0.15	479.37
Immune.CD4.CD53.CD84.BTK TCGA.BRCA.1198 JCI.2020 PMID.32573490	1.22	0.98	1.54	0.15	479.42
Immune.Hot.vs.Cold.CD8 Cabrita Nature.2020 PMID.31942071	1.22	0.98	1.54	0.15	479.45
LCK Rody BreastCancerResearch.2008 PMID.19272155	1.22	0.98	1.53	0.15	479.50
Tcells.CD8.Exhausted.vs.Naive.Metagene.1 Pauken Science.2016 PMID.27789795	1.22	0.98	1.54	0.15	479.48
Tcells.Bcell.Cluster Fan BMCMedGenomics.2011 PMID.21214954	1.22	0.98	1.54	0.16	479.55
Tcells Bindea Immunity.2013 PMID.24138885	1.22	0.98	1.54	0.16	479.56
Bcells.Naive CIBERSORT NatMethods.2015 PMID.25822800	1.21	0.98	1.52	0.16	479.61
Tcells.Gamma.Delta Bindea Immunity.2013 PMID.24138885	1.21	0.98	1.51	0.16	479.63
Tcells.Gamma.Delta TCGA Immunity.2018 PMID.29628290	1.21	0.98	1.51	0.16	479.63
Tcells ImSig.Nirmal CancerImmunolRes.2018 PMID.30266715	1.21	0.97	1.52	0.16	479.68
Tcells.CD8.Exhausted.at.day.8.post.Imm.vs.Naive.Metagene.1 Pauken Science.2016 PMID.27789795	1.22	0.97	1.53	0.16	479.71
Bcells Bindea Immunity.2013 PMID.24138885	1.20	0.96	1.50	0.19	479.95
Tcells.NK.Metagene Miller GenomeBiol.2013 PMID.23618380	1.20	0.96	1.50	0.20	480.03
Macrophages.Monocytes.CSF1.Response Beck CCR.2009 PMID..19188147	1.20	0.96	1.50	0.20	480.09
CSF1.Response TCGA Immunity.2018 PMID.29628290	1.20	0.96	1.50	0.20	480.09
Neutrophils.Activated.Lung Janiszewska NatCellBiol.2019 PMID.31263265	1.20	0.96	1.51	0.20	480.05
Tcells.CD4.Memory.Resting CIBERSORT NatMethods.2015 PMID.25822800	1.20	0.96	1.50	0.20	480.08
Tcells.Th1.cells Charoentong CellRep.2017 PMID.28052254	1.20	0.96	1.50	0.20	480.10
NK ImSig.Nirmal CancerImmunolRes.2018 PMID.30266715	1.19	0.96	1.49	0.20	480.17

© 2023 Fernandez-Martinez A et al. *JAMA Oncology.*

Tcells.CD8.Exhausted.vs.Naive.Metagene.3 Pauken Science.2016 PMID.27789795	1.20	0.96	1.50	0.20	480.17
Tcells.CD8.MCP Petitprez Nature.2020 PMID.31942077	1.20	0.96	1.51	0.20	480.19
Tcells.Regulatory.Tregs CIBERSORT NatMethods.2015 PMID.25822800	1.19	0.95	1.49	0.20	480.21
Tcells.CD4.Naive CIBERSORT NatMethods.2015 PMID.25822800	1.19	0.95	1.49	0.22	480.31
Neutrophils ImSig.Nirmal CancerImmunolRes.2018 PMID.30266715	1.19	0.95	1.51	0.22	480.32
Dendritic.cells.Immature. Bindea Immunity.2013 PMID.24138885	1.18	0.95	1.48	0.22	480.38
MDSC.Neutrophil Youn LeukocBiol.2012 PMID.21954284	1.19	0.95	1.49	0.22	480.38
Macrophages ImSig.Nirmal CancerImmunolRes.2018 PMID.30266715	1.18	0.95	1.48	0.22	480.42
Immune.Cell.Content Verhaak NatCommun.2013 PMID.24113773	1.18	0.95	1.48	0.22	480.43
IFNg.Module11 Gatza ProcNatlAcadSciUSA.2010 PMID.20335537	1.18	0.95	1.48	0.23	480.47
Influenza.11genes Khatri Immunity.2015 PMID.26682989	1.18	0.95	1.47	0.23	480.47
Bcells.IL10.Plus Lin JImmunol.2014 PMID.25080484	1.18	0.94	1.48	0.23	480.49
TLS.Known.Markers Cabrita Nature.2020 PMID.31942071	1.17	0.94	1.47	0.24	480.56
Neutrophils.Activated.Blood Janiszewska NatCellBiol.2019 PMID.31263265	0.85	0.67	1.06	0.24	480.57
TLS.Hallmark Cabrita Nature.2020 PMID.31942071	1.17	0.94	1.47	0.24	480.61
Monocytes CIBERSORT NatMethods.2015 PMID.25822800	1.17	0.94	1.46	0.24	480.62
Dendritic.cells.Resting CIBERSORT NatMethods.2015 PMID.25822800	1.16	0.94	1.45	0.26	480.75
Tcells.Regulatory Charoentong CellRep.2017 PMID.28052254	1.17	0.93	1.46	0.26	480.77
Immune.HLA.D TCGA.BRCA.1198 JCI.2020 PMID.32573490	1.17	0.93	1.46	0.26	480.78
Monocytes..Dendritic.cell.Metagene Miller GenomeBiol.2013 PMID.23618380	1.17	0.93	1.46	0.26	480.80
Tcells.CD8.Memory.vs.Naive.1 Pauken Science.2016 PMID.27789795	1.17	0.93	1.47	0.26	480.81
Tcells.CD8.Memory.vs.Naive.Metagene.1 Pauken Science.2016 PMID.27789795	1.17	0.93	1.47	0.26	480.81
Cytolytic.Activity Rooney Cell.2015 PMID.25594174	1.16	0.93	1.46	0.27	480.87
Monocytes.Dendritic.25genes Miller GenomeBiol.2013 PMID.23618380	1.15	0.92	1.44	0.32	481.11

© 2023 Fernandez-Martinez A et al. *JAMA Oncology.*

IFN.Cluster.GSEA.GP11 Fan BMCMedGenomics.2011 PMID.21214954	1.15	0.92	1.43	0.32	481.14
Tcells.CD8 Bindea Immunity.2013 PMID.24138885	0.87	0.70	1.09	0.32	481.14
Neutrophils Bindea Immunity.2013 PMID.24138885	0.88	0.70	1.09	0.34	481.25
Tcells.CD4.Central.Memory Charoentong CellRep.2017 PMID.28052254	0.88	0.71	1.09	0.35	481.29
Neutrophils CIBERSORT NatMethods.2015 PMID.25822800	1.13	0.91	1.42	0.35	481.33
Dendritic.cells Bindea Immunity.2013 PMID.24138885	1.13	0.91	1.42	0.36	481.35
Bcells.IL10.Minus Lin JImmunol.2014 PMID.25080484	1.13	0.91	1.41	0.39	481.46
Serum.Response.Up TCGA Immunity.2018 PMID.29628290	1.13	0.90	1.41	0.40	481.54
Bcells Garber CellMolGastroenterolHepatol.2017 PMID.28508029	1.12	0.90	1.39	0.41	481.57
NK.MCP Helmink Nature.2020 PMID.31942077	1.12	0.90	1.39	0.41	481.60
Tcells.Bcell.KEGG.hematopoietic.cell.lineage GSEA.GP2 ProcNatlAcadSciUSA.2005 PMID.16199517	1.12	0.90	1.40	0.41	481.59
Macrophages.M2 CIBERSORT NatMethods.2015 PMID.25822800	1.12	0.90	1.40	0.42	481.62
Tcells.CD4.Effector.Memory Charoentong CellRep.2017 PMID.28052254	0.90	0.72	1.12	0.43	481.66
IFNa.Module10 Gatza ProcNatlAcadSciUSA.2010 PMID.20335537	1.12	0.90	1.39	0.43	481.67
Wound.Healing Chang PlosBiol.2004 PMID.14737219	1.11	0.89	1.39	0.44	481.72
Macrophages Bindea Immunity.2013 PMID.24138885	1.11	0.89	1.39	0.44	481.72
Granulocytes.ImmuneProfiles.Mouse.Human Shay PNAS.2013 PMID.23382184	1.11	0.89	1.40	0.46	481.77
MDSC.Tumor Schlecker JImmunol.2012 PMID.23152559	1.11	0.89	1.39	0.46	481.80
Tcells.ImmuneProfiles.Mouse.Human Shay PNAS.2013 PMID.23382184	1.10	0.89	1.37	0.48	481.84
Tcells.CD8.Exhausted.Anti.PDL1.vs.Control.Metagene.1 Pauken Science.2016 PMID.27789795	1.10	0.89	1.38	0.48	481.86
Tcells.Thelper Bindea Immunity.2013 PMID.24138885	1.10	0.88	1.37	0.49	481.88
HCK Rody BreastCancerResearch.2008 PMID.19272155	1.10	0.88	1.38	0.49	481.90
NK.ImmuneProfiles.Mouse.Human Shay PNAS.2013 PMID.23382184	1.10	0.88	1.36	0.50	481.93

© 2023 Fernandez-Martinez A et al. *JAMA Oncology.*

Bcells.Extended Garber CellMolGastroenterolHepatol.2017 PMID.28508029	0.91	0.73	1.14	0.50	481.94
Dendritic.cells.Activated Charoentong CellRep.2017 PMID.28052254	1.09	0.88	1.37	0.52	481.98
Mast.cells.Resting CIBERSORT NatMethods.2015 PMID.25822800	1.08	0.87	1.35	0.57	482.10
Mast.cell Charoentong CellRep.2017 PMID.28052254	0.93	0.74	1.15	0.58	482.13
Tcells.Th17.cells Bindea Immunity.2013 PMID.24138885	0.93	0.75	1.15	0.61	482.19
Immune.14 Perez JCO.2015 PMID.2560586	1.07	0.86	1.33	0.65	482.27
IFN Rody BreastCancerResearch.2008 PMID.19272155	0.94	0.75	1.16	0.65	482.26
MHC.24genes Forero CancerImmunolRes.2016 PMID.26980599	0.94	0.75	1.17	0.65	482.27
Macrophages.ImmuneProfiles.Mouse.Human Shay PNAS.2013 PMID.23382184	1.06	0.85	1.33	0.68	482.32
Immunosuppression Petitprez Nature.2020 PMID.31942077	0.95	0.76	1.18	0.71	482.37
Hematopoietic.Stem.cells.ImmuneProfiles.Mouse.Human Shay PNAS.2013 PMID.23382184	1.06	0.85	1.32	0.71	482.37
Neutrophils Charoentong CellRep.2017 PMID.28052254	0.95	0.75	1.18	0.71	482.37
Tcells.Follicular.Helper Charoentong CellRep.2017 PMID.28052254	1.06	0.85	1.32	0.71	482.36
Eosinophils Charoentong CellRep.2017 PMID.28052254	1.06	0.85	1.32	0.71	482.38
Tcells.Gamma.Delta Charoentong CellRep.2017 PMID.28052254	1.05	0.85	1.31	0.71	482.39
NK Bindea Immunity.2013 PMID.24138885	0.95	0.77	1.18	0.73	482.41
Tcells.Regulatory.cell.2gene Petitprez Nature.2020 PMID.31942077	0.95	0.76	1.19	0.73	482.41
Immune.GIMAP.IL16 TCGA.BRCA.1198 JCI.2020 PMID.32573490	1.05	0.84	1.31	0.73	482.42
NK.CD56bright Charoentong CellRep.2017 PMID.28052254	1.05	0.84	1.31	0.76	482.46
Bcells.Naive Dybaer JCO.2015 PMID.25800755	0.97	0.78	1.20	0.83	482.52
IFN.Pathway ImSig.Nirmal CancerImmunolRes.2018 PMID.30266715	1.03	0.83	1.29	0.83	482.53
Immune.FOS.JUN.IL6 TCGA.BRCA.1198 JCI.2020 PMID.32573490	0.97	0.77	1.21	0.83	482.53
Macrophages.M2 Ghassabeh Blood.2006 PMID.16556895	1.03	0.83	1.29	0.83	482.52
Monocytes ImSig.Nirmal CancerImmunolRes.2018 PMID.30266715	1.03	0.83	1.30	0.83	482.53

© 2023 Fernandez-Martinez A et al. *JAMA Oncology.*

IFN.3.ImmLandscape Wolf PlosOne.2014 PMID.24516633	0.97	0.78	1.21	0.87	482.56
IFN.Score.module3 TCGA Immunity.2018 PMID.29628290	0.97	0.78	1.21	0.87	482.56
NK.Tcell Charoentong CellRep.2017 PMID.28052254	1.03	0.82	1.28	0.87	482.57
Dendritic.cells.Immature. Charoentong CellRep.2017 PMID.28052254	1.02	0.82	1.27	0.88	482.58
IFN.Cluster Fan BMCMedGenomics.2011 PMID.21214954	0.98	0.79	1.21	0.88	482.58
Mast.cells.Activated CIBERSORT NatMethods.2015 PMID.25822800	1.02	0.82	1.27	0.88	482.57
Immune.IFN TCGA.BRCA.1198 JCI.2020 PMID.32573490	0.98	0.79	1.22	0.88	482.58
Eosinophils Bindea Immunity.2013 PMID.24138885	1.02	0.82	1.27	0.90	482.59
CD103.Negative Broz CancerCell.2014 PMID.25446897	1.01	0.81	1.26	0.95	482.61
CD68.Cluster Iglesia CCR.2014 PMID.24916698	0.99	0.80	1.23	0.96	482.61
Neutrophils.MCP Petitprez Nature.2020 PMID.31942077	0.99	0.80	1.24	0.97	482.61
TLS.9genes Cabrita Nature.2020 PMID.31942071	1.00	0.81	1.25	0.98	482.62
TGFB.score TCGA Immunity.2018 PMID.29628290	1.00	0.81	1.25	0.99	482.62
Eosinophils CIBERSORT NatMethods.2015 PMID.25822800	1.00	0.81	1.24	0.99	482.62

Logistic regression multivariable models adjusted by study and treatment arm has been built for each biomarker. P-values are adjusted for multiple testing using a Benjamini & Hochberg method to control the False Discovery Rate.

© 2023 Fernandez-Martinez A et al. *JAMA Oncology.*

eTable 5. Association of Tumor-Infiltrating Lymphocytes (TILs) and Immune Gene Expression Signatures (iGES) With Pathologic Complete Response (pCR) in the Presence of Clinical Parameters Using the Combined CALGB 40601 and PAMELA Cohort

Signatures	OR	Lower CI	Upper CI	p value	AIC
Bcells.Plasma.cells.52genes Miller GenomeBiol.2013 PMID.23618380	1.82	1.39	2.40	0.002	429.72
Ig TCGA.BRCA.1198 Cell.2015 PMID.26451490	1.80	1.38	2.37	0.002	420.23
Plasma.cells ImSig.Nirmal CancerImmunolRes.2018 PMID.30266715	1.82	1.40	2.42	0.002	429.49
IgG Rody BreastCancerResearch.2008 PMID.19272155	1.67	1.29	2.19	0.005	434.08
Immune1 TCGA.BRCA.1198 JCI.2020 PMID.32573490	1.66	1.29	2.17	0.005	434.23
IGG.Cluster Fan BMCMedGenomics.2011 PMID.21214954	1.55	1.21	2.01	0.02	438.13
NK.CD56dim Bindea Immunity.2013 PMID.24138885	1.53	1.18	2.00	0.03	439.31
Bcells.Plasma.cells.Metagene Miller GenomeBiol.2013 PMID.23618380	1.54	1.20	2.01	0.03	438.43
TLS.High.In.Response.MCP Helmink Nature.2020 PMID.31942075	1.57	1.22	2.05	0.02	437.57
TILs	1.01	1.00	1.02	0.18	444.48

Logistic regression multivariable models are adjusted by study, treatment arm, clinical stage, age, HR-status, menopausal status, and subtype.
Only the iGES significantly associated with pCR and TILs are shown.
P-values are adjusted for multiple testing using a Benjamini & Hochberg method to control the False Discovery Rate.

© 2023 Fernandez-Martinez A et al. *JAMA Oncology.*

eTable 6. Accuracy Metrics (Area Under the Curve From the Receiver Operating Characteristic Curves: AUC ROC) of the Univariable Immune-Biomarker Models to Predict Pathologic Complete Response (pCR)

Signature	AUC CALGB 40601	AUC PAMELA
Ig TCGA.BRCA.1198 Cell.2015 PMID.26451490	0.64	0.65
Immune.CTLA4.CXCL.FOXP3 TCGA.BRCA.1198 JCI.2020 PMID.32573490.	0.60	0.64
Bcells.Plasma.cells.52genes Miller GenomeBiol.2013 PMID.23618380	0.63	0.64
IgG Rody BreastCancerResearch.2008 PMID.19272155	0.62	0.64
Plasma.cells ImSig.Nirmal CancerImmunolRes.2018 PMID.30266715	0.66	0.63
Immune1 TCGA.BRCA.1198 JCI.2020 PMID.32573490	0.62	0.62
IGG.Cluster Fan BMCMedGenomics.2011 PMID.21214954	0.60	0.62
Bcells.Plasmablast Dybaer JCO.2015 PMID.25800755	0.62	0.59
TILs	0.60	0.57
Monocytes Charoentong CellRep.2017 PMID.28052254	0.62	0.56
Denditic.cells.ImmuneProfiles.Mouse.Human Shay PNAS.2013 PMID.23382184	0.62	0.56
MDSC.Granulocytic Youn LeukocBiol.2012 PMID.21954284	0.61	0.53
TILs 40	0.60	0.59
TLS.High.In.Response.MCP Helmink Nature.2020 PMID.31942075	0.60	0.63
IFN.5.ImmLandscape Wolf PlosOne.2014 PMID.24516633	0.59	0.60
Tcells.CD8.Exhausted.vs.AntiPDL1.2 Pauken Science.2016 PMID.27789795	0.59	0.53
Plasma.cells CIBERSORT NatMethods.2015 PMID.25822800	0.59	0.55
MDSC.Tumor.Macrophages Schlecker JImmunol.2012 PMID.23152559	0.59	0.61
MHC.11genes Forero CancerImmunolRes.2016 PMID.26980599	0.59	0.58
PD1.Signaling.Reactome GSEA ProcNatlAcadSciUSA.2005 PMID.16199517	0.59	0.56
TLS.High.In.No.Response Helmink Nature.2020 PMID.31942075	0.58	0.55
TILs 20	0.58	0.55
Bcells.Centrocyte Dybkaer JCO.2015 PMID.25800755	0.58	0.52
Bcells.Plasma.cells.Metagene Miller GenomeBiol.2013 PMID.23618380	0.58	0.63
Tcells.CD8.Exhausted.at.day.8.post.Imm.vs.Naive.Metagene.1 Pauken Science.2016 PMID.27789795	0.58	0.56
Tcells.CD4.Activated Charoentong CellRep.2017 PMID.28052254	0.58	0.54
Bcells.Centroblast Dybkae JCO.2015 PMID.25800755	0.58	0.63
Macrophages.M0 CIBERSORT NatMethods.2015 PMID.25822800	0.58	0.60
NK.CD56dim Bindea Immunity.2013 PMID.24138885	0.58	0.63
Tcells.CD8.Exhausted.vs.Naive.Metagene.3 Pauken Science.2016 PMID.27789795	0.57	0.57

© 2023 Fernandez-Martinez A et al. *JAMA Oncology.*

Tcells.CD4.Memory.Activated CIBERSORT NatMethods.2015 PMID.25822800	0.57	0.58
CD274.Single.Gene Hollern Cell.2019 PMID.31730857	0.57	0.65
Tcells.Th1.cells Bindea Immunity.2013 PMID.24138885	0.57	0.56
Tcells.CD8.Exhausted.vs.Naive.Metagene.1 Pauken Science.2016 PMID.27789795	0.57	0.53
Tcells.Th17.cells Charoentong CellRep.2017 PMID.28052254	0.57	0.53
Tcells.CD8.Memory.vs.Naive.1 Pauken Science.2016 PMID.27789795	0.57	0.55
MHC.II Rody BreastCancerResearch.2008 PMID.19272155	0.57	0.59
MDSC Charoentong CellRep.2017 PMID.28052254	0.57	0.60
PDCD1.Single.Gene Pare AnnOncol.2019 PMID.30165419	0.57	0.61
Tcells.Activation Petitprez Nature.2020 PMID.31942077	0.57	0.62
Tcells.Central.Memory Bindea Immunity.2013 PMID.24138885	0.57	0.59
TLS.12genes.Chemokine Zhu FrontImmunol.2017 PMID.28713385	0.57	0.60
Immune.CD8.GZMK TCGA.BRCA.1198 JCI.2020 PMID.32573490	0.56	0.56
Tcells.Th2 Charoentong CellRep.2017 PMID.28052254	0.56	0.64
TILs 60	0.56	0.55
Neutrophils CIBERSORT NatMethods.2015 PMID.25822800	0.56	0.55
Dendritic.cells.Activated Bindea Immunity.2013 PMID.24138885	0.56	0.60
Bcells.Memory Dybaer JCO.2015 PMID.25800755	0.56	0.58
STAT1 Rody BreastCancerResearch.2008 PMID.19272155	0.56	0.61
LCK Rody BreastCancerResearch.2008 PMID.19272155	0.56	0.56
Tcells.CD8.Effector.vs.Naive.2 Pauken Science.2016 PMID.27789795	0.56	0.58
MERCK.Immune.Signature Cristescu Science.2018 PMID.30309915	0.56	0.60
CTLA4.Single.Gene Hollern Cell.2019 PMID.31730857	0.56	0.59
Tcells.Cluster Iglesia CCR.2014 PMID.24916698	0.56	0.57
Immune.Suppression Kardos JCIInsight.2016 PMID.27699256	0.56	0.61
Tcells.CD8.Memory.vs.Naive.Metagene.3 Pauken Science.2016 PMID.27789795	0.56	0.57
Immune.87 Perez JCO.2015 PMID.2560586	0.56	0.57
Immune.Active Hollern Cell.2019 PMID.31730857	0.56	0.59
Neutrophils.Activated.Blood Janiszewska NatCellBiol.2019 PMID.31263265	0.56	0.50
Tcells.CD8.Central.Memory Charoentong CellRep.2017 PMID.28052254	0.56	0.54
Tcells.CD8.Activated Charoentong CellRep.2017 PMID.28052254	0.56	0.56
TLS.Known.Markers Cabrita Nature.2020 PMID.31942071	0.56	0.53
Tcells.CD8.Exhausted.vs.Naive.2 Pauken Science.2016 PMID.27789795	0.56	0.59
Stromal.Inflammation Heng JPathol.2017 PMID.27861902	0.56	0.60
MHC.I.CoreGenes Lauss NatCommun.2017 PMID29170503	0.56	0.64
Macrophages.M1 CIBERSORT NatMethods.2015 PMID.25822800	0.55	0.61

© 2023 Fernandez-Martinez A et al. *JAMA Oncology.*

NK ImSig.Nirmal CancerImmunolRes.2018 PMID.30266715	0.55	0.55
Dendritic.cells.Activated CIBERSORT NatMethods.2015 PMID.25822800	0.55	0.61
CD8.Cluster Iglesia CCR.2014 PMID.24916698	0.55	0.56
Bcells.Extended Garber CellMolGastroenterolHepatol.2017 PMID.28508029	0.55	0.55
NK.Resting CIBERSORT NatMethods.2015 PMID.25822800	0.55	0.56
Bcells.Cluster Iglesia CCR.2014 PMID.24916698	0.55	0.56
Tcells.NK.51genes Miller GenomeBiol.2013 PMID.23618380	0.55	0.58
Tcells.Gamma.Delta CIBERSORT NatMethods.2015 PMID.25822800	0.55	0.56
TLS.Tumors.w.TLS.and.CD8.vs.CD8.alone Cabrita Nature.2020 PMID.31942071	0.55	0.55
Bcells.Activated Charoentong CellRep.2017 PMID.28052254	0.55	0.55
Neutrophils Bindea Immunity.2013 PMID.24138885	0.55	0.53
Hematopoietic.Stem.cells.ImmuneProfiles.Mouse.Human Shay PNAS.2013 PMID.23382184	0.55	0.45
TLS.Hallmark Cabrita Nature.2020 PMID.31942071	0.55	0.51
NK Charoentong CellRep.2017 PMID.28052254	0.55	0.56
Tcells.Th2.cells Bindea Immunity.2013 PMID.24138885	0.55	0.62
Tcells.CD8.Memory.vs.Naive.Metagene.2 Pauken Science.2016 PMID.27789795	0.55	0.59
Immune.GIMAP.IL16 TCGA.BRCA.1198 JCI.2020 PMID.32573490	0.55	0.53
Tcells.Survival.2gene Petitprez Nature.2020 PMID.31942077	0.55	0.57
Tcells.CD4.Naive CIBERSORT NatMethods.2015 PMID.25822800	0.55	0.53
Bcells ImSig.Nirmal CancerImmunolRes.2018 PMID.30266715	0.55	0.55
NK.CD56bright Bindea Immunity.2013 PMID.24138885	0.55	0.59
Bcells.Memory Charoentong CellRep.2017 PMID.28052254	0.55	0.58
Tcells ImSig.Nirmal CancerImmunolRes.2018 PMID.30266715	0.55	0.56
MCD3.CD8 Fan BMCMedGenomics.2011 PMID.21214954	0.55	0.56
Macrophages Charoentong CellRep.2017 PMID.28052254	0.55	0.52
Bcells.Immature Charoentong CellRep.2017 PMID.28052254	0.54	0.55
Proliferation.Pathway ImSig.Nirmal CancerImmunolRes.2018 PMID.30266715	0.54	0.59
Immune.FOS.JUN.IL6 TCGA.BRCA.1198 JCI.2020 PMID.32573490	0.54	0.50
IFN.Score.module3 TCGA Immunity.2018 PMID.29628290	0.54	0.43
TLS.9genes Cabrita Nature.2020 PMID.31942071	0.54	0.52
Dendritic.cells.Activated Charoentong CellRep.2017 PMID.28052254	0.54	0.60
IFN.Cluster Fan BMCMedGenomics.2011 PMID.21214954	0.54	0.44
Immune.IFN TCGA.BRCA.1198 JCI.2020 PMID.32573490	0.54	0.44
Tcells.CD8.Memory.vs.Naive.Metagene.1 Pauken Science.2016 PMID.27789795	0.54	0.55
CD68.Cluster Iglesia CCR.2014 PMID.24916698	0.54	0.47

© 2023 Fernandez-Martinez A et al. *JAMA Oncology.*

Tcells.Bcells.Lymphocyte.Infiltration Calabro BreastCancerResTreat.2009 PMID.18592372	0.54	0.57
Tcells.Effector.Memory Bindea Immunity.2013 PMID.24138885	0.54	0.54
CD103.Negative Broz CancerCell.2014 PMID.25446897	0.54	0.54
Bcells Bindea Immunity.2013 PMID.24138885	0.54	0.57
Tcells.Thelper Bindea Immunity.2013 PMID.24138885	0.54	0.51
TGFB.score TCGA Immunity.2018 PMID.29628290	0.54	0.48
Mast.cells.Activated CIBERSORT NatMethods.2015 PMID.25822800	0.54	0.53
Neutrophils Charoentong CellRep.2017 PMID.28052254	0.54	0.44
NK.Tcell Charoentong CellRep.2017 PMID.28052254	0.54	0.51
Lymphocyte.Infiltration.Expression.Score TCGA Immunity.2018 PMID.29628290	0.54	0.57
Eosinophils Bindea Immunity.2013 PMID.24138885	0.54	0.45
IFN.3.ImmLandscape Wolf PlosOne.2014 PMID.24516633	0.54	0.43
NK Bindea Immunity.2013 PMID.24138885	0.54	0.54
Neutrophils.Activated.Lung Janiszewska NatCellBiol.2019 PMID.31263265	0.54	0.51
NK.CD56bright Charoentong CellRep.2017 PMID.28052254	0.54	0.53
Bcells.Naive CIBERSORT NatMethods.2015 PMID.25822800	0.54	0.54
Cytotoxic.cells Bindea Immunity.2013 PMID.24138885	0.54	0.57
Tcells.Bcell.Cluster Fan BMCMedGenomics.2011 PMID.21214954	0.54	0.59
Macrophages.Th1.Cluster Iglesia CCR.2014 PMID.24916698	0.54	0.58
Tcells.CD8.Effector.Memory Charoentong CellRep.2017 PMID.28052254	0.54	0.60
CSF1.Response TCGA Immunity.2018 PMID.29628290	0.54	0.58
Mast.cells Bindea Immunity.2013 PMID.24138885	0.54	0.63
Eosinophils Charoentong CellRep.2017 PMID.28052254	0.54	0.46
Bcells.Naive Dybaer JCO.2015 PMID.25800755	0.54	0.44
Monocytes.Dendritic.25genes Miller GenomeBiol.2013 PMID.23618380	0.54	0.57
Tcells.Follicular.Helper TCGA Immunity.2018 PMID.29628290	0.54	0.62
IFN Rody BreastCancerResearch.2008 PMID.19272155	0.54	0.58
Tcells.Resident.Memory.Single.cell Savas NatMed.2018 PMID.29942092	0.54	0.61
Tcells.Th17.cells Bindea Immunity.2013 PMID.24138885	0.54	0.44
Bcells.IL10.Plus Lin JImmunol.2014 PMID.25080484	0.53	0.58
MDSC.Neutrophil Youn LeukocBiol.2012 PMID.21954284	0.53	0.59
Tcells.Regulatory.Tregs CIBERSORT NatMethods.2015 PMID.25822800	0.53	0.54
Dendritic.cells Bindea Immunity.2013 PMID.24138885	0.53	0.57
Tcells.NK.Metagene Miller GenomeBiol.2013 PMID.23618380	0.53	0.57
Bcells Garber CellMolGastroenterolHepatol.2017 PMID.28508029	0.53	0.52
Bcells.Memory CIBERSORT NatMethods.2015 PMID.25822800	0.53	0.55
Tcells.Follicular.Helper Bindea Immunity.2013 PMID.24138885	0.53	0.62
Tcells.MCP Petitprez Nature.2020 PMID.31942077	0.53	0.58

© 2023 Fernandez-Martinez A et al. *JAMA Oncology.*

Immune.HLA.D TCGA.BRCA.1198 JCI.2020 PMID.32573490	0.53	0.59
Tcells.CD4.Effector.Memory Charoentong CellRep.2017 PMID.28052254	0.53	0.57
Immune.Cell.Content Verhaak NatCommun.2013 PMID.24113773	0.53	0.57
Tcells.CD8 CIBERSORT NatMethods.2015 PMID.25822800	0.53	0.57
IFN.Pathway ImSig.Nirmal CancerImmunolRes.2018 PMID.30266715	0.53	0.48
Macrophages.ImmuneProfiles.Mouse.Human Shay PNAS.2013 PMID.23382184	0.53	0.58
NK.Activated CIBERSORT NatMethods.2015 PMID.25822800	0.53	0.58
Tcells.Follicular.Helper CIBERSORT NatMethods.2015 PMID.25822800	0.53	0.57
Serum.Response.Up TCGA Immunity.2018 PMID.29628290	0.53	0.48
Dendritic.cells.Resting CIBERSORT NatMethods.2015 PMID.25822800	0.53	0.61
Eosinophils CIBERSORT NatMethods.2015 PMID.25822800	0.53	0.48
Tcells.Follicular.Helper Charoentong CellRep.2017 PMID.28052254	0.53	0.54
Tcells.CD4.Central.Memory Charoentong CellRep.2017 PMID.28052254	0.53	0.56
Neutrophils.MCP Petitprez Nature.2020 PMID.31942077	0.53	0.52
Tcells.Gamma.Delta Bindea Immunity.2013 PMID.24138885	0.53	0.49
Immunosuppression Petitprez Nature.2020 PMID.31942077	0.53	0.54
Tcells.CD4.Memory.Resting CIBERSORT NatMethods.2015 PMID.25822800	0.53	0.55
Bcells.ImmuneProfiles.Mouse.Human Shay PNAS.2013 PMID.23382184	0.53	0.53
Monocytes..Dendritic.cell.Metagene Miller GenomeBiol.2013 PMID.23618380	0.53	0.59
Immune.CD19 TCGA.BRCA.1198 JCI.2020 PMID.32573490	0.53	0.56
Immune.CD34.TIE1 TCGA.BRCA.1198 JCI.2020 PMID.32573490	0.53	0.60
Tcells.CD8.MCP Petitprez Nature.2020 PMID.31942077	0.53	0.54
Tcells.ImmuneProfiles.Mouse.Human Shay PNAS.2013 PMID.23382184	0.53	0.53
HCK Rody BreastCancerResearch.2008 PMID.19272155	0.53	0.57
CTLA4.Pathway GSEA.BIOCARTA ProcNatlAcadSciUSA.2005 PMID.16199517	0.53	0.59
Tcells.Th1.cells Charoentong CellRep.2017 PMID.28052254	0.53	0.58
Tcells.Gamma.Delta TCGA Immunity.2018 PMID.29628290	0.53	0.49
Immune.HLA.A.F TCGA.BRCA.1198 JCI.2020 PMID.32573490	0.53	0.58
Tcells.Regulatory Charoentong CellRep.2017 PMID.28052254	0.53	0.58
Tcells.CD8.Exhausted.Anti.PDL1.vs.Control.Metagene.1 Pauken Science.2016 PMID.27789795	0.53	0.51
Immune.CD4.CD53.CD84.BTK TCGA.BRCA.1198 JCI.2020 PMID.32573490	0.53	0.58
Macrophages.M2 CIBERSORT NatMethods.2015 PMID.25822800	0.53	0.60
Tcells Bindea Immunity.2013 PMID.24138885	0.53	0.56

© 2023 Fernandez-Martinez A et al. *JAMA Oncology.*

Monocytes ImSig.Nirmal CancerImmunolRes.2018 PMID.30266715	0.53	0.40
Macrophages.Monocytes.CSF1.Response Beck CCR.2009 PMID..19188147	0.53	0.58
Macrophages Bindea Immunity.2013 PMID.24138885	0.53	0.57
Dendritic.cells.Immature. Bindea Immunity.2013 PMID.24138885	0.53	0.48
Dendritic.cells.Immature. Charoentong CellRep.2017 PMID.28052254	0.52	0.42
Tcells.CD8 Bindea Immunity.2013 PMID.24138885	0.52	0.61
Cytotoxic.Lymphocytes.MCP Petitprez Nature.2020 PMID.31942077	0.52	0.58
MDSC.Tumor Schlecker JImmunol.2012 PMID.23152559	0.52	0.55
NK.CD56dim Charoentong CellRep.2017 PMID.28052254	0.52	0.60
Tcells.Gamma.Delta Charoentong CellRep.2017 PMID.28052254	0.52	0.51
NK.MCP Helmink Nature.2020 PMID.31942077	0.52	0.48
MHC.24genes Forero CancerImmunolRes.2016 PMID.26980599	0.52	0.52
Bcells.Tcells.Cooperation Hollern Cell.2019 PMID.31730857	0.52	0.55
Immune.14 Perez JCO.2015 PMID.2560586	0.52	0.52
MHC.I Rody BreastCancerResearch.2008 PMID.19272155	0.52	0.62
IFNg.Module11 Gatza ProcNatlAcadSciUSA.2010 PMID.20335537	0.52	0.53
Bcells.IL10.Minus Lin JImmunol.2014 PMID.25080484	0.51	0.46
Neutrophils ImSig.Nirmal CancerImmunolRes.2018 PMID.30266715	0.51	0.59
NK.ImmuneProfiles.Mouse.Human Shay PNAS.2013 PMID.23382184	0.51	0.48
Immune.Hot.vs.Cold.CD8 Cabrita Nature.2020 PMID.31942071	0.51	0.58
Macrophages.M2 Ghassabeh Blood.2006 PMID.16556895	0.51	0.53
Monocytes CIBERSORT NatMethods.2015 PMID.25822800	0.51	0.59
Mast.cells.Resting CIBERSORT NatMethods.2015 PMID.25822800	0.51	0.52
Cytolytic.Activity Rooney Cell.2015 PMID.25594174	0.51	0.55
Plasmacytoid.Dendritic.cell Charoentong CellRep.2017 PMID.28052254	0.51	0.63
IFNa.Module10 Gatza ProcNatlAcadSciUSA.2010 PMID.20335537	0.51	0.51
IFN.Cluster.GSEA.GP11 Fan BMCMedGenomics.2011 PMID.21214954	0.51	0.51
Tcells.Regulatory.cell.2gene Petitprez Nature.2020 PMID.31942077	0.51	0.45
Influenza.11genes Khatri Immunity.2015 PMID.26682989	0.51	0.52
Wound.Healing Chang PlosBiol.2004 PMID.14737219	0.50	0.50
Tcells.Bcell.KEGG.hematopoietic.cell.lineage GSEA.GP2 ProcNatlAcadSciUSA.2005 PMID.16199517	0.50	0.52
Mast.cell Charoentong CellRep.2017 PMID.28052254	0.50	0.50
Granulocytes.ImmuneProfiles.Mouse.Human Shay PNAS.2013 PMID.23382184	0.50	0.56
Macrophages ImSig.Nirmal CancerImmunolRes.2018 PMID.30266715	0.49	0.60

© 2023 Fernandez-Martinez A et al. *JAMA Oncology.*

Using CALGB 40601 as train set, the average AUC of the different models was calculated across all the resamples using 10-fold cross validation. A second AUC was calculated using PAMELA as an external validation set.

© 2023 Fernandez-Martinez A et al. *JAMA Oncology.*

eTable 7. Association of Tumor-Infiltrating Lymphocytes (TILs) and Immune Gene Expression Signatures (iGES) With Event-Free Survival (EFS) in CALGB 40601

Signature	HR	Lower CI	Upper CI	P-value	AIC
Bcells.Plasma.cells.Metagene Miller GenomeBiol.2013 PMID.23618380	0.55	0.42	0.73	0.007	401.97
Bcells.Plasma.cells.52genes Miller GenomeBiol.2013 PMID.23618380	0.56	0.42	0.74	0.007	403.55
Ig TCGA.BRCA.1198 Cell.2015 PMID.26451490	0.56	0.42	0.75	0.007	403.91
IgG Rody BreastCancerResearch.2008 PMID.19272155	0.60	0.45	0.80	0.02	406.92
IGG.Cluster Fan BMCMedGenomics.2011 PMID.21214954	0.59	0.44	0.80	0.02	406.97
Immune.14 Perez JCO.2015 PMID.2560586	0.60	0.45	0.81	0.02	407.17
Immune1 TCGA.BRCA.1198 JCI.2020 PMID.32573490	0.59	0.44	0.80	0.02	407.03
Cytotoxic.Lymphocytes.MCP Petitprez Nature.2020 PMID.31942077	0.61	0.45	0.83	0.04	408.82
Plasma.cells ImSig.Nirmal CancerImmunolRes.2018 PMID.30266715	0.64	0.49	0.85	0.04	409.32
MHC.24genes Forero CancerImmunolRes.2016 PMID.26980599	0.59	0.42	0.82	0.04	408.55
Tcells.Follicular.Helper CIBERSORT NatMethods.2015 PMID.25822800	0.61	0.44	0.83	0.04	408.97
Bcells.Tcells.Cooperation Hollern Cell.2019 PMID.31730857	0.62	0.46	0.84	0.04	408.91
TLS.High.In.Response.MCP Helmink Nature.2020 PMID.31942075	0.63	0.47	0.84	0.04	409.27
Immune.87 Perez JCO.2015 PMID.2560586	0.62	0.46	0.85	0.04	409.41
Tcells.CD8.MCP Petitprez Nature.2020 PMID.31942077	0.60	0.43	0.84	0.04	409.44
Tcells.CD4.Naive CIBERSORT NatMethods.2015 PMID.25822800	0.64	0.47	0.86	0.04	409.78
Bcells.Cluster Iglesia CCR.2014 PMID.24916698	0.62	0.45	0.85	0.04	409.39
Tcells.Cluster Iglesia CCR.2014 PMID.24916698	0.63	0.46	0.86	0.04	410.00
Tcells.CD8 CIBERSORT NatMethods.2015 PMID.25822800	0.63	0.46	0.86	0.04	410.11
Tcells.Gamma.Delta CIBERSORT NatMethods.2015 PMID.25822800	0.64	0.48	0.87	0.04	410.39
NK ImSig.Nirmal CancerImmunolRes.2018 PMID.30266715	0.63	0.46	0.87	0.04	410.40
PD1.Signaling.Reactome GSEA ProcNatlAcadSciUSA.2005 PMID.16199517	0.63	0.46	0.87	0.04	410.40
Tcells.Bcells.Lymphocyte.Infiltration Calabro BreastCancerResTreat.2009 PMID.18592372	0.63	0.46	0.87	0.04	410.33
Lymphocyte.Infiltration.Expression.Score TCGA Immunity.2018 PMID.29628290	0.63	0.46	0.87	0.04	410.33

© 2023 Fernandez-Martinez A et al. *JAMA Oncology.*

TLS.Tumors.w.TLS.and.CD8.vs.CD8.alone Cabrita Nature.2020 PMID.31942071	0.63	0.45	0.87	0.04	410.21
Tcells.CD8.Activated Charoentong CellRep.2017 PMID.28052254	0.65	0.47	0.88	0.04	410.91
TLS.Hallmark Cabrita Nature.2020 PMID.31942071	0.63	0.46	0.87	0.04	410.83
Immune.CD8.GZMK TCGA.BRCA.1198 JCI.2020 PMID.32573490	0.64	0.47	0.88	0.04	410.73
CD8.Cluster Iglesia CCR.2014 PMID.24916698	0.65	0.47	0.88	0.04	410.82
Tcells.CD4.Memory.Resting CIBERSORT NatMethods.2015 PMID.25822800	0.64	0.47	0.88	0.04	410.81
Tcells.Regulatory.Tregs CIBERSORT NatMethods.2015 PMID.25822800	0.65	0.48	0.89	0.04	410.98
NK.CD56dim Charoentong CellRep.2017 PMID.28052254	1.51	1.12	2.03	0.04	410.92
CTLA4.Pathway GSEA.BIOCARTA ProcNatlAcadSciUSA.2005 PMID.16199517	0.65	0.48	0.89	0.04	411.16
Immune.CD19 TCGA.BRCA.1198 JCI.2020 PMID.32573490	0.63	0.45	0.88	0.04	410.74
Bcells.Activated Charoentong CellRep.2017 PMID.28052254	0.65	0.47	0.89	0.04	411.10
NK.Resting CIBERSORT NatMethods.2015 PMID.25822800	0.64	0.46	0.89	0.04	411.14
Bcells.Memory CIBERSORT NatMethods.2015 PMID.25822800	0.64	0.47	0.89	0.04	411.21

Cox regression models for EFS prediction in CALGB 40601 have been adjusted by treatment arm. Only the models significantly associated with EFS with a p-value <0.05 are shown. P-values are adjusted for multiple testing using a Benjamini & Hochberg method to control the False Discovery Rate.

© 2023 Fernandez-Martinez A et al. *JAMA Oncology.*

eTable 8. Association of Tumor-Infiltrating Lymphocytes (TILs) as a Continuous Variable and Immune Gene Expression Signatures (iGES) With Event-Free Survival (EFS) in CALGB 40601

Comparative analysis of nested multivariable Cox regression models.

**Signature:Plasma.cells ImSig.Nirmal CancerImmunolRes.2018 PMID.30266715**						
**Model**	**Features**	**HR**	**95% CI**	**p-value[1]**	**AIC**	**LR p-value[2]**
**Model 1**	THL vs. TH	0.34	0.14, 0.78	**0.01**	395.68	-
	TL vs. TH	1.27	0.62, 2.60	0.51		
	HR (pos vs. neg)	1.86	0.94, 3.69	0.08		
	Stage (III vs. II)	2.03	1.07, 3.87	**0.03**		
	pCR (pCR vs. RD)	0.22	0.10, 0.48	**<0.001**		
	HER2-E vs. Other	4.2	1.97, 8.96	**<0.001**		
**Model 2**	THL vs. TH	0.31	0.13, 0.73	**0.007**	395.31	Model 2 vs. Model 1 LRT p-value 0.12
	TL vs. TH	1.3	0.63, 2.67	0.48		
	HR (pos vs. neg)	1.85	0.93, 3.69	0.08		
	Stage (III vs. II)	1.95	1.02, 3.71	**0.04**		
	pCR (pCR vs. RD)	0.24	0.11, 0.54	**<0.001**		
	HER2-E vs. not	4.53	2.11, 9.73	**<0.001**		
	TILs (continuous)	0.99	0.97, 1.00	0.14		
**Model 3**	THL vs. TH	0.3	0.13, 0.71	**0.006**	388.34	Model 3 vs. Model 1 LRT p-value **<0.001**
	TL vs. TH	1.55	0.74, 3.26	0.24		
	HR (pos vs. neg)	1.84	0.93, 3.63	0.08		
	Stage (III vs. II)	2.04	1.07, 3.89	**0.03**		
	pCR (pCR vs. RD)	0.32	0.14, 0.74	**0.007**		
	HER2-E vs. not	5.43	2.47, 11.9	**<0.001**		
	Signature (cont)	0.59	0.42, 0.82	**0.002**		
**Model 4**	THL vs. TH	0.3	0.13, 0.71	**0.006**	390.32	Model 4 vs. Model 2 LRT p-value **0.008**
	TL vs. TH	1.56	0.74, 3.29	0.24		
	HR (pos vs. neg)	1.84	0.93, 3.62	0.08		
	Stage (III vs. II)	2.05	1.07, 3.91	**0.03**		
	pCR (pCR vs. RD)	0.32	0.14, 0.74	**0.007**		Model 4 vs. Model 3 LRT p-value 0.90
	HER2-E vs. not	5.43	2.47, 12.0	**<0.001**		
	TILs (continuous)	1	0.98, 1.02	0.90		
	Signature (continuous)	0.58	0.39, 0.86	**0.007**		
**Signature: Bcells.Plasma.cells.52genes Miller GenomeBiol.2013 PMID.23618380**						
**Model**	**Features**	**HR**	**95% CI**	**p-value[1]**	**AIC**	**LR p-value[2]**
**Model 1**	THL vs. TH	0.34	0.14, 0.78	**0.01**	395.68	-
	TL vs. TH	1.27	0.62, 2.60	0.51		
	HR (pos vs. neg)	1.86	0.94, 3.69	0.08		
	Stage (III vs. II)	2.03	1.07, 3.87	**0.03**		
	pCR (pCR vs. RD)	0.22	0.10, 0.48	**<0.001**		

© 2023 Fernandez-Martinez A et al. *JAMA Oncology.*

	HER2-E vs. Other	4.2	1.97, 8.96	**<0.001**		
**Model 2**	THL vs. TH	0.31	0.13, 0.73	**0.007**	395.31	Model 2 vs. Model 1 LRT p-value 0.12
	TL vs. TH	1.3	0.63, 2.67	0.48		
	HR (pos vs. neg)	1.85	0.93, 3.69	0.08		
	Stage (III vs. II)	1.95	1.02, 3.71	**0.04**		
	pCR (pCR vs. RD)	0.24	0.11, 0.54	**<0.001**		
	HER2-E vs. not	4.53	2.11, 9.73	**<0.001**		
	TILs (continuous)	0.99	0.97, 1.00	0.14		
**Model 3**	THL vs. TH	0.29	0.12, 0.68	**0.005**	385.04	Model 3 vs. Model 1 LRT p-value **0.004**
	TL vs. TH	1.57	0.75, 3.27	0.23		
	HR (pos vs. neg)	1.36	0.68, 2.73	0.39		
	Stage (III vs. II)	2.2	1.15, 4.19	**0.02**		
	pCR (pCR vs. RD)	0.34	0.15, 0.76	**0.008**		
	HER2-E vs. not	4.73	2.20, 10.1	**<0.001**		
	Signature (cont)	0.53	0.38, 0.75	**<0.001**		
**Model 4**	THL vs. TH	0.3	0.13, 0.71	**0.006**	386.88	Model 4 vs. Model 2 LRT p-value **0.001**
	TL vs. TH	1.6	0.76, 3.36	0.22		
	HR (pos vs. neg)	1.34	0.66, 2.69	0.42		
	Stage (III vs. II)	2.23	1.17, 4.26	**0.02**		
	pCR (pCR vs. RD)	0.33	0.15, 0.75	**0.008**		Model 4 vs. Model 3 LRT p-value 0.69
	HER2-E vs. not	4.68	2.17, 10.1	**<0.001**		
	TILs (continuous)	1	0.99, 1.02	0.68		
	Signature (continuous)	0.51	0.34, 0.76	**<0.001**		
**Signature: Ig TCGA.BRCA.1198 Cell.2015 PMID.26451490**						
**Model**	**Features**	**HR**	**95% CI**	**p-value[1]**	**AIC**	**LR p-value[2]**
**Model 1**	THL vs. TH	0.34	0.14, 0.78	**0.01**	395.68	-
	TL vs. TH	1.27	0.62, 2.60	0.51		
	HR (pos vs. neg)	1.86	0.94, 3.69	0.08		
	Stage (III vs. II)	2.03	1.07, 3.87	**0.03**		
	pCR (pCR vs. RD)	0.22	0.10, 0.48	**<0.001**		
	HER2-E vs. Other	4.2	1.97, 8.96	**<0.001**		
**Model 2**	THL vs. TH	0.31	0.13, 0.73	**0.007**	395.31	Model 2 vs. Model 1 LRT p-value 0.12
	TL vs. TH	1.3	0.63, 2.67	0.48		
	HR (pos vs. neg)	1.85	0.93, 3.69	0.08		
	Stage (III vs. II)	1.95	1.02, 3.71	**0.04**		
	pCR (pCR vs. RD)	0.24	0.11, 0.54	**<0.001**		
	HER2-E vs. not	4.53	2.11, 9.73	**<0.001**		
	TILs (continuous)	0.99	0.97, 1.00	0.14		
**Model 3**	THL vs. TH	0.31	0.13, 0.73	**0.007**	384.82	Model 3 vs. Model 1 LRT p-value **<0.001**
	TL vs. TH	1.71	0.81, 3.59	0.16		
	HR (pos vs. neg)	1.48	0.74, 2.93	0.27		
	Stage (III vs. II)	2.34	1.22, 4.47	**0.01**		

© 2023 Fernandez-Martinez A et al. *JAMA Oncology.*

	pCR (pCR vs. RD)	0.34	0.15, 0.77	**0.01**		
	HER2-E vs. not	4.81	2.24, 10.3	**<0.001**		
	Signature (cont)	0.53	0.37, 0.75	**<0.001**		
**Model 4**	THL vs. TH	0.32	0.14, 0.75	**0.009**	386.66	Model 4 vs. Model 2 LRT p-value **0.001**
	TL vs. TH	1.75	0.82, 3.72	0.15		
	HR (pos vs. neg)	1.47	0.74, 2.90	0.27		
	Stage (III vs. II)	2.38	1.24, 4.59	**0.01**		
	pCR (pCR vs. RD)	0.34	0.15, 0.76	**0.009**		Model 4 vs. Model 3 LRT p-value 0.69
	HER2-E vs. not	4.77	2.22, 10.2	**<0.001**		
	TILs (continuous)	1	0.99, 1.02	0.69		
	Signature (continuous)	0.5	0.33, 0.76	**0.001**		
**Signature: Immune1 TCGA.BRCA.1198 JCI.2020 PMID.32573490**						
**Model**	**Features**	**HR**	**95% CI**	**p-value[1]**	**AIC**	**LR p-value[2]**
**Model 1**	THL vs. TH	0.34	0.14, 0.78	**0.01**	395.68	-
	TL vs. TH	1.27	0.62, 2.60	0.51		
	HR (pos vs. neg)	1.86	0.94, 3.69	0.08		
	Stage (III vs. II)	2.03	1.07, 3.87	**0.03**		
	pCR (pCR vs. RD)	0.22	0.10, 0.48	**<0.001**		
	HER2-E vs. Other	4.2	1.97, 8.96	**<0.001**		
**Model 2**	THL vs. TH	0.31	0.13, 0.73	**0.007**	395.31	Model 2 vs. Model 1 LRT p-value 0.12
	TL vs. TH	1.3	0.63, 2.67	0.48		
	HR (pos vs. neg)	1.85	0.93, 3.69	0.08		
	Stage (III vs. II)	1.95	1.02, 3.71	**0.04**		
	pCR (pCR vs. RD)	0.24	0.11, 0.54	**<0.001**		
	HER2-E vs. not	4.53	2.11, 9.73	**<0.001**		
	TILs (continuous)	0.99	0.97, 1.00	0.14		
**Model 3**	THL vs. TH	0.3	0.13, 0.71	**0.006**	389.43	Model 3 vs. Model 1 LRT p-value **0.004**
	TL vs. TH	1.49	0.72, 3.11	0.29		
	HR (pos vs. neg)	1.47	0.73, 2.95	0.28		
	Stage (III vs. II)	2.1	1.10, 4.00	**0.03**		
	pCR (pCR vs. RD)	0.31	0.14, 0.71	**0.005**		
	HER2-E vs. not	4.41	2.07, 9.38	**<0.001**		
	Signature (cont)	0.6	0.43, 0.85	**0.004**		
**Model 4**	THL vs. TH	0.3	0.13, 0.71	**0.006**	391.41	Model 4 vs. Model 2 LRT p-value **0.02**
	TL vs. TH	1.5	0.72, 3.14	0.28		
	HR (pos vs. neg)	1.46	0.72, 2.95	0.30		
	Stage (III vs. II)	2.11	1.10, 4.03	**0.02**		
	pCR (pCR vs. RD)	0.31	0.14, 0.71	**0.005**		Model 4 vs. Model 3 LRT p-value 0.88
	HER2-E vs. not	4.39	2.06, 9.36	**<0.001**		
	TILs (continuous)	1	0.98, 1.02	0.88		
	Signature (continuous)	0.59	0.39, 0.90	**0.014**		

© 2023 Fernandez-Martinez A et al. *JAMA Oncology.*

Signature: IgG Rody BreastCancerResearch.2008 PMID.19272155						
**Model**	**Features**	**HR**	**95% CI**	**p-value[1]**	**AIC**	**LR p-value[2]**
**Model 1**	THL vs. TH	0.34	0.14, 0.78	**0.011**	395.68	-
	TL vs. TH	1.27	0.62, 2.60	0.51		
	HR (pos vs. neg)	1.86	0.94, 3.69	0.08		
	Stage (III vs. II)	2.03	1.07, 3.87	**0.03**		
	pCR (pCR vs. RD)	0.22	0.10, 0.48	**<0.001**		
	HER2-E vs. Other	4.2	1.97, 8.96	**<0.001**		
**Model 2**	THL vs. TH	0.31	0.13, 0.73	**0.007**	395.31	Model 2 vs. Model 1 LRT p-value 0.12
	TL vs. TH	1.3	0.63, 2.67	0.48		
	HR (pos vs. neg)	1.85	0.93, 3.69	0.08		
	Stage (III vs. II)	1.95	1.02, 3.71	**0.04**		
	pCR (pCR vs. RD)	0.24	0.11, 0.54	**<0.001**		
	HER2-E vs. not	4.53	2.11, 9.73	**<0.001**		
	TILs (continuous)	0.99	0.97, 1.00	0.14		
**Model 3**	THL vs. TH	0.33	0.14, 0.76	**0.009**	387.85	Model 3 vs. Model 1 LRT p-value **0.002**
	TL vs. TH	1.59	0.76, 3.32	0.22		
	HR (pos vs. neg)	1.52	0.76, 3.02	0.24		
	Stage (III vs. II)	2.3	1.20, 4.40	**0.01**		
	pCR (pCR vs. RD)	0.31	0.14, 0.69	**0.004**		
	HER2-E vs. not	4.71	2.20, 10.1	**<0.001**		
	Signature (cont)	0.57	0.41, 0.81	**0.002**		
**Model 4**	THL vs. TH	0.33	0.14, 0.77	**0.01**	389.85	Model 4 vs. Model 2 LRT p-value **0.006**
	TL vs. TH	1.59	0.76, 3.35	0.22		
	HR (pos vs. neg)	1.51	0.76, 3.02	0.24		
	Stage (III vs. II)	2.31	1.20, 4.45	**0.01**		
	pCR (pCR vs. RD)	0.31	0.14, 0.69	**0.004**		Model 4 vs. Model 3 LRT p-value 0.96
	HER2-E vs. not	4.7	2.20, 10.1	**<0.001**		
	TILs (continuous)	1	0.98, 1.02	0.96		
	Signature (continuous)	0.57	0.38, 0.85	**0.006**		

CI: confident intervals; EFS: event-free survival; HR: hormone receptor; pCR: pathologic complete response; RD: residual disease; pos: positive; neg: negative; HER2-E: HER2-Enriched; AIC: Akaike Information Criterion; LR: likelihood-ratio test; TILs: tumor infiltrating lymphocytes; IgG: immunoglobulin G; T: weekly paclitaxel; H: trastuzumab; L: lapatinib. [1]Cox regression model p-value; [2]Likelihood-ratio test p-value.

© 2023 Fernandez-Martinez A et al. *JAMA Oncology.*

eTable 9. Association of Tumor-Infiltrating Lymphocytes (TILs) Using a Cutoff of 40% and Immune Gene Expression Signatures (iGES) With Event-Free Survival (EFS) in CALGB 40601

Comparative analysis of nested multivariable Cox regression models.

Signature: IGG.Cluster Fan BMCMedGenomics.2011 PMID.21214954						
**Model**	**Features**	**HR**	**95% CI**	**p-value[1]**	**AIC**	**LR p-value[2]**
**Model 1**	THL vs. TH	0.34	0.14, 0.78	**0.011**	395.68	-
	TL vs. TH	1.27	0.62, 2.60	0.511		
	HR (pos vs. neg)	1.86	0.94, 3.69	0.076		
	Stage (III vs. II)	2.03	1.07, 3.87	**0.031**		
	pCR (pCR vs. RD)	0.22	0.10, 0.48	**<0.001**		
	HER2-E vs. Other	4.2	1.97, 8.96	**<0.001**		
**Model 2**	THL vs. TH	0.31	0.13, 0.73	**0.007**	394.78	Model 2 vs. Model 1 LRT p-value 0.09
	TL vs. TH	1.27	0.62, 2.61	0.513		
	HR (pos vs. neg)	1.87	0.94, 3.73	0.076		
	Stage (III vs. II)	1.92	1.01, 3.66	**0.046**		
	pCR (pCR vs. RD)	0.25	0.11, 0.54	**<0.001**		
	HER2-E vs. not	4.51	2.10, 9.68	**<0.001**		
	TILs High vs. Low (40%)	0.49	0.20, 1.19	0.113		
**Model 3**	THL vs. TH	0.31	0.13, 0.71	**0.006**	389.97	Model 3 vs. Model 1 LRT p-value **0.005**
	TL vs. TH	1.45	0.70, 3.02	0.322		
	HR (pos vs. neg)	1.43	0.71, 2.90	0.319		
	Stage (III vs. II)	2.01	1.06, 3.83	**0.033**		
	pCR (pCR vs. RD)	0.3	0.13, 0.66	**0.003**		
	HER2-E vs. not	4.28	2.02, 9.08	**<0.001**		
	Signature (cont)	0.63	0.45, 0.87	**0.006**		
**Model 4**	THL vs. TH	0.3	0.13, 0.70	**0.006**	391.74	Model 4 vs. Model 2 LRT p-value **0.03**
	TL vs. TH	1.43	0.69, 2.99	0.339		
	HR (pos vs. neg)	1.46	0.72, 2.97	0.299		
	Stage (III vs. II)	2	1.05, 3.80	**0.035**		
	pCR (pCR vs. RD)	0.3	0.13, 0.68	**0.004**		Model 4 vs. Model 3

© 2023 Fernandez-Martinez A et al. *JAMA Oncology.*

	HER2-E vs. not	4.33	2.04, 9.21	**<0.001**		LRT p-value 0.63
	TILs High vs. Low (40%)	0.79	0.29, 2.14	0.639		
	Signature (continuous)	0.65	0.45, 0.95	**0.024**		
**Signature: Plasma.cells ImSig.Nirmal CancerImmunolRes.2018 PMID.30266715**						
**Model**	**Features**	**HR**	**95% CI**	**p-value[1]**	**AIC**	**LR p-value[2]**
**Model 1**	THL vs. TH	0.34	0.14, 0.78	**0.011**	395.68	-
	TL vs. TH	1.27	0.62, 2.60	0.511		
	HR (pos vs. neg)	1.86	0.94, 3.69	0.076		
	Stage (III vs. II)	2.03	1.07, 3.87	**0.031**		
	pCR (pCR vs. RD)	0.22	0.10, 0.48	**<0.001**		
	HER2-E vs. Other	4.2	1.97, 8.96	**<0.001**		
**Model 2**	THL vs. TH	0.31	0.13, 0.73	**0.007**	394.78	Model 2 vs. Model 1 LRT p-value 0.09
	TL vs. TH	1.27	0.62, 2.61	0.513		
	HR (pos vs. neg)	1.87	0.94, 3.73	0.076		
	Stage (III vs. II)	1.92	1.01, 3.66	**0.046**		
	pCR (pCR vs. RD)	0.25	0.11, 0.54	**<0.001**		
	HER2-E vs. not	4.51	2.10, 9.68	**<0.001**		
	TILs High vs. Low (40%)	0.49	0.20, 1.19	0.113		
**Model 3**	THL vs. TH	0.3	0.13, 0.71	**0.006**	388.34	Model 3 vs. Model 1 LRT p-value **0.002**
	TL vs. TH	1.55	0.74, 3.26	0.243		
	HR (pos vs. neg)	1.84	0.93, 3.63	0.081		
	Stage (III vs. II)	2.04	1.07, 3.89	**0.031**		
	pCR (pCR vs. RD)	0.32	0.14, 0.74	**0.007**		
	HER2-E vs. not	5.43	2.47, 11.9	**<0.001**		
	Signature (cont)	0.59	0.42, 0.82	**0.002**		
**Model 4**	THL vs. TH	0.3	0.13, 0.70	**0.005**	390.20	Model 4 vs. Model 2 LRT p-value **0.01**
	TL vs. TH	1.54	0.73, 3.23	0.257		
	HR (pos vs. neg)	1.84	0.93, 3.64	0.08		
	Stage (III vs. II)	2.03	1.06, 3.87	**0.032**		

© 2023 Fernandez-Martinez A et al. *JAMA Oncology.*

	pCR (pCR vs. RD)	0.33	0.14, 0.75	**0.008**		Model 4 vs. Model 3 LRT p-value 0.71
	HER2-E vs. not	5.4	2.46, 11.9	**<0.001**		
	TILs High vs. Low (40%)	0.83	0.30, 2.26	0.714		
	Signature (continuous)	0.61	0.42, 0.88	**0.009**		
**Signature: Bcells.Plasma.cells.52genes Miller GenomeBiol.2013 PMID.23618380**						
**Model**	**Features**	**HR**	**95% CI**	**p-value[1]**	**AIC**	**LR p-value[2]**
**Model 1**	THL vs. TH	0.34	0.14, 0.78	**0.011**	395.68	-
	TL vs. TH	1.27	0.62, 2.60	0.511		
	HR (pos vs. neg)	1.86	0.94, 3.69	0.076		
	Stage (III vs. II)	2.03	1.07, 3.87	**0.031**		
	pCR (pCR vs. RD)	0.22	0.10, 0.48	**<0.001**		
	HER2-E vs. Other	4.2	1.97, 8.96	**<0.001**		
**Model 2**	THL vs. TH	0.31	0.13, 0.73	**0.007**	394.78	Model 2 vs. Model 1 LRT p-value 0.09
	TL vs. TH	1.27	0.62, 2.61	0.513		
	HR (pos vs. neg)	1.87	0.94, 3.73	0.076		
	Stage (III vs. II)	1.92	1.01, 3.66	**0.046**		
	pCR (pCR vs. RD)	0.25	0.11, 0.54	**<0.001**		
	HER2-E vs. not	4.51	2.10, 9.68	**<0.001**		
	TILs High vs. Low (40%)	0.49	0.20, 1.19	0.113		
**Model 3**	THL vs. TH	0.29	0.12, 0.68	**0.005**	385.04	Model 3 vs. Model 1 LRT p-value **<0.001**
	TL vs. TH	1.57	0.75, 3.27	0.234		
	HR (pos vs. neg)	1.36	0.68, 2.73	0.388		
	Stage (III vs. II)	2.2	1.15, 4.19	**0.016**		
	pCR (pCR vs. RD)	0.34	0.15, 0.76	**0.008**		
	HER2-E vs. not	4.73	2.20, 10.1	**<0.001**		
	Signature (cont)	0.53	0.38, 0.75	**<0.001**		
**Model 4**	THL vs. TH	0.29	0.12, 0.68	**0.005**	387.03	Model 4 vs. Model 2 LRT p-value **0.002**
	TL vs. TH	1.56	0.74, 3.27	0.243		
	HR (pos vs. neg)	1.36	0.68, 2.75	0.385		

© 2023 Fernandez-Martinez A et al. *JAMA Oncology.*

Model	Features	HR	95% CI	p-value[1]	AIC	LR p-value[2]
	Stage (III vs. II)	2.19	1.15, 4.19	**0.017**		
	pCR (pCR vs. RD)	0.34	0.15, 0.76	**0.009**		Model 4 vs. Model 3 LRT p-value 0.92
	HER2-E vs. not	4.73	2.20, 10.2	**<0.001**		
	TILs High vs. Low (40%)	0.95	0.35, 2.59	0.915		
	Signature (continuous)	0.54	0.37, 0.78	**0.001**		
**Signature: Ig TCGA.BRCA.1198 Cell.2015 PMID.26451490**						
**Model**	**Features**	**HR**	**95% CI**	**p-value[1]**	**AIC**	**LR p-value[2]**
**Model 1**	THL vs. TH	0.34	0.14, 0.78	**0.011**	395.68	-
	TL vs. TH	1.27	0.62, 2.60	0.511		
	HR (pos vs. neg)	1.86	0.94, 3.69	0.076		
	Stage (III vs. II)	2.03	1.07, 3.87	**0.031**		
	pCR (pCR vs. RD)	0.22	0.10, 0.48	**<0.001**		
	HER2-E vs. Other	4.2	1.97, 8.96	**<0.001**		
**Model 2**	THL vs. TH	0.31	0.13, 0.73	**0.007**	394.78	Model 2 vs. Model 1 LRT p-value 0.09
	TL vs. TH	1.27	0.62, 2.61	0.513		
	HR (pos vs. neg)	1.87	0.94, 3.73	0.076		
	Stage (III vs. II)	1.92	1.01, 3.66	**0.046**		
	pCR (pCR vs. RD)	0.25	0.11, 0.54	**<0.001**		
	HER2-E vs. not	4.51	2.10, 9.68	**<0.001**		
	TILs High vs. Low (40%)	0.49	0.20, 1.19	0.113		
**Model 3**	THL vs. TH	0.31	0.13, 0.73	**0.007**	384.82	Model 3 vs. Model 1 LRT p-value **<0.001**
	TL vs. TH	1.71	0.81, 3.59	0.16		
	HR (pos vs. neg)	1.48	0.74, 2.93	0.265		
	Stage (III vs. II)	2.34	1.22, 4.47	**0.01**		
	pCR (pCR vs. RD)	0.34	0.15, 0.77	**0.01**		
	HER2-E vs. not	4.81	2.24, 10.3	**<0.001**		
	Signature (cont)	0.53	0.37, 0.75	**<0.001**		
**Model 4**	THL vs. TH	0.31	0.13, 0.73	**0.007**	386.82	Model 4 vs. Model 2
	TL vs. TH	1.7	0.80, 3.61	0.169		

© 2023 Fernandez-Martinez A et al. *JAMA Oncology.*

	HR (pos vs. neg)	1.48	0.74, 2.94	0.264		LRT p-value **0.002**
	Stage (III vs. II)	2.33	1.21, 4.48	**0.011**		
	pCR (pCR vs. RD)	0.34	0.15, 0.78	**0.01**		Model 4 vs. Model 3 LRT p-value 0.94
	HER2-E vs. not	4.81	2.25, 10.3	**<0.001**		
	TILs High vs. Low (40%)	0.96	0.35, 2.63	0.942		
	Signature (continuous)	0.53	0.36, 0.78	**0.001**		
**Signature: Immune1 TCGA.BRCA.1198 JCI.2020 PMID.32573490**						
**Model**	**Features**	**HR**	**95% CI**	**p-value[1]**	**AIC**	**LR p-value[2]**
**Model 1**	THL vs. TH	0.34	0.14, 0.78	**0.011**	395.68	-
	TL vs. TH	1.27	0.62, 2.60	0.511		
	HR (pos vs. neg)	1.86	0.94, 3.69	0.076		
	Stage (III vs. II)	2.03	1.07, 3.87	**0.031**		
	pCR (pCR vs. RD)	0.22	0.10, 0.48	**<0.001**		
	HER2-E vs. Other	4.2	1.97, 8.96	**<0.001**		
**Model 2**	THL vs. TH	0.31	0.13, 0.73	**0.007**	394.78	Model 2 vs. Model 1 LRT p-value 0.09
	TL vs. TH	1.27	0.62, 2.61	0.513		
	HR (pos vs. neg)	1.87	0.94, 3.73	0.076		
	Stage (III vs. II)	1.92	1.01, 3.66	**0.046**		
	pCR (pCR vs. RD)	0.25	0.11, 0.54	**<0.001**		
	HER2-E vs. not	4.51	2.10, 9.68	**<0.001**		
	TILs High vs. Low (40%)	0.49	0.20, 1.19	0.113		
**Model 3**	THL vs. TH	0.3	0.13, 0.71	**0.006**	389.43	Model 3 vs. Model 1 LRT p-value **0.004**
	TL vs. TH	1.49	0.72, 3.11	0.285		
	HR (pos vs. neg)	1.47	0.73, 2.95	0.282		
	Stage (III vs. II)	2.1	1.10, 4.00	**0.025**		
	pCR (pCR vs. RD)	0.31	0.14, 0.71	**0.005**		
	HER2-E vs. not	4.41	2.07, 9.38	**<0.001**		
	Signature (cont)	0.6	0.43, 0.85	**0.004**		
**Model 4**	THL vs. TH	0.3	0.13, 0.70	**0.005**	391.31	

© 2023 Fernandez-Martinez A et al. *JAMA Oncology.*

	TL vs. TH	1.48	0.71, 3.09	0.30		Model 4 vs. Model 2 LRT p-value **0.02**
	HR (pos vs. neg)	1.49	0.73, 3.00	0.271		
	Stage (III vs. II)	2.08	1.09, 3.97	**0.027**		
	pCR (pCR vs. RD)	0.32	0.14, 0.71	**0.006**		Model 4 vs. Model 3 LRT p-value 0.73
	HER2-E vs. not	4.44	2.09, 9.46	**<0.001**		
	TILs High vs. Low (40%)	0.84	0.30, 2.32	0.733		
	Signature (continuous)	0.62	0.42, 0.92	**0.019**		
**Signature: IgG Rody BreastCancerResearch.2008 PMID.19272155**						
**Model**	**Features**	**HR**	**95% CI**	**p-value[1]**	**AIC**	**LR p-value[2]**
**Model 1**	THL vs. TH	0.34	0.14, 0.78	**0.011**	395.68	-
	TL vs. TH	1.27	0.62, 2.60	0.511		
	HR (pos vs. neg)	1.86	0.94, 3.69	0.076		
	Stage (III vs. II)	2.03	1.07, 3.87	**0.031**		
	pCR (pCR vs. RD)	0.22	0.10, 0.48	**<0.001**		
	HER2-E vs. Other	4.2	1.97, 8.96	**<0.001**		
**Model 2**	THL vs. TH	0.31	0.13, 0.73	**0.007**	394.78	Model 2 vs. Model 1 LRT p-value 0.09
	TL vs. TH	1.27	0.62, 2.61	0.513		
	HR (pos vs. neg)	1.87	0.94, 3.73	0.076		
	Stage (III vs. II)	1.92	1.01, 3.66	**0.046**		
	pCR (pCR vs. RD)	0.25	0.11, 0.54	**<0.001**		
	HER2-E vs. not	4.51	2.10, 9.68	**<0.001**		
	TILs High vs. Low (40%)	0.49	0.20, 1.19	0.113		
**Model 3**	THL vs. TH	0.33	0.14, 0.76	**0.009**	387.85	Model 3 vs. Model 1 LRT p-value **0.002**
	TL vs. TH	1.59	0.76, 3.32	0.217		
	HR (pos vs. neg)	1.52	0.76, 3.02	0.236		
	Stage (III vs. II)	2.3	1.20, 4.40	**0.012**		
	pCR (pCR vs. RD)	0.31	0.14, 0.69	**0.004**		
	HER2-E vs. not	4.71	2.20, 10.1	**<0.001**		
	Signature (cont)	0.57	0.41, 0.81	**0.002**		

© 2023 Fernandez-Martinez A et al. *JAMA Oncology.*

**Model 4**	THL vs. TH	0.32	0.14, 0.75	**0.009**	389.76	Model 4 vs. Model 2 LRT p-value **0.008**
	TL vs. TH	1.57	0.75, 3.30	0.234		
	HR (pos vs. neg)	1.53	0.76, 3.06	0.229		
	Stage (III vs. II)	2.27	1.18, 4.37	**0.014**		
	pCR (pCR vs. RD)	0.31	0.14, 0.69	**0.004**		Model 4 vs. Model 3 LRT p-value 0.77
	HER2-E vs. not	4.72	2.21, 10.1	**<0.001**		
	TILs High vs. Low (40%)	0.86	0.32, 2.34	0.77		
	Signature (continuous)	0.59	0.40, 0.87	**0.007**		

CI: confident intervals; EFS: event-free survival; HR: hormone receptor; pCR: pathologic complete response; RD: residual disease; pos: positive; neg: negative; HER2-E: HER2-Enriched; AIC: Akaike Information Criterion; LR: likelihood-ratio test; TILs: tumor infiltrating lymphocytes; IgG: immunoglobulin G; T: weekly paclitaxel; H: trastuzumab; L: lapatinib. [1]Cox regression model p-value; [2]Likelihood-ratio test p-value.

© 2023 Fernandez-Martinez A et al. *JAMA Oncology.*

eTable 10. Landmark Analysis Week 30

Association of tumor-infiltrating lymphocytes (TILs) as continuous variable and immune gene expression signatures (iGES) with event-free survival (EFS) in CALGB 40601: comparative analysis of nested multivariable Cox regression models.

Signature: IGG.Cluster Fan BMCMedGenomics.2011 PMID.21214954						
**Model**	**Features**	**HR**	**95% CI**	**p-value[1]**	**AIC**	**LR p-value[2]**
**Model 1**	THL vs. TH	0.37	0.16, 0.87	**0.02**	364.58	-
	TL vs. TH	1.34	0.63, 2.83	0.44		
	HR (pos vs. neg)	2.13	1.03, 4.42	**0.04**		
	Stage (III vs. II)	1.95	0.99, 3.83	0.05		
	pCR (pCR vs. RD)	0.2	0.09, 0.46	**<0.001**		
	HER2-E vs. Other	4.6	2.09, 10.1	**<0.001**		
**Model 2**	THL vs. TH	0.35	0.15, 0.83	**0.02**	365.28	Model 2 vs. Model 1 LRT p-value 0.25
	TL vs. TH	1.36	0.64, 2.90	0.42		
	HR (pos vs. neg)	2.15	1.03, 4.45	**0.04**		
	Stage (III vs. II)	1.89	0.96, 3.71	0.07		
	pCR (pCR vs. RD)	0.22	0.10, 0.51	**<0.001**		
	HER2-E vs. not	4.9	2.21, 10.9	**<0.001**		
	TILs (continuous)	0.99	0.98, 1.01	0.27		
**Model 3**	THL vs. TH	0.34	0.14, 0.81	**0.015**	361.31	Model 3 vs. Model 1 LRT p-value **0.02**
	TL vs. TH	1.54	0.71, 3.33	0.27		
	HR (pos vs. neg)	1.71	0.81, 3.60	0.16		
	Stage (III vs. II)	1.95	0.99, 3.83	0.05		
	pCR (pCR vs. RD)	0.27	0.11, 0.62	**0.002**		
	HER2-E vs. not	4.6	2.10, 10.1	**<0.001**		
	Signature (cont)	0.67	0.47, 0.94	**0.02**		
**Model 4**	THL vs. TH	0.35	0.14, 0.83	**0.02**	363.27	Model 4 vs. Model 2 LRT p-value **0.04**
	TL vs. TH	1.55	0.72, 3.36	0.27		
	HR (pos vs. neg)	1.68	0.79, 3.59	0.18		
	Stage (III vs. II)	1.95	0.99, 3.85	0.05		
	pCR (pCR vs. RD)	0.26	0.11, 0.62	**0.002**		Model 4 vs. Model 3 LRT p-value 0.84
	HER2-E vs. not	4.55	2.07, 10.0	**<0.001**		

© 2023 Fernandez-Martinez A et al. *JAMA Oncology.*

	TILs (continuous)	1	0.98, 1.02	0.84		
	Signature (continuous)	0.65	0.43, 0.99	**0.04**		
**Signature: Plasma.cells ImSig.Nirmal CancerImmunolRes.2018 PMID.30266715**						
**Model**	**Features**	**HR**	**95% CI**	**p-value[1]**	**AIC**	**LR p-value[2]**
**Model 1**	THL vs. TH	0.37	0.16, 0.87	**0.02**	364.58	-
	TL vs. TH	1.34	0.63, 2.83	0.45		
	HR (pos vs. neg)	2.13	1.03, 4.42	**0.04**		
	Stage (III vs. II)	1.95	0.99, 3.83	0.05		
	pCR (pCR vs. RD)	0.2	0.09, 0.46	**<0.001**		
	HER2-E vs. Other	4.6	2.09, 10.1	**<0.001**		
**Model 2**	THL vs. TH	0.35	0.15, 0.83	**0.02**	365.28	Model 2 vs. Model 1 LRT p-value 0.25
	TL vs. TH	1.36	0.64, 2.90	0.42		
	HR (pos vs. neg)	2.15	1.03, 4.45	**0.04**		
	Stage (III vs. II)	1.89	0.96, 3.71	0.07		
	pCR (pCR vs. RD)	0.22	0.10, 0.51	**<0.001**		
	HER2-E vs. not	4.9	2.21, 10.9	**<0.001**		
	TILs (continuous)	0.99	0.98, 1.01	0.27		
**Model 3**	THL vs. TH	0.34	0.14, 0.80	**0.01**	360.66	Model 3 vs. Model 1 LRT p-value **0.02**
	TL vs. TH	1.61	0.74, 3.49	0.23		
	HR (pos vs. neg)	2.11	1.02, 4.35	**0.04**		
	Stage (III vs. II)	1.96	1.00, 3.86	0.05		
	pCR (pCR vs. RD)	0.28	0.12, 0.66	**0.004**		
	HER2-E vs. not	5.58	2.47, 12.6	**<0.001**		
	Signature (cont)	0.64	0.45, 0.91	**0.01**		
**Model 4**	THL vs. TH	0.34	0.14, 0.82	**0.02**	362.61	Model 4 vs. Model 2 LRT p-value **0.03**
	TL vs. TH	1.62	0.74, 3.53	0.22		
	HR (pos vs. neg)	2.11	1.02, 4.34	**0.04**		
	Stage (III vs. II)	1.97	1.00, 3.89	0.05		
	pCR (pCR vs. RD)	0.28	0.12, 0.66	**0.004**		Model 4 vs. Model 3 LRT p-value 0.81
	HER2-E vs. not	5.59	2.47, 12.6	**<0.001**		

© 2023 Fernandez-Martinez A et al. *JAMA Oncology.*

	TILs (continuous)	1	0.98, 1.02	0.81		
	Signature (continuous)	0.62	0.41, 0.95	**0.03**		
**Signature: Bcells.Plasma.cells.52genes Miller GenomeBiol.2013 PMID.23618380**						
**Model**	**Features**	**HR**	**95% CI**	**p-value[1]**	**AIC**	**LR p-value[2]**
**Model 1**	THL vs. TH	0.37	0.16, 0.87	**0.02**	364.58	-
	TL vs. TH	1.34	0.63, 2.83	0.45		
	HR (pos vs. neg)	2.13	1.03, 4.42	**0.04**		
	Stage (III vs. II)	1.95	0.99, 3.83	0.05		
	pCR (pCR vs. RD)	0.2	0.09, 0.46	**<0.001**		
	HER2-E vs. Other	4.6	2.09, 10.1	**<0.001**		
**Model 2**	THL vs. TH	0.35	0.15, 0.83	**0.02**	365.28	Model 2 vs. Model 1 LRT p-value 0.25
	TL vs. TH	1.36	0.64, 2.90	0.42		
	HR (pos vs. neg)	2.15	1.03, 4.45	**0.04**		
	Stage (III vs. II)	1.89	0.96, 3.71	0.07		
	pCR (pCR vs. RD)	0.22	0.10, 0.51	**<0.001**		
	HER2-E vs. not	4.9	2.21, 10.9	**<0.001**		
	TILs (continuous)	0.99	0.98, 1.01	0.27		
**Model 3**	THL vs. TH	0.33	0.14, 0.78	**0.01**	357.02	Model 3 vs. Model 1 LRT p-value **0.002**
	TL vs. TH	1.68	0.78, 3.64	0.19		
	HR (pos vs. neg)	1.62	0.77, 3.38	0.20		
	Stage (III vs. II)	2.1	1.07, 4.13	**0.03**		
	pCR (pCR vs. RD)	0.3	0.13, 0.71	**0.006**		
	HER2-E vs. not	5	2.27, 11.0	**<0.001**		
	Signature (cont)	0.56	0.39, 0.81	**0.002**		
**Model 4**	THL vs. TH	0.34	0.14, 0.82	**0.02**	358.57	Model 4 vs. Model 2 LRT p-value **0.003**
	TL vs. TH	1.74	0.80, 3.81	0.16		
	HR (pos vs. neg)	1.56	0.74, 3.27	0.239		
	Stage (III vs. II)	2.15	1.09, 4.24	**0.03**		
	pCR (pCR vs. RD)	0.29	0.12, 0.69	**0.005**		Model 4 vs. Model 3 LRT p-value 0.51
	HER2-E vs. not	4.89	2.21, 10.8	**<0.001**		

© 2023 Fernandez-Martinez A et al. *JAMA Oncology.*

	TILs (continuous)	1.01	0.99, 1.03	0.50		
	Signature (continuous)	0.52	0.34, 0.79	**0.002**		
**Signature: Ig TCGA.BRCA.1198 Cell.2015 PMID.26451490**						
**Model**	**Features**	**HR**	**95% CI**	**p-value[1]**	**AIC**	**LR p-value[2]**
**Model 1**	THL vs. TH	0.37	0.16, 0.87	**0.02**	364.58	-
	TL vs. TH	1.34	0.63, 2.83	0.45		
	HR (pos vs. neg)	2.13	1.03, 4.42	**0.04**		
	Stage (III vs. II)	1.95	0.99, 3.83	0.05		
	pCR (pCR vs. RD)	0.2	0.09, 0.46	**<0.001**		
	HER2-E vs. Other	4.6	2.09, 10.1	**<0.001**		
**Model 2**	THL vs. TH	0.35	0.15, 0.83	**0.02**	365.28	Model 2 vs. Model 1 LRT p-value 0.25
	TL vs. TH	1.36	0.64, 2.90	0.42		
	HR (pos vs. neg)	2.15	1.03, 4.45	**0.04**		
	Stage (III vs. II)	1.89	0.96, 3.71	0.07		
	pCR (pCR vs. RD)	0.22	0.10, 0.51	**<0.001**		
	HER2-E vs. not	4.9	2.21, 10.9	**<0.001**		
	TILs (continuous)	0.99	0.98, 1.01	0.27		
**Model 3**	THL vs. TH	0.35	0.15, 0.83	**0.02**	356.98	Model 3 vs. Model 1 LRT p-value **0.002**
	TL vs. TH	1.81	0.83, 3.95	0.14		
	HR (pos vs. neg)	1.73	0.84, 3.59	0.14		
	Stage (III vs. II)	2.21	1.12, 4.37	**0.02**		
	pCR (pCR vs. RD)	0.31	0.13, 0.72	**0.007**		
	HER2-E vs. not	5.06	2.30, 11.1	**<0.001**		
	Signature (cont)	0.56	0.39, 0.81	**0.002**		
**Model 4**	THL vs. TH	0.36	0.15, 0.87	**0.02**	358.58	Model 4 vs. Model 2 LRT p-value **0.003**
	TL vs. TH	1.89	0.85, 4.17	0.12		
	HR (pos vs. neg)	1.7	0.82, 3.51	0.15		
	Stage (III vs. II)	2.29	1.15, 4.56	**0.02**		
	pCR (pCR vs. RD)	0.3	0.13, 0.71	**0.006**		Model 4 vs. Model 3 LRT p-value 0.53
	HER2-E vs. not	4.97	2.25, 11.0	**<0.001**		

© 2023 Fernandez-Martinez A et al. *JAMA Oncology.*

	TILs (continuous)	1.01	0.99, 1.02	0.52		
	Signature (continuous)	0.52	0.34, 0.80	**0.003**		
**Signature: Immune1 TCGA.BRCA.1198 JCI.2020 PMID.32573490**						
**Model**	**Features**	**HR**	**95% CI**	**p-value[1]**	**AIC**	**LR p-value[2]**
**Model 1**	THL vs. TH	0.37	0.16, 0.87	**0.02**	364.58	-
	TL vs. TH	1.34	0.63, 2.83	0.45		
	HR (pos vs. neg)	2.13	1.03, 4.42	**0.04**		
	Stage (III vs. II)	1.95	0.99, 3.83	0.05		
	pCR (pCR vs. RD)	0.2	0.09, 0.46	**<0.001**		
	HER2-E vs. Other	4.6	2.09, 10.1	**<0.001**		
**Model 2**	THL vs. TH	0.35	0.15, 0.83	**0.02**	365.28	Model 2 vs. Model 1 LRT p-value 0.25
	TL vs. TH	1.36	0.64, 2.90	0.42		
	HR (pos vs. neg)	2.15	1.03, 4.45	**0.04**		
	Stage (III vs. II)	1.89	0.96, 3.71	0.07		
	pCR (pCR vs. RD)	0.22	0.10, 0.51	**<0.001**		
	HER2-E vs. not	4.9	2.21, 10.9	**<0.001**		
	TILs (continuous)	0.99	0.98, 1.01	0.27		
**Model 3**	THL vs. TH	0.34	0.14, 0.80	**0.01**	360.55	Model 3 vs. Model 1 LRT p-value **0.01**
	TL vs. TH	1.59	0.74, 3.45	0.24		
	HR (pos vs. neg)	1.73	0.83, 3.63	0.14		
	Stage (III vs. II)	2.02	1.02, 3.97	**0.04**		
	pCR (pCR vs. RD)	0.28	0.12, 0.66	**0.004**		
	HER2-E vs. not	4.73	2.16, 10.4	**<0.001**		
	Signature (cont)	0.64	0.44, 0.91	**0.01**		
**Model 4**	THL vs. TH	0.34	0.14, 0.83	**0.02**	362.40	Model 4 vs. Model 2 LRT p-value **0.03**
	TL vs. TH	1.62	0.75, 3.52	0.22		
	HR (pos vs. neg)	1.69	0.80, 3.57	0.17		
	Stage (III vs. II)	2.04	1.03, 4.04	**0.04**		
	pCR (pCR vs. RD)	0.28	0.12, 0.66	**0.003**		Model 4 vs. Model 3 LRT p-value 0.70
	HER2-E vs. not	4.64	2.11, 10.2	**<0.001**		

© 2023 Fernandez-Martinez A et al. *JAMA Oncology.*

	TILs (continuous)	1	0.98, 1.02	0.70		
	Signature (continuous)	0.6	0.39, 0.94	**0.03**		
**Signature: IgG Rody BreastCancerResearch.2008 PMID.19272155**						
**Model**	**Features**	**HR**	**95% CI**	**p-value[1]**	**AIC**	**LR p-value[2]**
**Model 1**	THL vs. TH	0.37	0.16, 0.87	**0.02**	364.58	-
	TL vs. TH	1.34	0.63, 2.83	0.45		
	HR (pos vs. neg)	2.13	1.03, 4.42	**0.04**		
	Stage (III vs. II)	1.95	0.99, 3.83	0.05		
	pCR (pCR vs. RD)	0.2	0.09, 0.46	**<0.001**		
	HER2-E vs. Other	4.6	2.09, 10.1	**<0.001**		
**Model 2**	THL vs. TH	0.35	0.15, 0.83	**0.02**	365.28	Model 2 vs. Model 1 LRT p-value 0.25
	TL vs. TH	1.36	0.64, 2.90	0.42		
	HR (pos vs. neg)	2.15	1.03, 4.45	**0.04**		
	Stage (III vs. II)	1.89	0.96, 3.71	0.07		
	pCR (pCR vs. RD)	0.22	0.10, 0.51	**<0.001**		
	HER2-E vs. not	4.9	2.21, 10.9	**<0.001**		
	TILs (continuous)	0.99	0.98, 1.01	0.27		
**Model 3**	THL vs. TH	0.36	0.15, 0.86	**0.02**	359.43	Model 3 vs. Model 1 LRT p-value **0.007**
	TL vs. TH	1.69	0.78, 3.67	0.18		
	HR (pos vs. neg)	1.79	0.86, 3.71	0.12		
	Stage (III vs. II)	2.19	1.11, 4.31	**0.02**		
	pCR (pCR vs. RD)	0.28	0.12, 0.65	**0.003**		
	HER2-E vs. not	4.97	2.26, 10.9	**<0.001**		
	Signature (cont)	0.61	0.43, 0.87	**0.007**		
**Model 4**	THL vs. TH	0.37	0.15, 0.88	**0.03**	361.35	Model 4 vs. Model 2 LRT p-value **0.02**
	TL vs. TH	1.72	0.79, 3.75	0.17		
	HR (pos vs. neg)	1.77	0.85, 3.68	0.13		
	Stage (III vs. II)	2.22	1.11, 4.42	**0.02**		
	pCR (pCR vs. RD)	0.28	0.12, 0.65	**0.003**		Model 4 vs. Model 3 LRT p-value 0.78
	HER2-E vs. not	4.93	2.24, 10.9	**<0.001**		

© 2023 Fernandez-Martinez A et al. *JAMA Oncology.*

	TILs (continuous)	1	0.98, 1.02	0.78		
	Signature (continuous)	0.59	0.39, 0.90	**0.01**		

CI: confident intervals; EFS: event-free survival; HR: hormone receptor; pCR: in-breast pathologic complete response; RD: residual disease; pos: positive; neg: negative; HER2-E: HER2-Enriched; AIC: Akaike Information Criterion; LR: likelihood-ratio test; TILs: tumor infiltrating lymphocytes; IgG: immunoglobulin G; T: weekly paclitaxel; H: trastuzumab; L: lapatinib. [1]Cox regression model p-value; [2]Likelihood-ratio test p-value.

© 2023 Fernandez-Martinez A et al. *JAMA Oncology.*

eTable 11. Summary Table of Akaike Information Criteria (AIC) and C-Index From Multivariable Cox Models Including Immune Gene Expression Signatures (iGES)

iGES ID	AIC	c-index
IGG.Cluster Fan BMCMedGenomics.2011 PMID.21214954	389.97	0.76
Plasma.cells ImSig.Nirmal CancerImmunolRes.2018 PMID.30266715	388.34	0.73
Bcells.Plasma.cells.52genes Miller GenomeBiol.2013 PMID.23618380	385.04	0.77
Ig TCGA.BRCA.1198 Cell.2015 PMID.26451490	384.82	0.77
Immune1 TCGA.BRCA.1198 JCI.2020 PMID.32573490	389.43	0.74
IgG Rody BreastCancerResearch.2008 PMID.19272155	387.85	0.74

A median c-index was calculated for each model using 5-folds cross validation.

© 2023 Fernandez-Martinez A et al. *JAMA Oncology.*

eReferences

1. Andres-Terre M, McGuire HM, Pouliot Y, et al: Integrated, Multi-cohort Analysis Identifies Conserved Transcriptional Signatures across Multiple Respiratory Viruses. Immunity 43:1199-211, 2015

2. Beck AH, Espinosa I, Edris B, et al: The macrophage colony-stimulating factor 1 response signature in breast carcinoma. Clin Cancer Res 15:778-87, 2009

3. Bindea G, Mlecnik B, Tosolini M, et al: Spatiotemporal dynamics of intratumoral immune cells reveal the immune landscape in human cancer. Immunity 39:782-95, 2013

4. Broz ML, Binnewies M, Boldajipour B, et al: Dissecting the tumor myeloid compartment reveals rare activating antigen-presenting cells critical for T cell immunity. Cancer Cell 26:638-52, 2014

5. Cabrita R, Lauss M, Sanna A, et al: Tertiary lymphoid structures improve immunotherapy and survival in melanoma. Nature 577:561-565, 2020

6. Calabro A, Beissbarth T, Kuner R, et al: Effects of infiltrating lymphocytes and estrogen receptor on gene expression and prognosis in breast cancer. Breast Cancer Res Treat 116:69-77, 2009

7. Chang HY, Sneddon JB, Alizadeh AA, et al: Gene expression signature of fibroblast serum response predicts human cancer progression: similarities between tumors and wounds. PLoS Biol 2:E7, 2004

8. Charoentong P, Finotello F, Angelova M, et al: Pan-cancer Immunogenomic Analyses Reveal Genotype-Immunophenotype Relationships and Predictors of Response to Checkpoint Blockade. Cell Rep 18:248-262, 2017

9. Ciriello G, Gatza ML, Beck AH, et al: Comprehensive Molecular Portraits of Invasive Lobular Breast Cancer. Cell 163:506-19, 2015

10. Cristescu R, Mogg R, Ayers M, et al: Pan-tumor genomic biomarkers for PD-1 checkpoint blockade-based immunotherapy. Science 362, 2018

11. Dybkaer K, Bogsted M, Falgreen S, et al: Diffuse large B-cell lymphoma classification system that associates normal B-cell subset phenotypes with prognosis. J Clin Oncol 33:1379-88, 2015

12. Fan C, Prat A, Parker JS, et al: Building prognostic models for breast cancer patients using clinical variables and hundreds of gene expression signatures. BMC Med Genomics 4:3, 2011

13. Forero A, Li Y, Chen D, et al: Expression of the MHC Class II Pathway in Triple-Negative Breast Cancer Tumor Cells Is Associated with a Good Prognosis and Infiltrating Lymphocytes. Cancer Immunol Res 4:390-9, 2016

14. Garber ME, Saldanha A, Parker JS, et al: A B-Cell Gene Signature Correlates With the Extent of Gluten-Induced Intestinal Injury in Celiac Disease. Cell Mol Gastroenterol Hepatol 4:1-17, 2017

15. Garcia-Recio S, Thennavan A, East MP, et al: FGFR4 regulates tumor subtype differentiation in luminal breast cancer and metastatic disease. J Clin Invest 130:4871-4887, 2020

16. Gatza ML, Lucas JE, Barry WT, et al: A pathway-based classification of human breast cancer. Proc Natl Acad Sci U S A 107:6994-9, 2010

17. Ghassabeh GH, De Baetselier P, Brys L, et al: Identification of a common gene signature for type II cytokine-associated myeloid cells elicited in vivo in different pathologic conditions. Blood 108:575-83, 2006

18. Helmink BA, Reddy SM, Gao J, et al: B cells and tertiary lymphoid structures promote immunotherapy response. Nature 577:549-555, 2020

19. Heng YJ, Lester SC, Tse GM, et al: The molecular basis of breast cancer pathological phenotypes. J Pathol 241:375-391, 2017

20. Hollern DP, Xu N, Thennavan A, et al: B Cells and T Follicular Helper Cells Mediate Response to Checkpoint Inhibitors in High Mutation Burden Mouse Models of Breast Cancer. Cell 179:1191-1206 e21, 2019

21. Iglesia MD, Vincent BG, Parker JS, et al: Prognostic B-cell signatures using mRNA-seq in patients with subtype-specific breast and ovarian cancer. Clin Cancer Res 20:3818-29, 2014

22. Janiszewska M, Tabassum DP, Castano Z, et al: Subclonal cooperation drives metastasis by modulating local and systemic immune microenvironments. Nat Cell Biol 21:879-888, 2019

23. Kardos J, Chai S, Mose LE, et al: Claudin-low bladder tumors are immune infiltrated and actively immune suppressed. JCI Insight 1:e85902, 2016

24. Lauss M, Donia M, Harbst K, et al: Mutational and putative neoantigen load predict clinical benefit of adoptive T cell therapy in melanoma. Nat Commun 8:1738, 2017

25. Lin W, Cerny D, Chua E, et al: Human regulatory B cells combine phenotypic and genetic hallmarks with a distinct differentiation fate. J Immunol 193:2258-66, 2014

26. Nagalla S, Chou JW, Willingham MC, et al: Interactions between immunity, proliferation and molecular subtype in breast cancer prognosis. Genome Biol 14:R34, 2013

27. Newman AM, Liu CL, Green MR, et al: Robust enumeration of cell subsets from tissue expression profiles. Nat Methods 12:453-7, 2015

28. Nirmal AJ, Regan T, Shih BB, et al: Immune Cell Gene Signatures for Profiling the Microenvironment of Solid Tumors. Cancer Immunol Res 6:1388-1400, 2018

29. Pare L, Pascual T, Segui E, et al: Association between PD1 mRNA and response to anti-PD1 monotherapy across multiple cancer types. Ann Oncol 29:2121-2128, 2018

30. Pauken KE, Sammons MA, Odorizzi PM, et al: Epigenetic stability of exhausted T cells limits durability of reinvigoration by PD-1 blockade. Science 354:1160-1165, 2016

31. Perez EA, Thompson EA, Ballman KV, et al: Genomic analysis reveals that immune function genes are strongly linked to clinical outcome in the North Central Cancer Treatment Group n9831 Adjuvant Trastuzumab Trial. J Clin Oncol 33:701-8, 2015

32. Petitprez F, de Reynies A, Keung EZ, et al: B cells are associated with survival and immunotherapy response in sarcoma. Nature 577:556-560, 2020

33. Rody A, Holtrich U, Pusztai L, et al: T-cell metagene predicts a favorable prognosis in estrogen receptor-negative and HER2-positive breast cancers. Breast Cancer Res 11:R15, 2009

34. Rooney MS, Shukla SA, Wu CJ, et al: Molecular and genetic properties of tumors associated with local immune cytolytic activity. Cell 160:48-61, 2015

© 2023 Fernandez-Martinez A et al. *JAMA Oncology.*

35. Savas P, Virassamy B, Ye C, et al: Single-cell profiling of breast cancer T cells reveals a tissue-resident memory subset associated with improved prognosis. Nat Med 24:986-993, 2018

36. Schlecker E, Stojanovic A, Eisen C, et al: Tumor-infiltrating monocytic myeloid-derived suppressor cells mediate CCR5-dependent recruitment of regulatory T cells favoring tumor growth. J Immunol 189:5602-11, 2012

37. Shay T, Jojic V, Zuk O, et al: Conservation and divergence in the transcriptional programs of the human and mouse immune systems. Proc Natl Acad Sci U S A 110:2946-51, 2013

38. Subramanian A, Tamayo P, Mootha VK, et al: Gene set enrichment analysis: a knowledge-based approach for interpreting genome-wide expression profiles. Proc Natl Acad Sci U S A 102:15545-50, 2005

39. Thorsson V, Gibbs DL, Brown SD, et al: The Immune Landscape of Cancer. Immunity 48:812-830 e14, 2018

40. Wolf DM, Lenburg ME, Yau C, et al: Gene co-expression modules as clinically relevant hallmarks of breast cancer diversity. PLoS One 9:e88309, 2014

41. Yoshihara K, Shahmoradgoli M, Martinez E, et al: Inferring tumour purity and stromal and immune cell admixture from expression data. Nat Commun 4:2612, 2013

42. Youn JI, Collazo M, Shalova IN, et al: Characterization of the nature of granulocytic myeloid-derived suppressor cells in tumor-bearing mice. J Leukoc Biol 91:167-81, 2012

43. Zhu G, Falahat R, Wang K, et al: Tumor-Associated Tertiary Lymphoid Structures: Gene-Expression Profiling and Their Bioengineering. Front Immunol 8:767, 2017

© 2023 Fernandez-Martinez A et al. *JAMA Oncology.*
